# Screening to prevent fragility fractures among adults 40 years and older in primary care: protocol for a systematic review

**DOI:** 10.1186/s13643-019-1094-5

**Published:** 2019-08-23

**Authors:** Michelle Gates, Jennifer Pillay, Guylène Thériault, Heather Limburg, Roland Grad, Scott Klarenbach, Christina Korownyk, Donna Reynolds, John J. Riva, Brett D. Thombs, Gregory A. Kline, William D. Leslie, Susan Courage, Ben Vandermeer, Robin Featherstone, Lisa Hartling

**Affiliations:** 1 Alberta Research Centre for Health Evidence, University of Alberta, 11405 87 Avenue, Edmonton, Alberta T6G 1C9 Canada; 2 Department of Family Medicine, McGill University, Montreal, Canada; 3 Global Health and Guidelines Division, Public Health Agency of Canada, Ottawa, Canada; 4 Department of Medicine, University of Alberta, Edmonton, Canada; 5 Department of Family and Community Medicine, University of Toronto, Toronto, Canada; 6 Department of Family Medicine, McMaster University, Hamilton, Canada; 7 Department of Health Research Methods, Evidence and Impact, McMaster University, Hamilton, Canada; 8 Faculty of Medicine, McGill University and Lady Davis Institute for Medical Research, Jewish General Hospital, Montreal, Canada; 9 Department of Medicine, University of Calgary, Calgary, Canada; 10 Department of Medicine (Endocrinology), University of Manitoba, Winnipeg, Canada; 11 Department of Radiology (Nuclear Medicine), University of Manitoba, Winnipeg, Canada

**Keywords:** Systematic review, Guideline, Fragility fractures, Screening

## Abstract

**Purpose:**

To inform recommendations by the Canadian Task Force on Preventive Health Care by systematically reviewing direct evidence on the effectiveness and acceptability of screening adults 40 years and older in primary care to reduce fragility fractures and related mortality and morbidity, and indirect evidence on the accuracy of fracture risk prediction tools. Evidence on the benefits and harms of pharmacological treatment will be reviewed, if needed to meaningfully influence the Task Force’s decision-making.

**Methods:**

A modified update of an existing systematic review will evaluate screening effectiveness, the accuracy of screening tools, and treatment benefits. For treatment harms, we will integrate studies from existing systematic reviews. A de novo review on acceptability will be conducted. Peer-reviewed searches (Medline, Embase, Cochrane Library, PsycINFO [acceptability only]), grey literature, and hand searches of reviews and included studies will update the literature. Based on pre-specified criteria, we will screen studies for inclusion following a liberal-accelerated approach. Final inclusion will be based on consensus. Data extraction for study results will be performed independently by two reviewers while other data will be verified by a second reviewer; there may be some reliance on extracted data from the existing reviews. The risk of bias assessments reported in the existing reviews will be verified and for new studies will be performed independently. When appropriate, results will be pooled using either pairwise random effects meta-analysis (screening and treatment) or restricted maximum likelihood estimation with Hartun-Knapp-Sidnick-Jonkman correction (risk prediction model calibration). Subgroups of interest to explain heterogeneity are age, sex, and menopausal status. Two independent reviewers will rate the certainty of evidence using the GRADE approach, with consensus reached for each outcome rated as critical or important by the Task Force.

**Discussion:**

Since the publication of other guidance in Canada, new trials have been published that are likely to improve understanding of screening in primary care settings to prevent fragility fractures. A systematic review is required to inform updated recommendations that align with the current evidence base.

**Electronic supplementary material:**

The online version of this article (10.1186/s13643-019-1094-5) contains supplementary material, which is available to authorized users.

## Background

In this review, we will synthesize evidence related to screening to prevent fragility fractures and related mortality and morbidity among adults 40 years and older in primary care. The findings will be used by the Canadian Task Force on Preventive Health Care—supplemented by consultations with patients on outcome prioritization and by information from organizational stakeholders and other sources on issues of feasibility, acceptability, costs/resources, and equity―to inform recommendations on screening for the prevention of fragility fractures among adults 40 years and older, which will support primary care providers in delivering preventive care.

### Rationale and scope of systematic review

Osteoporosis Canada’s 2010 Clinical Practice Guideline for the Diagnosis and Management of Osteoporosis is the guideline commonly used for prevention of fragility fractures among Canadian adults [1]. The Osteoporosis Canada guideline recommends that all adults over 50 years be assessed for risk factors for osteoporosis and fragility fracture [1]. Adults 65 years and older, menopausal women, and men aged 50 to 64 years with clinical risk factors are recommended to have bone mineral density (BMD) assessed using dual-energy x-ray absorptiometry (DXA) [1]. Osteoporosis Canada recommends that one of two closely related risk assessment tools validated in the Canadian population be used to estimate absolute fracture risk [1]: the Canadian Association of Radiologists and Osteoporosis Canada risk assessment tool (CAROC) [2] or the Fracture Risk Assessment Tool (FRAX) [3]. Since publication of the Osteoporosis Canada guideline, new evidence has become available, including results from recent trials of screening in primary care settings to prevent fragility fractures [4, 5]. Evidence from screening trials is likely to improve understanding of the effects of screening, but as far as we are aware, no systematic review has included these newer trials.

Prevention of fragility fractures has traditionally focused on BMD measurement with intervention after findings of low bone mass or osteoporosis [6]. However, most fractures occur in individuals with a BMD not meeting the diagnostic threshold for osteoporosis [7, 8], and this poor sensitivity suggests that BMD alone may not be the ideal strategy for population screening when the outcome of interest is the detection of persons at high risk in order to prevent future fracture [6]. Improving the predictive value for future fracture risk (and therefore detection of patients who stand to benefit from intervention), by focusing on other clinical risk factors, or by combining these with BMD assessments, has shown promise and resulted in the development of several fracture risk prediction tools that offer short- to mid-term absolute fracture risks. As evidenced by the increasing integration of FRAX and other risk assessment tools into clinical practice guidelines [3, 9], for many, the concept of screening for osteoporosis has been replaced with that of screening to prevent fragility fracture. Though the Osteoporosis Canada Guideline [1] and other Canadian guidelines [10, 11] now recommend that absolute fracture risk be estimated using an assessment tool incorporating clinical risk factors, with BMD measurement if indicated, practice may vary across clinical settings [12–15], and the impact of this strategy on fracture incidence or other patient-important outcomes—particularly across all patient groups—is uncertain. There is no international consensus on the recommended approach to screening to prevent fragility fractures [9]. Among other factors, this lack of guidance has contributed to a limited uptake of risk assessment tools in clinical practice [13, 16]. As a result, there is a sizable gap between best practice recommendations and the fracture prevention and management services offered to Canadians [17].

The focus of this systematic review will be on screening for prevention of fragility fractures in the general primary care adult population aged 40 years and greater. The 40-year age cut-off was chosen taking into account the increasing risk of fracture with advancing age [18] and to ensure that women in early menopause (e.g., 40 to 45 years) would be captured. Prevention of subsequent fractures among those known to have previously experienced a clinical fragility fracture will not be examined, because there is little uncertainty and large consensus regarding the appropriate management of these patients [19–22].

### Description of the condition and disease burden

Fragility fractures are those that occur spontaneously during normal daily activities or that result from minor impacts that would not normally cause a fracture in healthy adults [17]. Major independent risk factors for fragility fracture include the use of certain medications (e.g., glucocorticoids), low body weight, smoking, alcohol use, family history of fracture, older age, female sex, history of falls, type 2 diabetes, and prior history of fragility fracture [23–28]. Age is a strong predictor of incident fractures, particularly among postmenopausal women and older men [18]. Findings from the Canadian Multicentre Osteoporosis Study indicate that the 10-year fracture risk is relatively low for men up to 65 years, while in women the risk increased with age (e.g., 6.7% in 35–44 years; 8.3% in 45–54 years; 13.9% in 55–65 years; 21.3% in 65-74 years; and 31.8% in 75–84 years) [18]. Compared to postmenopause, the occurrence of fragility fractures in premenopausal women is relatively rare [29, 30]. Osteoporosis, a state characterized by a loss of bone mass and reduced bone quality [31], is also an important risk factor for fragility fracture. According to the World Health Organization, individuals may be conventionally classified as having osteoporosis when they have a BMD T-score that is 2.5 or more standard deviations (SDs) below the mean for healthy young adults based on a standard reference site (e.g., the femoral neck) [31]. Osteoporosis may be a consequence of aging or secondary to other medical conditions or treatments [32].

Fragility fractures impose a substantial burden on Canadian society. The most recent published data from the 2010–2011 fiscal year indicates that Canadians 50 years of age and older sustained over 130,000 fragility fractures [33]. These resulted in a greater number of hospitalized days than either stroke or myocardial infarction [34]. The incidence of hip fractures in Canadians 40 years and older during 2015–2016 was 147 per 100,000, with rates in women over two times those in men and steep increases based on age after 40 years (e.g., 87 per 100,000 in 65–69 and 1156 per 100,000 in 85–89 year olds) [35]. The consequences of fragility fractures, particularly hip and clinical vertebral fractures, include significant morbidity (e.g., decreased mobility, pain, reduced quality of life) and an increased risk of mortality in the 5 years post-fracture [36–38]. For example, individuals 50 years or older who sustain a hip fracture are at 4.2 times (95% confidence interval (CI) 1.8 to 9.6) greater risk of mortality within the first year post-fracture as compared with those without fractures [37]. The cost of acute and long-term care, prescription drugs, and wage losses and home care for fragility fractures has been estimated at $4.6 billion (2010/11) [33]. Asymptomatic vertebral fractures rarely come to clinical attention [39, 40], but there is evidence to suggest they strongly predict future fracture [24, 41], and are associated with excess mortality [42, 43]. However, uncertainty regarding causality remains because many studies to date have not adjusted for important confounding variables such as frailty, other fractures (e.g., hip), and the presence of comorbid conditions [42, 43]. It is believed that excess mortality in those with vertebral fractures (radiographic or clinical) is predominantly related to comorbid conditions that predispose individuals both to fracture and to increased risk of mortality [40, 43, 44].

### Components of screening interventions

#### Rationale for screening

Since individuals without prior fracture but at risk for incident fragility fracture are asymptomatic, screening should be able to identify those who are at greater risk of fracture and potential candidates for preventive intervention. Information from screening may be used, along with patient values and preferences, to inform decisions about treatment that might decrease future risk of fracture and related morbidity [45]. Thus, the aim of screening is not to detect the existence of osteoporosis but rather to reduce fracture-related burden of morbidity, mortality, and costs.

Screening to prevent fragility fractures involves a sequence of activities, not simply one test. The activities include a systematic offering of screening in a specified population of asymptomatic people with the intent to identify those at increased risk for fractures in order to provide preventive treatment and improve health outcomes. The effectiveness is ideally measured over the entire population being offered the screening program, relying upon trials that directly evaluate long-term outcomes from screening compared with no screening, or between different screening programs, in primary care populations. Inferences about the effectiveness of screening programs to prevent fragility fractures, however, have mostly relied upon indirect data (linked evidence) from individual components of an end-to-end screening program. These indirect data include information about the accuracy and performance of risk assessment tools and the effectiveness of treatment among people at increased risk for fracture.

#### Fracture risk assessment

International guidelines (Additional file 1) vary in their current recommendations on screening approaches, based on the country-specific population burden of fragility fractures and mortality, competing societal priorities, and resource availability [9]. Several screening strategies exist in clinical practice, and in most cases, recommendations differ by population group based on sex, menopausal status, and age. For women 65 years or older (or postmenopausal), many North American organizations recommend either only using BMD assessment [46, 47] or assessing BMD in all women and integrating this with other clinical risk factors into an absolute fracture risk for treatment decision-making [1, 10, 12, 48]. More common in European guidelines for this population group (and oftentimes across all populations >50 years) is an assessment of absolute risk using clinical factors before deciding whether to further stratify risk by assessing BMD [49–51]. For women who are not menopausal (or < 65 years) and for men, many recommendations are to first assess risk based on clinical factors and use BMD in those considered at-risk. In some approaches, BMD assessment is also recommended in all men of a certain age category (e.g., ≥ 50 [12], ≥ 65 [1], or ≥ 70 years [52, 53]). Shared decision-making is incorporated in few recommendations; the Institute for Clinical Systems Improvement recommends shared decision-making about BMD testing, but only in specific population subgroups: men 70 years and older; adults with a known condition associated with low bone mass/bone loss; and organ transplant patients [54]. The European Society of Endocrinology’s guidelines for postmenopausal women recommend that patient values and preferences be considered when deciding who to treat [55]. When BMD testing follows a clinical risk assessment, it is not always clear if this is used independently or integrated (as possible) into a total clinical risk score. Moreover, in some jurisdictions, the indication for BMD testing may be restricted to instances where the absolute fracture risk is predicted to be intermediate to moderate (i.e., close to the level where treatment would be considered), whereby further information from the test may better inform treatment decisions. In these guidelines (e.g., United Kingdom), BMD testing would not be indicated when absolute risk is either well below or far above treatment thresholds [56]. The definition of the intermediate risk category may be determined based on other considerations such as resource availability and funding, and the risk profile of the target population.

There are at least 12 published tools to predict fracture risk [16, 19]. These tools combine an individual’s known clinical risk factors for fragility fracture into a single total estimation of absolute fracture risk over a certain time period (commonly 5 or 10 years) [16]. The main difference between various tools is the number of factors assessed and how these factors are weighted in the models. Certain prediction tools (e.g., FRAX) require calibration to the population context in which they will be used to account for differences in fracture incidence and mortality across geographic regions [57]. Not all tools have been validated in populations outside of their derivation cohort, limiting transferability of these risk prediction models [58]. Some tools (e.g., FRAX, Garvan) allow for, but do not require, inclusion of BMD results; others (e.g., CAROC) require BMD. Tools generally incorporate easily obtained clinical risk measures, but may be enhanced by simple arithmetic procedures (e.g., falls history or level of exposure to glucocorticoids added to FRAX [56]).

Most guidelines recommend that when BMD is assessed it should be measured at the femoral neck via DXA [1, 19, 50, 59], because measurements at this site can be incorporated into many risk assessment tools [1, 19, 50, 59], and the use of multiple sites does not appear to improve the accuracy of fracture prediction [60, 61]. Lumbar spine BMD is also commonly reported and may be used by some practitioners in their decision-making on fracture risk assessment. For example, procedures have been developed and endorsed by the International Society for Clinical Densitometry and International Osteoporosis Foundation [62], to adjust FRAX probabilities when large discordance exists between lumbar spine and femoral neck BMD [63–65]. Some DXA instruments also offer vertebral fracture assessment, which can be used as a complement to BMD assessment to identify existing vertebral fractures [24]. Though these fractures are generally asymptomatic, clinicians should be aware that emerging evidence suggests that they strongly and independently predict incident clinical fracture outcomes (including hip fracture), independent of FRAX score [24, 41]. Further evidence, controlled for important confounding variables (e.g., hip fracture), is needed to confirm these findings. Current Canadian guidelines recommend vertebral fracture assessment via DXA or spine radiography when other clinical evidence suggests that a vertebral fracture is likely to be present (e.g., height loss) and may be used among those in moderate risk categories to help inform treatment decisions [1]. Analysis of data from the Canadian Multicentre Osteoporosis Study [66] indicates that Jiang et al.’s algorithm-based qualitative approach [67], which focuses on depression of the vertebral endplate, is the preferred approach to defining vertebral fractures (compared to the widely used Genant semiquantitative method [68]). Other less common BMD assessment methods (e.g., quantitative ultrasound, peripheral DXA, quantitative computed tomography scan, bone turnover markers) are typically used outside the scope of a population-based primary screening program [19, 59, 69].

Many systematic reviews on fracture risk assessment tools have focused on discrimination (i.e., ability to distinguish between people who develop fractures versus those who do not; measured by area under the receiver operating characteristics curve and other accuracy measures [e.g., sensitivity, specificity] relying on particular thresholds) as their primary, or only, outcome. On the other hand, primary care providers and patients may find calibration (i.e., accuracy of absolute risk prediction within a population) to be a more clinically meaningful measure to inform shared decisions about management.

#### Treatment thresholds and decisions

Treatment thresholds vary considerably across countries and may take into account variation in population-specific risk of fracture and mortality [57], competing health care priorities, patient willingness-to-pay for fracture-related health care, resource availability (e.g., access to BMD assessment tools), and pre-existing reimbursement criteria [9, 56]. The United States National Osteoporosis Foundation [70] recommends initiating pharmacological treatment in individuals with osteoporosis or with low BMD (T-score between − 1.0 and − 2.5, osteopenia) and either a 10-year hip fracture probability ≥ 3% or a 10-year major osteoporosis-related fracture probability ≥ 20% (using FRAX). This decision was supported by a cost-effectiveness analysis based on assumptions from one-step BMD screening followed by treatment with a generic bisphosphonate (assumed relative fracture reduction of 35%), and a willingness-to-pay threshold of $60,000 per quality-adjusted life-year gained [71, 72].

Canadian guidelines [1, 73], as well as those developed in several other countries (e.g., Austria [74], Greece [75], Hungary [76], Malaysia [77, 78], Mexico [79], the Philippines [80], Saudi Arabia [81], Poland [82], Slovakia [83], Slovenia [84], Spain [85–87], Taiwan [88], Thailand [89]), that are based on country-specific FRAX models, use a fixed 20% 10-year probability of major osteoporotic fracture as a treatment threshold [56]. In many (but not all) cases, the choice of the 20% intervention threshold is without a specific rationale, but instead based on the threshold used in the United States. Some guidelines also use a fixed 3% 10-year hip fracture probability as an alternative intervention threshold [56]. Another less common approach is to use intervention thresholds that increase with age [56]. The threshold is based on the rationale that because individuals with a prior fracture can be considered for treatment without the need for further assessment, other individuals of the same age with a similar fracture risk but no prior fracture should also be eligible [51]. Recent strategies adopt a hybrid approach (i.e., incorporating both fixed and age-dependent intervention thresholds) [51, 90, 91]. For example, the National Osteoporosis Guideline Group for the United Kingdom recommends that the treatment threshold increase with age for individuals up to 70 years to align with the level of risk associated with a prior fracture (ranges from approximately 7 to 24% 10-year probability of fracture; equivalent to the risk probability of a woman of the same age with a prior fragility fracture) [51]. After age 70, a fixed threshold is used to account for the reduced sensitivity of the risk probability algorithm for those without a prior fracture, which becomes most apparent at advanced age [51].

Treatment decisions may best be based on patient preferences, including their competing priorities and assessment of the relative importance of benefits and harms, and shared decision-making between patients and their healthcare providers [92]. Although treatment efficacy appears to be an important variable when choosing between different treatments [92], a major factor impacting the effectiveness of any treatment, and therefore screening program, is medication adherence. A study in the United States showed that close to 30% of patients provided with a prescription for osteoporosis treatment do not fill their prescription [93]. Of those initiating treatment, only half are still taking their medication at 1 year [94]. Predominant factors affecting adherence include dosing frequency, side effects of medications, costs, and lack of knowledge about the implications of osteoporosis [94]. One study conducted in the United States showed that in 2009, half of women (mean age 69 years; 30–40% with osteoporosis or prior fracture; perceived risk for 10-year fracture about 40%) who were provided information regarding fracture risks and treatment risks and benefits reported that they would accept prescription osteoporosis treatment at the threshold currently recommended by national physician treatment guidelines; 18% of the women would not accept treatment even at 50% fracture risk levels [95]. Willingness to accept treatment increased at higher levels of fracture risk and was higher in those with greater acceptance of the risks of medications [95]. There is large variation between patients regarding their treatment preferences, which supports a shared decision-making approach in place of recommended treatment thresholds based on fracture risk [92].

#### Pharmacological treatment

According to the 2010 Osteoporosis Canada guideline, for postmenopausal women, the first-line therapy is either one of three bisphosphonates (i.e., alendronate, risedronate or zoledronic acid), denosumab, or raloxifene (a selective estrogen receptor modulator) [1]. Hormone therapy may be considered for women experiencing vasomotor symptoms [1], and etidronate (another bisphosphonate) may be considered for those who are intolerant of first-line therapies [96]. As of October 2013, calcitonin is no longer approved by Health Canada for the treatment of osteoporosis due to concern about the increased risk of malignancies associated with the drug [97]. Moreover, systematic reviews evaluating etidronate have failed to demonstrate an impact on fracture reduction [19, 98] and this medication is used infrequently in Canada. For men, Osteoporosis Canada recommends bisphosphonates (i.e., alendronate, risedronate, zoledronic acid) as first-line therapy [1]. More recent guidelines from the American College of Physicians (2017) [99] and American Association of Clinical Endocrinologists/American College of Endocrinology (2016) [100] recommend alendronate, risedronate, zoledronic acid, and denosumab as first-line treatments for preventing fractures. Furthermore, use of hormone therapy for the prevention of fractures in postmenopausal women is not recommended [101].

In 2018, the United States Preventive Services Task Force (USPSTF) reviewed the effects of pharmacological treatments on preventing fragility fractures, using data from studies where the majority of the participants had no prior fracture [19]. Compared with placebo, moderate-certainty evidence was found for bisphosphonates in reducing the primary outcomes of vertebral and nonvertebral fractures in women, although low-certainty evidence found no difference in reducing the secondary outcome of hip fracture alone [19]. To explain this, it has been reported that only one of the three trials with hip fracture as an outcome was adequately powered to detect a significant difference [102]. Moreover, only one of the trials reporting on bisphosphonates was conducted in men [103]. One trial (*n* = 7868) of denosumab compared with placebo showed a decrease in vertebral, nonvertebral, and hip fractures in women [19]; the certainty of evidence was assessed as low for these outcomes. Few trials reported data on all clinical fractures or clinical vertebral fractures, and the reviewers did not assess the certainty of evidence for these outcomes. Trials have based their inclusion criteria on BMD (levels ranging from osteopenic to osteoporotic) rather than absolute risk for fractures, such that findings may not be applicable to those with high risk for fractures but with normal BMD. Similarly, beneficial effects may be obscured by inclusion of patients with low BMD but without higher fracture risk.

#### Non-pharmacological treatment

Non-pharmacological interventions (e.g., vitamin D, calcium, exercise, falls prevention) are considered as adjuncts to pharmacological treatment in primary care [1] and are considered to be out of scope for the current review.

### Negative consequences of screening and treatment

The development of recommendations for screening requires consideration of the potential for negative consequences (i.e., harms). These may be related to the screening test itself, such as radiation exposure from DXA, labelling (categorizing an individual as being “at-risk”), an inaccurate estimation of fracture risk, adverse effects related to pharmacological treatment, and overdiagnosis.

#### Screening tests and labelling

The screening tests may expose individuals to small amounts of radiation from DXA scans (with or without vertebral fracture assessment/spinal radiography) [104]. Costs for the patient and healthcare system include the time, effort, and expense related to attending appointments and the resources used to screen in clinical settings, to organize and perform tests, and to interpret results [19]. Patients may not always fully understand the meaning of risk assessment results, nor the consequences of an asymptomatic finding that cannot easily be conceptualized [105, 106]. Individuals undergoing screening, and those who perceive their predicted risk for fragility fracture to be high, may experience anxiety and feelings of uncertainty [105, 107]. These people may become overly cautious, limit their activities, and become less independent [107, 108]. They may feel stigmatized if they are labelled as “old” or “frail” [105]. However, quantitative data from a recent (*n* = 12,483) randomized controlled trial of screening in the United Kingdom examined the effect of the screening on anxiety and quality of life and suggested that the risk of these harms is small [4]. Individuals who were screened had levels of anxiety and quality of life that were very similar to those who were not screened [4]. One reason for this finding may be related to patient attitudes and beliefs. For example, a qualitative study of patients aged 50 and older in Canada showed that individuals perceived fractures and osteoporosis not to be serious health conditions and believed that they had negligible impact [109]. More research is needed to better understand the factors that influence a patient’s desire to have or avoid screening for osteoporosis-related fracture risk.

#### Inaccurate prediction of risk

Individuals can experience physical and psychological harm if their risk of fracture is over- or under-estimated (e.g., due to inaccurate measurement or interpretation of BMD or risk assessment results). When a patient is identified as having a higher risk of fracture than they truly have, they may experience unnecessary anxiety, and these individuals may be subjected to unneeded treatments that can have adverse effects with little or no benefit. Alternatively, a patient may be identified as having a lower risk of fracture than they truly have, which may be especially likely when BMD alone is used to estimate risk [110]. Based on false reassurance, these individuals may not make useful lifestyle modifications. They may also not have access to available treatments that could ultimately decrease their risk of fracture when screening program eligibility criteria are based on fracture risk rather than shared decision-making.

#### Adverse events associated with pharmacological treatment

Two systematic reviews have assessed adverse events for multiple bisphosphonates as well as for denosumab. Based on moderate-certainty evidence, the USPSTF’s 2018 systematic review did not find increased discontinuation rates due to the composite outcome “any adverse events,” upper gastrointestinal events, or serious adverse events for bisphosphonates over placebo. Insufficient evidence was found for cardiovascular events, osteonecrosis of the jaw, and atypical femoral fractures. For denosumab, in women, there was insufficient evidence for discontinuation due to adverse events, and low-certainty evidence found no significant increase in serious adverse events and serious infections [19]. The evidence used for this review was limited due to its focus on randomized controlled trials and studies of patients without previous fracture or secondary causes of osteoporosis, even though it may be argued that the harms of treatment are unlikely to differ substantially between somewhat different patient populations. Using a broader patient population, and thus a larger and more comprehensive evidence base, a 2012 systematic review by the Agency for Healthcare Research and Quality [94] reported different findings. For example, the review found high-certainty evidence for an increased risk of mild upper gastrointestinal events (e.g., acid reflux, nausea, vomiting) with alendronate, low-certainty evidence of an increased risk for bisphosphonate-related osteonecrosis of the jaw and atypical femoral fractures, and high-certainty evidence that denosumab increases infections [94]. Authors of both reviews considered the evidence insufficient for serious cardiovascular events (e.g., atrial fibrillation, acute coronary syndrome) and cancers (e.g., esophageal, gastrointestinal) [19, 94, 99]. For several outcomes (e.g., serious cardiovascular events), observational evidence was only considered when no trials existed. More recently, evidence has emerged to suggest the possibility of rapid bone loss or risk of multiple vertebral fractures due to rebound increased bone resorption after discontinuation of treatment with anti-RANKL antibodies (i.e., denosumab) [111]. However, supportive evidence of these effects from extensions of clinical trials is currently limited [112, 113].

#### Overdiagnosis

Although the result of the screening test—a risk for future fracture—is not a diagnosis of a condition or a disease, it has similar consequences because certain risk levels lead to labeling of patients as “at high risk,” and at one point a certain threshold has to be chosen by care providers either to serve as a threshold for treatment or to start a conversation with a patient about treatment. Overdiagnosed patients may be considered to be those who are deemed to be at excess risk of fracture—either according to a set threshold or based on shared decision-making—but who would never have known they were at risk because, without screening, they would not have experienced a fracture. Using a shared decision-making perspective, overdiagnosis leading to overtreatment may be conceptualized as patients who had a risk assessment and following shared decision-making decided to start treatment but would never have sustained a fragility fracture regardless of screening efforts.

## Methods

### Systematic review scope and approach

The Evidence Review and Synthesis Centre at the University of Alberta will conduct this review on behalf of the Task Force and following the research methods outlined in the Task Force methods manual [114]. We will follow a predefined protocol for the review (as documented herein), reported in accordance with the Preferred Reporting Items for Systematic reviews and Meta-Analysis Protocols statement (Additional file 2) [115]. During protocol development, a working group was formed consisting of Task Force members (GT, RG, SK, CK, DR, JR, BT), clinical experts (GK, WL), and scientific support from the Global Health and Guidelines Division at the Public Health Agency of Canada (HL, SC). The working group helped to formulate key questions (KQs) and PICOTS (population, interventions, comparators, outcomes, timing, and setting/study design) for the review, upon which the Task Force members made final decisions. Members of the Task Force rated outcomes based on their importance for clinical decision-making. The relative importance of the potential outcomes was also sought from patients, using surveys and focus groups conducted by the Knowledge Translation team at St. Michael’s Hospital (Toronto), and these findings were incorporated into the final outcome ratings of the Task Force. This version of the protocol was reviewed by seven external stakeholders and three peer-reviewers and was approved by the Task Force. It is registered with the International Prospective Registry of Systematic Reviews (PROSPERO) database (registration number forthcoming). We will record all protocol amendments (including description, timing within the review conduct, and reasoning) in the PROSPERO record and report these in the final manuscript. We will report our findings in accordance with the Preferred Reporting Items for Systematic reviews and Meta-Analyses statement [116] or the Checklist for the Critical Appraisal and Data Extraction for Systematic Reviews of Prediction Modelling Studies [58], as applicable to the research question. The Task Force and clinical experts will not be involved in the selection of studies, data extraction, or data analysis, but will help interpret the findings and comment on the draft report.

### Key questions and analytical framework

#### Key questions

KQ1a**:** What are the benefits and harms of screening compared with no screening to prevent fragility fractures and related morbidity and mortality in primary care for adults ≥ 40 years?

KQ1b: Does the effectiveness of screening to prevent fragility fractures vary by screening program type (i.e., 1 step vs 2 step) or risk assessment tool?

KQ2: How accurate are screening tests at predicting fractures among adults ≥ 40 years?

KQ3a: What are the benefits of pharmacologic treatments to prevent fragility fractures among adults ≥ 40 years?

KQ3b: What are the harms of pharmacologic treatments to prevent fragility fractures among adults ≥ 40 years?

KQ4: For patients ≥ 40 years, what is the acceptability* of screening and/or initiating treatment to prevent fragility fractures when considering the possible benefits and harms from screening and/or treatment?

*Acceptability indicators include positive attitudes, intentions, willingness, and uptake

**Fig. 1:**
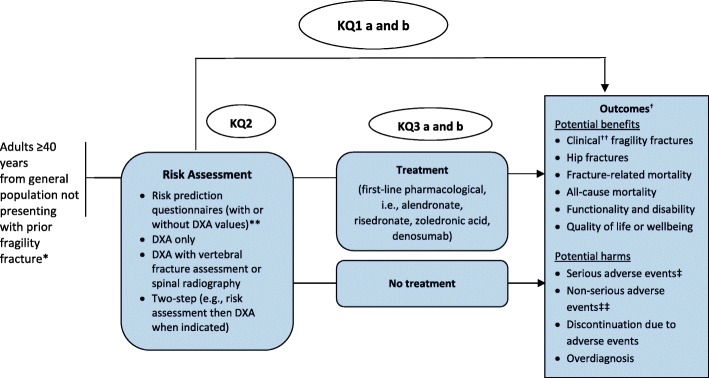
Analytical framework: Key question (KQ) 1a: What are the benefits and harms of screening compared with no screening to prevent fragility fractures and related morbidity and mortality in primary care for adults ≥ 40 years? KQ1b: Does the effectiveness of screening to prevent fragility fractures vary by screening program type (i.e., 1-step vs 2-step) or risk assessment tool? KQ2: How accurate are screening tests at predicting fracture risk among adults ≥ 40 years? KQ3a: What are the benefits of pharmacologic treatments to prevent fragility fractures among adults ≥ 40 years? KQ3b: What are the harms of pharmacologic treatments to prevent fragility fractures among adults ≥ 40 years? Abbreviations: DXA, dual-energy x-ray absorptiometry; KQ, key question *Main target population for guideline; inclusion and exclusion criteria for studies differ somewhat and are described in the text and Tables 1, 2, 3.** Any paper or electronic tool or set of questions using ≥ 2 demographic and/or clinical factors to assess risk for future fracture; must be externally validated for KQ2. ^†^These were all rated as critical or important by the Task Force, after considering input on their relative importance by patients, using surveys and focus groups conducted by the Knowledge Translation team at St. Michael’s Hospital (Toronto). All benefits are considered critical (rated as ≥ 7 on 9-point scale) except for all-cause mortality which was important (4–6 on 9-point scale); for harms, serious adverse events are critical while the others are important. We acknowledge that some outcomes, should the direction of effect be the opposite of intended, may be considered harms versus benefits, and vise versa. ^††^Any symptomatic and radiologically confirmed fracture (sites per author definition; may be defined as major osteoporotic fracture). ^‡^The primary outcome will be total count of any serious adverse event, but individual outcomes of (a) serious cardiovascular, (b) serious cardiac rhythm disturbances, (c) serious gastrointestinal events (except cancers), (d) gastrointestinal cancers (i.e., colon, colorectal, gastric, esophageal), (e) atypical fractures, and (f) osteonecrosis of the jaw will also be included. ^‡‡^ Count of total number of participants experiencing one or more non-serious adverse event; the outcome of “any adverse event” will be used as a surrogate if necessary

Figure 1 shows the analytical framework that depicts the population, KQs and outcomes, as well as key screening characteristics that will be considered. A staged approach to the evidence will be undertaken.

At the first stage, we will focus on identifying direct evidence from screening for fragility fracture on benefits and harms that are patient-oriented and either critical or important to clinical decision-making (KQ1a). We will prioritize evidence from randomized controlled trials, as these studies generally provide the highest internal validity. We will also consider evidence from controlled clinical trials (i.e., that includes a comparison [control] group and contains all of the key components of a true experimental design other than randomization: assignment of groups is determined by study design, and the administration of screening and endpoint ascertainment follows a protocol) if certainty in the evidence from randomized controlled trials is limited and poses a barrier to the development of recommendations, and the Task Force believes that further evidence from controlled clinical trials may influence their recommendations. We expect that this could occur due to limited available evidence overall or lack of evidence for selected subgroups (e.g., by age, sex, or different risk assessment approaches). If evidence for KQ1a indicates that screening for fragility fracture reduces fracture risk, we will examine whether this effectiveness varies by screening approach (e.g., 1 step vs. 2 step) or by risk assessment tool (KQ1b). We will review evidence related to the acceptability of screening and/or treatment (KQ4), as well as indirect evidence on the accuracy of screening tests (KQ2), concurrently with KQ1. We will proceed with KQ3 (treatment benefits and harms) only if the Task Force believes that further indirect evidence would influence their recommendations.

### Eligibility criteria

Tables 1, 2, 3, 4 show the inclusion and exclusion criteria for each key question, related to the population, intervention, comparator, outcomes, timing, and setting/study design (i.e., PICOTS). Additional file 3 contains a more detailed narrative description of the selection criteria.

**Table 1:** Key question 1 (benefits and harms of screening) study eligibility criteria

Criteria	Include	Exclude
Population	Asymptomatic adults ≥ 40 years in the general population (we will include studies where ≥ 80% of the sample or the sample mean age − 1 standard deviation is ≥ 40 years) *Subgroups for decision-making*: age, sex, menopausal status *Methods subgroups*: diabetes, presence of prior fractures, baseline predicted fracture risk, length of follow-up	■ Adults < 40 years ■ Treatment with anti-osteoporosis drugs at baseline ■ > 50% with prior diagnosis of osteoporosis, prior fragility fracture, endocrine or other disorders likely to be related to metabolic bone disease, chronic use of glucocorticoid medications, cancer
Intervention	Screening^a^ to prevent fragility fracture with any of the following: ■ Fracture risk assessment alone (validated or non-validated tools; with or without BMD incorporated, if applicable to tool) ■ Bone mineral density (BMD) alone by dual-energy x-ray absorptiometry of the femoral neck and/or lumbar spine (DXA) ± vertebral fracture assessment (VFA)/spinal radiography ■ Fracture risk assessment followed by BMD (DXA) if indicated ± vertebral fracture assessment/spinal radiography Treatment of any form is offered for those participants reaching a threshold that is either investigator-defined or based on patient and/or clinician decision making.	■ Other BMD or osteoporosis screening tests (e.g., quantitative ultrasound, quantitative computed tomography, peripheral DXA, trabecular bone score, bone turnover markers) ■ VFA without BMD
Comparator	*KQ1a*: No screening *KQ1b*: ■ Another screening strategy (e.g., 1 vs. 2 step screening) ■ Screening using a different risk assessment tool	■ Other BMD or osteoporosis-related screening tests ■ Fracture liaison services
Outcomes	*Benefits* Critical ■ Hip fractures ■ Fracture-related mortality ■ Functionality and disability (includes surrogate measures such as frailty questionnaires and long-term care admissions) ■ Quality of life or well being ■ All clinical fragility fractures^b^ Important ■ All-cause mortality *Harms* Critical ■ Serious adverse events^c^ (including all serious cardiovascular events; serious cardiac rhythm disturbances (e.g., atrial fibrillation or ventricular arrhythmia); serious gastrointestinal (GI) events (excluding cancers); GI cancer; atypical femoral fractures; osteonecrosis of the jaw) Important ■ Overdiagnosis ■ Discontinuations due to adverse events ■ Non-serious adverse events (including any adverse events or adverse (drug) reactions; any non-serious adverse events)	
Timing	Follow-up ≥ 6 months	Follow-up < 6 months
Setting	Primary health care [117]	Long-term care facilities
Study design and publication status	■ Randomized controlled trials ■ Clinical controlled trials, only if needed^d^ ■ Manuscripts, reports, abstracts, dissertations, and clinical trials registers, if data are available	■ Systematic reviews, meta-analyses, and pooled analyses ■ All other primary study designs ■ Non-research (e.g., editorials) ■ Studies available only as conference proceedings or other grey literature, unless data are available and adequate to assess study design and risk of bias
Language	English or French	All other languages
Date of publication	Any	Not applicable

^a^ Screening includes the intervention, follow-up, referral and/or treatment. Fracture risk assessment tools are considered to be any paper or electronic tool or set of questions using ≥ 2 demographic and/or clinical risk factors to assess risk of future fracture

^b^ Clinical fragility fractures include only symptomatic and radiologically confirmed fractures, sites per author definition, and may be defined as major osteoporotic fracture

^c^ A serious adverse event is any untoward medical occurrence that at any dose (a) results in death, (b) is life-threatening, (c) requires inpatient hospitalization or prolongation of existing hospitalization, (d) results in persistent or significant disability/incapacity, (e) is a congenital anomaly/birth defect, (f) is a medically important event or reaction [118]

^d^ If certainty in the evidence is a barrier to the development of recommendations, and the CTFPHC believes that further evidence from CCTs may influence their recommendations

**Table 2:** Key question 2 (accuracy of screening tests) study eligibility criteria

Criteria	Include	Exclude
Population	Asymptomatic adults ≥ 40 years in the general population (we will include studies where ≥ 80% of the sample or the sample mean age − 1 standard deviation is ≥ 40 years) *Subgroups for decision-making*: age, sex, menopausal status *Methods subgroups*: treatment with anti-osteoporosis drugs, baseline predicted fracture risk, length of follow-up	■ Adults < 40 years ■ > 50% with prior diagnosis of osteoporosis, prior fragility fracture, endocrine or other disorders likely to be related to metabolic bone disease, chronic use of glucocorticoid medications, cancer
Intervention	Screening tool to prevent fragility fracture using any of the following approaches: ■ Fracture risk assessment alone ■ Bone mineral density (BMD) alone by dual-energy x-ray absorptiometry of the femoral neck or lumbar spine (DXA) ± vertebral fracture assessment/spinal radiography ■ Fracture risk assessment followed by/incorporating BMD (DXA) ± vertebral fracture assessment/spinal radiography When a risk assessment tool is used it must have been externally validated to predict fragility fractures in a population within a very high human development index country [119] with a fracture rate similar to Canada (i.e., moderate) [57]	■ Risk assessment tools not externally validated (different population than derivation cohort) to predict fragility fractures. ■ Validation studies conducted in a population within a country that does not have a very high human development index, and/or has a different fracture rate to Canada ■ Other BMD or osteoporosis-related screening tests (e.g., quantitative ultrasound, quantitative computed tomography, peripheral DXA, trabecular bone score, bone turnover markers)
Outcomes	Calibration (total/average and by differing estimated risks, e.g., expected vs. observed fractures, goodness-of-fit, calibration slope) for 5- and 10-year fracture risk of: ■ Hip fractures ■ All clinical fragility fractures *Note*: Discrimination (e.g., sensitivity, specificity, area under the receiver operating characteristics curve/c-statistic, positive predictive value, negative predictive value) will not be included, but will be reported as it has been previously in the 2018 USPSTF systematic review as evidence to support contextual and implementation aspects of the guideline. When BMD is used alone, only discrimination outcomes will be reported since calibration is not relevant.	
Timing	Any length of follow-up; to make predictions for 5- or 10-year fracture	Not applicable
Setting	■ Primary health care [117] ■ Very high human development index country [119] with a fracture rate similar to Canada (i.e., moderate) [57]	■ Long-term care facilities ■ Countries that are not very high human development index and/or have a different fracture rate than Canada
Study design and publication status	■ Prospective or retrospective cohort studies with a defined index screen (assessed before fracture measurement); may be randomized comparisons between different index tests, but all patients assessed for fracture and each arm treated separately ■ Manuscripts, reports, abstracts, dissertations, and trial registers with data available	■ Systematic reviews, meta-analyses, and pooled analyses ■ All other primary study designs ■ Non-research (e.g., editorials) ■ Studies available only as conference proceedings or other grey literature, unless data are available and adequate to assess risk of bias
Language	English or French	All other languages
Date of publication	Any	Not applicable

*Note that studies of tools (that incorporate mortality in their risk algorithms) that do not consider death hazards in their observed fracture rate will be included but may contribute to downgrading the certainty in the evidence.

**Table 3:** Key question 3 (benefits and harms of treatment) study eligibility criteria

Criteria	Include	Exclude
Population	*KQ3a*: Adults ≥ 40 years in the general population who are at risk of (as per study authors) fragility fracture (we will include studies where ≥ 80% of the sample or the sample mean age − 1 standard deviation is ≥ 40 years) *KQ3b*: Adults ≥ 40 years who are at risk of fragility fracture *Subgroups for decision-making*: age, sex, menopausal status *Methods subgroups (KQ3a)*: prior fracture, predicted fracture risk, length of follow-up	*KQ3a:* ■ Adults < 40 years (mean age – 1 standard deviation < 40) ■ > 50% with prior fragility fracture, endocrine or other disorders likely to be related to metabolic bone disease, chronic use of glucocorticoid medications, cancer *KQ3b:* ■ Adults < 40 years ■ Endocrine or other disorders likely related to metabolic bone disease, cancer
Intervention	Pharmacotherapy currently approved by Health Canada for the treatment of osteoporosis or prevention of fragility fractures (see Additional file 3) that is commonly used in Canada as a first-line treatment: ■ Bisphosphonates (alendronate, risedronate, zoledronic acid only); harms of bisphosphonates as a class will be included if > 90% of participants are taking alendronate or risedronate, or if within-study subgroup analysis for these drugs is available ■ Denosumab Adjunct calcium and/or vitamin D (but not other drugs) will be included if it is used identically in both the intervention and comparison group	■ Pharmacotherapies not commonly used in Canada: hormone therapy, etidronate, raloxifene, teriparatide, calcitonin (no longer recommended) ■ 5 mg/day dosage of alendronate ■ Drugs used in combination ■ Off-label pharmaceuticals and dosages ■ Natural health products, dietary supplements (e.g., vitamins, minerals) ■ Complex interventions (e.g., pharmacotherapy + exercise)
Comparator	*KQ3a:* Placebo *KQ3b:* Placebo or no treatment; no comparator for the outcomes of osteonecrosis of the jaw and atypical femoral fractures In both cases adjunct calcium and/or vitamin D will be included if it is used identically in both the intervention and comparison group.	■ Other drugs, dosages, or drug combinations ■ Complex interventions (e.g., pharmacotherapy + exercise)
Outcomes	*KQ3a:* ■ Hip fractures ■ Fracture-related mortality ■ Functionality and disability (includes surrogate measures such as frailty questionnaires and long-term care admissions) ■ Quality of life or well being ■ All clinical fragility fractures^a^ ■ All-cause mortality *KQ3b:* ■ Discontinuations due to adverse events ■ Serious AEs^b^ including all serious cardiovascular events; serious cardiac rhythm disturbances (e.g., serious atrial fibrillation or ventricular arrhythmia); serious gastrointestinal events (excluding cancers); gastrointestinal cancer; atypical femoral fractures; osteonecrosis of the jaw; fractures related to rebound effects after stopping treatment ■ Non-serious adverse events (including any adverse events or adverse (drug) reactions; any non-serious adverse events)	
Timeframe	≥ 6 months follow-up	< 6 months follow-up
Setting	*KQ3a*: Primary health care [117] *KQ3b*: Primary health care or long-term care	*KQ3a*: long-term care *KQ3b*: All other settings
Study design and publication status	*KQ3a*: ■ Randomized controlled trials ■ Manuscripts, reports, abstracts, dissertations, and clinical trials registers, if data are available *KQ3b*: ■ Randomized controlled trials ■ Controlled observational studies (> 1000 participants) for serious adverse events only ■ Uncontrolled cohort studies for osteonecrosis of the jaw and atypical fractures only ■ Manuscripts, reports, abstracts, dissertations, and clinical trials registers if data are available	*KQ3a*: ■ Systematic reviews, meta-analyses, and pooled analyses ■ All other primary study designs ■ Non-research (e.g., editorials) ■ Studies available only as conference proceedings or other grey literature, unless data are available and adequate to assess risk of bias *KQ3b*: ■ Systematic reviews, meta-analyses, and pooled analyses ■ Non-research (e.g., editorials) ■ Studies available only as conference proceedings or other grey literature, unless data are available and adequate to assess risk of bias ■ Case reports and series
Language	English or French	All other languages
Date of publication	Any	Any

^a^ Clinical fragility fractures include only symptomatic and radiologically confirmed fractures; sites per author definition, and may be defined as major osteoporotic fracture.

^b^ A serious adverse event is any untoward medical occurrence that at any dose (a) results in death, (b) is life-threatening, (c) requires inpatient hospitalization or prolongation of existing hospitalization, (d) results in persistent or significant disability/incapacity, (e) is a congenital anomaly/birth defect, (f) is a medically important event or reaction [118]

**Table 4:** Key question 4 (acceptability of screening and/or treatment) study eligibility criteria

KQ4	Inclusion	Exclusion
Population	■ Adults aged ≥ 40 years (we will include studies where ≥ 80% of the sample or the sample mean age − 1 standard deviation is ≥ 40 years) ■ *Population subgroups* include: absolute fracture risk (perceived or actual; may use other risk factors as proxy: age, sex, menopausal status, BMD); prior screening, history of fracture, prior use of anti-osteoporotic medication; prior diagnosis of osteoporosis; level of concern about fractures or perceived severity of fractures	■ Adults < 40 years ■ Current use of anti-osteoporosis drugs (> 10% of population), although study participants may have recently received a prescription or recommendation to start treatment. ■ Studies with > 50% population with a prior fragility fracture, or secondary causes of osteoporosis (e.g., endocrine disorders, chronic glucocorticoid medications, etc.); unless study provides data for participants without these conditions
Exposure/intervention	■ Population may or may not have knowledge of their own medical fracture risk/BMD but must have at least some general scenario or background information (e.g., scenarios, vignettes, educational material, decision aid) containing the possible magnitude of benefits and/or harms from screening* or treatment** for fragility fractures or osteoporosis, or ■ Investigators solicit the magnitude of benefits and/or harms where screening or treatment is acceptable. *Context of screening should involve use of absolute risk assessment tools and/or dual-energy x-ray absorptiometry (DXA) with or without vertebral fracture assessment (VFA) or spinal X-ray **Treatments of interest include bisphosphonates or denosumab *Exposure subgroups*: different presentation of information (e.g., magnitudes of effects, absolute vs relative effects, number of outcomes presented)	■ Context of screening using other BMD or osteoporosis screening tests (e.g., quantitative ultrasound (QUS), quantitative computed tomography (QCT), peripheral DXA (pDXA), trabecular bone score (TBS), bone turnover markers, vertebral fracture assessment, or radiography without BMD) ■ Benefit and harm information about other treatments (e.g., hormone therapy, calcitonin, parathyroid hormone, raloxifene, exercise programs +/− other complex interventions, vitamin D, calcium, or other dietary supplements alone)
Comparator	■ None ■ Non-active exposure: Intervention without information about the possible magnitude of benefits and/or harms of screening or treatment (e.g., pamphlet on bone health or fracture risk factors) ■ Information on alternative screening (e.g., tools, intensity) or treatment (e.g., thresholds) strategy (above criteria apply)	See above for exposure
Outcomes	Acceptability measures: ■ Willingness or intentions to screen or initiate treatment ■ Acceptability of screening or initiating treatment ■ Uptake of screening or treatment ■ Absolute risk for fracture to make treatment acceptable ■ Others as suitable, as reported by authors (e.g., intent to return for another screen, magnitude of benefits to make screening and/or treatment acceptable)	
Publication date	■ 1995 (introduction of bisphosphonates)	
Study design	■ Any quantitative study design (e.g., RCT, survey), and quantitative data from mixed methods studies	■ Systematic reviews, meta-analyses, and pooled analyses ■ Non-research (e.g., editorials, commentaries, opinions) ■ Studies available only as conference proceedings or other grey literature, unless data are available and adequate to assess risk of bias ■ Qualitative studies
Setting	■ Primary health care [117]	■ Long-term care or hospital setting

Note that studies of tools (that incorporate mortality in their risk algorithms) that do not consider death hazards in their observed fracture rate will be included but may contribute to downgrading the certainty in the evidence.

### Literature search

Where possible, we will either update another systematic review or (if a single review is not a good candidate for an update) follow the Task Force’s approach to integrating studies from existing reviews [120]. For the integration approach, we will use multiple previously published systematic reviews to identify studies meeting our criteria, then run update searches to identify evidence published more recently. We will re-analyze data and re-interpret the results using Task Force methods, although we may rely on the reporting in other reviews for data extraction or, possibly, methodological quality assessments. To locate potential candidate reviews for an update, we conducted a comprehensive search for relevant systematic reviews and carefully inspected these reviews for suitability. Important considerations included the comprehensiveness of the original search (i.e., ability to capture studies of interest), the quality of reporting, and whether the eligibility criteria were similar enough to ensure that all studies of interest would be identified (or in some cases could be reliably identified from the excluded studies list or by other means). Details of the planned approach for each KQ are provided in the paragraphs that follow.

For KQ1 (benefits and harms of screening), KQ2 (accuracy of screening tests), and KQ3a (benefits of treatment), we identified the USPSTF’s 2018 systematic review [19] as suitable for updating, with some modifications. The latest search was to October 2016 with surveillance up to March 2018. We will perform a full update search from January 1, 2016, onwards to locate newly published primary studies that meet our eligibility criteria. We plan to include studies regardless of methodological quality; although the USPSTF excluded studies deemed to be of poor quality (i.e., fatally flawed), they report these in an explicit manner. The authors of this review also cite, in their excluded studies list, all the studies reporting on calibration (KQ2) that were not conducted in the United States (i.e., did not meet inclusion criteria). Due to other differences in eligibility criteria, we will also use the review’s excluded studies list and reference lists from other reviews and major guidelines, to locate clinical controlled trials and screening trials with an active comparator for KQ1b (comparative effectiveness of screening approaches). Pending quality checks (see section on Data Extraction), we plan to rely to at least some extent on the reporting of the USPSTF review for data extraction and (as one of two reviewers) risk of bias appraisals for studies included in their review.

For KQ3b (harms of treatment), we identified the Agency for Healthcare Research and Quality’s 2012 systematic review [94] (updated in 2014 for randomized controlled trials of bisphosphonates) as suitable for integration into the present review (for randomized controlled trials), along with 26 other systematic reviews that included observational studies on serious adverse events that may not have been captured in the Agency for Healthcare Research and Quality’s review (Additional file 4). Compared with the aforementioned USPSTF review, the population eligibility criteria of the Agency for Healthcare Research and Quality were more inclusive (e.g., including people with previous fragility fractures), thus more closely matching the criteria used for this KQ. The search for this review was conducted in March 2011 with a more recent update to March 2014 for (trials of) bisphosphonates [121]. We will perform a full update search from January 1, 2010, onwards to locate additional published primary studies that meet our eligibility criteria.

For KQ4, we will perform a *de novo* review and search for studies published from 1995 (date of approval of bisphosphonates) to present.

Comprehensive searches for each KQ have been developed and will be implemented by a research librarian. Searches combine Medical Subject Heading terms and key words for bone health, fracture, osteoporosis, screening, DXA and risk assessment tools (by name), the drugs of interest, and others relevant to the KQ of interest (Additional file 5 shows the search strategies). The searches were peer-reviewed by a second librarian with systematic review experience, as recommended by the Peer Review of Electronic Search Strategies guideline statement [122]. We will search Ovid Medline, Ovid Embase, and Wiley Cochrane Library; for KQ4, we will also search PsycINFO. For KQ 1 and 3, we will also search trials registries (clinicaltrials.gov, World Health Organization International Clinical Trials Registry Platform) for entries 2016 onwards. We will restrict searches to records published in English or French, based on evidence that the findings of systematic reviews on conventional medicine topics do not appear to be biased by such restrictions [123, 124]. To locate potential studies not identified by the electronic database searches, we will scan the reference lists of relevant systematic reviews (published after 2013) and the included studies found from the database searches.

We will export the results of database searches to an EndNote Library (version X7, Clarivate Analytics, Philadelphia, US) for record-keeping and to remove duplicates. We will document our supplementary search process (i.e., for any study not originating from the database searches) and enter these into EndNote individually. We will update electronic database searches for all KQs approximately 4 to 5 months prior to publication of the Task Force guideline.

### Selection of studies

Records retrieved from the database searches will be uploaded to DistillerSR (Evidence Partners Inc., Ottawa, Canada) for screening. We will screen all records retrieved via database searches in a two-step selection process, according to predefined eligibility criteria (described herein). Prior to each stage of screening, reviewers will pilot the eligibility criteria on a random sample of 50 titles/abstracts and 20 full-text studies, with further pilot rounds conducted on an as-needed basis. We will first review the titles and abstracts of all records for relevance using a liberal-accelerated approach [125, 126]. One reviewer will screen all records and classify them as “include/unsure,” “exclude,” or “reference.” Those marked as “include/unsure” by any single reviewer will move forward for full-text review, whereas those marked as “exclude” will be independently assessed by a second reviewer to confirm or refute their exclusion. One reviewer will review the “reference” category, including scanning the reference lists of the included studies and relevant systematic reviews identified by the search, and any potentially relevant citations will move forward for full-text review. Two reviewers will then independently scrutinize full-text studies for eligibility and reach consensus on their inclusion in the review. Disagreements about studies to be included will be resolved by discussion or the involvement of a third reviewer with methods or clinical expertise. If the details required for inclusion are not adequately reported in a study, we will contact first authors by electronic mail (three times over one month) to request the additional information needed to make a final decision. We will also contact the first/primary authors of relevant protocols, trial registries, abstracts, and any other reports where full study details are unavailable, to inquire about completed publications. We will document the flow of records through the selection process, with reasons provided for all full-text exclusions, and present these in a PRISMA flow diagram [116] and appended excluded studies list.

### Data extraction

We will develop a standardized form to assist in extracting relevant data. To verify that the form will accurately and completely capture the desired data, reviewers will pilot the form on a random sample of three to five included studies, with further piloting on an as-needed basis. Following a quality check of a 10% random sample, if no errors are found that would possibly change the conclusions of the review (e.g., large study where effects in intervention and control groups have been reversed), we will rely (i.e., cut and paste) on data previously extracted from the primary systematic reviews that we identified for updating or integration. Any additional data from the studies in the reviews will be extracted by one reviewer and verified by another with the exception (for KQs 1, 2, 3a) of results data which will be extracted in duplicate. For studies not included in the reviews, verification (study and population characteristics) or independent extraction (results data) will be conducted. For KQ3b (harms of treatment) where we expect over 200 studies, we will only have resources to verify accuracy of results data. If needed, we will extract estimates of data points from graphs using Plot Digitizer software [127]. For calibration outcomes, where possible, we will use guidance on reviews for prognostic models to estimate the total expected versus observed fractures (e.g., from bar graphs) for the population as a whole and across risk strata [128]. Apart from total calibration, we will report (descriptively) findings from each study on how calibration varied across differing estimated fracture risks (e.g., by deciles; low vs median vs high values).

Additional file 3 shows a detailed list of the data extraction items of interest, including how we will differentiate between count (total number of events) and dichotomous/binary (number of people experiencing one or more events) data. For randomized trials in KQ1 and KQ3b, we will prioritize outcome data derived by analyzing all individuals randomized (i.e., intention-to-treat approach). We will extract data as reported in the individual studies and not make assumptions about the lack or presence of an outcome if it is not reported. We will contact study authors (three times over one month) if important study data appear to be missing or are unclear. When there are multiple publications of the same study, we will consider the earliest full publication of the primary outcome data to be the primary data source, while all others will be considered as secondary sources/associated publications. We will extract data from the primary source first, adding in data from the secondary source(s). Throughout the report, we will reference the primary source, and cite secondary sources when applicable.

### Risk of bias assessment

For KQ1 (benefits and harms of screening), KQ2 (accuracy of screening tests), and KQ3a (benefits of treatment), we will use previous risk of bias or quality assessments reported in the 2018 USPSTF review to represent a single reviewer; another reviewer will conduct an independent assessment and develop consensus with the reported assessments. A third reviewer will be consulted as needed. The 2018 USPSTF used the Cochrane Risk of Bias Tool [129] to assess randomized controlled trials (KQ1 and KQ3a) and the Prediction model Risk Of Bias Assessment Tool [130, 131] to assess prognostic accuracy studies (KQ2).

The 2012 Agency for Healthcare Research and Quality review only assessed the risk of bias for the studies also reporting fracture outcomes (benefits) such that assessments for many randomized controlled trials (only reporting harms) were not conducted. Moreover, for the studies that were assessed, the authors applied the Jadad scale [132]. We will re-assess risk of bias for all randomized controlled included in KQ3b (harms of treatment) using a modified Cochrane risk of bias tool (see Additional file 3), because use of the Jadad scale has been discouraged due to its focus on reporting (rather than conduct), lack of assessment of bias related to allocation concealment, and overall concerns regarding the weighting of items in scales to judge risk of bias [133]. We will use the Newcastle-Ottawa Quality Assessment Scale [134] to assess (controlled) cohort and case-control studies. For surveys/cross-sectional studies (KQ4) and uncontrolled cohorts, we will use the relevant tool developed by the National Institutes of Health’s National Heart, Lung, and Blood Institute [135].

For all newly included studies for KQs 1, 2 and 3a, and 4, two reviewers will independently appraise study-level (or outcome-level, as appropriate) risk of bias or quality using the same tools. Due to the large volume of included studies expected for KQ3b (> 200), appraisals in this case will be completed by one reviewer with verification by another. Prior to beginning the appraisals, reviewers will pilot each tool’s criteria on a random sample of three to five included studies and develop decision rules to aid in their assessments. Disagreements between reviewers will be resolved by discussion or the involvement of a third reviewer, if needed. The results of our appraisals will inform the study limitations domain of our assessment of the certainty of the body of evidence. We will report all assessment results by and across studies, for each domain and using the overall assessments.

### Data synthesis

We will provide a summary of the average effect across studies using approaches relevant to the outcomes for each KQ. We will consider clinical and methodological heterogeneity in our decision to pool study data via meta-analysis. When study data are not appropriate for statistical pooling, we will describe the findings narratively and compare them to average effect estimates from corresponding meta-analyses.

#### Key questions 1 and 3

We will inspect studies for methodological and clinical heterogeneity, and if appropriate, for KQ1 (benefits and harms of screening) and KQ3 (benefits and harms of treatment), we will pool data for each outcome via pairwise meta-analysis using the DerSimonian and Laird random effects model [136] in Review Manager (version 5.3, The Cochrane Collaboration, Copenhagen, Denmark). In the case of rare events (< 1% event rate, e.g., adverse events), we will instead consider using the Peto odds ratio [137] method in order to provide a less biased effect estimate [138]. We will pool the data from randomized controlled trials and controlled clinical trials separately from observational studies. We will report risk ratios (RRs) or rate ratios between groups and corresponding 95% CIs for dichotomous or count data, respectively. When zero events are reported for at least one of the intervention groups, we will report the risk difference (RD) and 95% CI. For continuous outcomes, we will report the mean difference (MD) and 95% CI when all data are collected using the same measurement tool, or the standardized mean difference (SMD) and 95% CI when a variety of tools are used to describe a similar construct. When data for multiple time-points are available, we will choose to include data from the longest length of follow-up within the following categories: 6 to 12 months, 13 months to 5 years, 6 to 10 years, > 10 years.

If appropriate, we may pool data from studies of different bisphosphonates together, then analyze each bisphosphonate separately (i.e., as a subgroup) and compare estimates of effect for individual drugs to the class of bisphosphonates. For the clinical fracture and serious adverse event outcomes, we will preferentially analyze dichotomous data using a RR (primary outcome). If this is not reported by the authors, we will also consider analyzing count data using a rate ratio (surrogate outcome). The only instance in which we may consider combining dichotomous and count data in one analysis (assuming RR and rate ratios are very similar) is after clinical and statistical consultation confirms that events are rare enough and would be highly likely to have occurred in distinct patients and only once during follow-up.

We will calculate absolute effects for each outcome-comparison by applying the risk ratio from the meta-analysis to the median control group event rates from the included studies. If statistically significant, we will also calculate numbers needed to screen or treat.

#### Key question 2

If appropriate, for KQ2 (accuracy of screening tests), we will pool model calibration data for each identified screening method separately using the restricted maximum likelihood estimation approach and the Hartun-Knapp-Sidnick-Jonkman correction to derive 95% CIs [139, 140]. We will rescale total observed versus expected fracture event ratios and their variance (standard error (SE)) on the natural log scale prior to entering these into meta-analysis to achieve approximate normality [141–143]. We will report the observed versus expected fracture ratio and 95% CIs for calibration. When studies report calibration slope and/or calibration within categories (e.g., quintiles of risk), we will summarize the overall results narratively rather than extracting data for each category. We will consider model calibration to be “good” when the summary observed vs. expected fracture ratio is between 0.8 and 1.2 (i.e., there are 20% more or less events than are expected) [128].

Because discrimination outcomes (e.g., C-statistic/area under the receiver operating characteristics curve, sensitivity, specificity, positive and negative predictive values) were not rated as important by the Task Force, these will not be systematically reviewed by the Evidence Review Synthesis Centre. We will, however, present model discrimination information narratively and/or in tables as reported in the USPSTF review. We will consider model discrimination to be “good” when the summary C-statistic is > 0.75 (where 0.5 indicates no concordance and 1.0 indicates perfect concordance) [98].

#### Key question 4

We expect to perform a narrative synthesis given the likely heterogeneity in study designs, exposure characteristics (e.g., differences between studies in presentation of information on screening or treatment effects), populations, and outcomes reported across the studies. We will generally follow the guidance developed by Popay et al. [144] recognizing that our question of acceptability differs to some extent from questions about intervention effects or implementation factors. We will begin with a preliminary synthesis of the findings across studies and follow this with an exploration of the relationships between the studies, focusing on our population and exposure subgroups of interest (see Table 4) as well as other factors such as methodological quality. We will attempt to provide a best estimate of the acceptability of screening and/or treatment initiation (e.g., by people having information on the benefits and harms in absolute terms and with similar magnitude as thought to be applicable to the population of those at general risk for fracture), as well as factors that may impact the acceptability.

#### Dealing with missing data

If data required for meta-analysis are not directly reported by individual studies, whenever possible, we will compute or estimate these using other statistics presented in the studies, based on available guidance [128, 145]. If necessary, we will substitute means with medians. If standard deviations (SDs) or SEs are not reported, we will compute these from CIs, z- or t-statistics, or *p* values [146]. When computing SDs for change from baseline values, we will assume a correlation of 0.5 unless data pertaining to the actual correlation are available. If none of these data are available, we will approximate the SD using the range or interquartile range [147]. If it is not possible to compute or estimate the SD from other available data and the number of missing SDs is small, we will impute the mean SD from other studies in the meta-analysis, as this approach has been shown to minimally impact average effect estimates and their 95% CIs [148]. For KQ2 (accuracy of screening tests), we will estimate the log of the observed versus expected fracture ratio and its variance using available data (e.g., observed vs. expected fracture ratio, observed and expected events, observed and expected outcome probabilities, calibration-in-the-large) and standard formulae [128, 149, 150].

#### Assessment of heterogeneity

Our approach to subgroup analysis for KQs 1–3 will be to first report on within-study subgroup data for our pre-specified subgroups of interest (see Tables 1, 2, 3). Within-study findings are usually not available across all studies and can be difficult to conceptualize across a body of evidence. Thus, we will further explore heterogeneity in effects (i.e., in direction or magnitude of effects) using an exploratory between-study approach whereby we will categorize studies into subgroups; for population subgroups, we will use a large majority (e.g., ≥ 80% of participants) for classifying groups. To assess differences across subgroups, we will use appropriate statistical techniques (e.g., meta-regression if more than 8–10 studies) or stratify the meta-analysis by subgroup. We will interpret the plausibility of subgroup differences cautiously using available guidance, without relying on statistical significance [151, 152]. To assist in our interpretation of plausibility for KQ2 (accuracy of screening tests), we will calculate the 95% prediction interval as an estimate of the range of potential model performance in a new validation study and present these values along with the results of meta-analyses [128, 153].

When appropriate, we will perform sensitivity analyses (e.g., variability in overall or domain-specific risk of bias across studies, study design [randomized versus nonrandomized trials], differences in outcome definitions or adherence rates between studies) by removing certain studies from the analysis to see whether findings are different. For KQ1 and KQ3, we will perform sensitivity analyses if we have uncertainty about combining count and binary data. If substantial heterogeneity is present and cannot be plausibly explained via subgroup or sensitivity analyses, we may decide to suppress the pooled estimate of effect and instead present the findings of the comparison narratively.

#### Small study bias

When meta-analyses include at least eight studies of varying size, we will test for small study bias by visually inspecting funnel plots for asymmetry and quantitatively using Egger’s regression test (KQ1 and KQ3) [154] or the funnel inverse variance test (KQ2) [155] (significant at *P* < 0.10).

### Assessment of the certainty of effects in the body of evidence

We will not rely on previous appraisals of the certainty of the body of evidence, and instead assess this anew. Two reviewers will independently appraise the certainty of the body of evidence (i.e., “extent of our confidence that the estimates of effect are correct” [156]) for each meta-analytic comparison for the critical and important outcomes.

For KQ1 (benefits and harms of screening), KQ3 (benefits and harms of treatment), and KQ4 (acceptability of screening and/or treatment), we will assess the evidence based on five GRADE considerations: study limitations (risk of bias), inconsistency in results, imprecision of the effect estimates, indirectness of the evidence (related to our PICOTS), and publication (small study) bias [156–162]. For KQ4, we will not use publication bias, and imprecision will rely on sample sizes. We will perform separate GRADE assessments for trials and observational studies for each outcome, as applicable. For the study limitations domain, we will consider not only the studies that reported on the outcome, but also studies where it appears that the outcome should have been reported but was not (i.e., selective reporting is suspected). We will only grade the “sub-outcomes” in the serious adverse event category if there is heterogeneity in the effects between the sub-outcomes; otherwise, we will only rate the “any serious AE” outcome. Although all of evidence from KQs 2 and 3 are considered indirect for answering the primary question about screening effectiveness, we will not rate down this evidence for indirectness for this reason. We will report our assessments transparently and use a *partially contextualized approach*, whereby we assess our certainty that the true effect lies within a range of magnitudes, that might be considered “no or trivial,” “small-to-moderate,” or “moderate-to-large” [156].

In the absence of clear guidance on the applicability and interpretation of GRADE domains for prognostic studies, for KQ2 (accuracy of screening tests) calibration outcomes, we will work with experts in the field to modify existing guidance to produce an exemplar that is applicable for prognostic models.

For each outcome, we will create separate GRADE summary of findings tables [163, 164] using GRADEpro GDT software (Evidence Prime, Hamilton, ON) [165]. We will use footnotes to explain all decisions where the evidence was rated down or upwards, and comment (if applicable) on differences between the findings for trials and observational studies. The certainty assessments for each outcome will be incorporated into the Task Force’s evidence-to-decision framework [166]. The Task Force may alter the appraisals when *fully contextualizing* the assessment while considering the findings across outcomes (e.g., on benefits and harms) [156]. They will then will use this information to assess the net benefits and harms of screening, and then consider other elements of the GRADE methodology (i.e., feasibility, patient values and preferences, effect magnitude, resource implications such as the cost of screening and interventions) to develop recommendations on screening to prevent fragility fracture [166].

## Discussion

The 2010 Osteoporosis Canada Guidelines are the most recent available national recommendations for screening to prevent fragility fracture in Canada. Since publication of the guidelines, new trial evidence has become available that may alter recommendations [4, 5]. We will undertake an updated systematic review of the available research relevant to screening for fragility fracture. We anticipate some challenges in updating previous systematic reviews, due to some differences in eligibility criteria and variable reporting in the eligible reviews. We have incorporated methods to overcome these challenges (e.g., scanning excluded studies lists or other systematic reviews). The Task Force will use the results of this systematic review to develop evidence-based recommendations for screening of adults ≥ 40 years for fragility fracture in primary care.

## Additional files

Additional file 1: Summary of available screening guidelines. This file documents a variety of screening guidelines for fragility fracture. (DOCX 31 kb)

Additional file 2: Completed PRISMA-P checklist. This file documents the protocol’s adherence to PRISMA-P. (DOCX 29 kb)

Additional file 3: Supplementary information on selection criteria, data extraction items, and risk of bias assessment. This file contains detailed information about the selection criteria, data extraction items, and risk of bias assessment. (DOCX 36 kb)

Additional file 4: Identified systematic reviews with adverse events data from observational studies for KQ3b. This file contains a list of systematic reviews identified for integration in KQ3b. (DOCX 18 kb)

Additional file 5: Search strategies. This file contains the planned search strategies for the review. (DOCX 46 kb)

## Data Availability

Not applicable

## Abbreviations

AE: Adverse event
BMD: Bone mineral density
CAROC: Canadian Association of Radiologists and Osteoporosis Canada fracture risk assessment tool
CI: Confidence interval
DXA: Dual-energy x-ray absorptiometry
FRAX: Fracture Risk Assessment tool
GRADE: Grading of Recommendations Assessment, Development and Evaluation
KQ: Key Question
MD: Mean Difference
PICOTS: Population, Intervention, Comparator, Outcome, Timeline, Setting/Study design
PROSPERO: International Prospective Registry of Systematic Reviews
RD: Risk difference
RR: Risk ratio
SD: Standard deviation
SE: Standard error
SMD: Standardized mean difference
US: United States
USPSTF: United States Preventive Services Task Force
