# Supplementary material for: Screening to prevent fragility fractures among adults 40 years and older in primary care: protocol for a systematic review
Source: Syst Rev. 2019 Aug 23;8:216. doi: 10.1186/s13643-019-1094-5 (PMC6706906; doi:10.1186/s13643-019-1094-5)
Supplement: Supplementary file 1 — Summary of available screening guidelines. This file documents a variety of screening guidelines for fragility fracture. (DOCX 31 kb) [file 13643_2019_1094_MOESM1_ESM.docx]

**Additional file 1.** Existing relevant guidelines and recommendations for screening to prevent fragility fracture or osteoporosis

| **Organization, Year** | **Target population** | **Recommendations provided** |
| --- | --- | --- |
| **International** | | |
| World Health Organization, 2007 [1, 2] | Adults | - The report directs attention away from the sole use of BMD to determine treatment thresholds, and instead recommends the assessment of absolute fracture risk (either by BMD testing or other validated instruments). - The use of clinical risk factors together with BMD will allow for the effective and efficient delivery of healthcare to high risk individuals while avoiding unnecessary treatment of others. - In Member States where densitometry is unavailable, case-finding strategies can include clinical risk factors alone. - In Member States where BMD is universally recommended, stratification of risk can be improved by the consideration of clinical risk factors. - In Member States with limited access to DXA, clinical risk factors can be used to stratify risk groups and to determine those where a BMD test would help to characterize their fracture probability. |
| Endocrine Society, 2012 [3] | Men | - Men aged ≥70 years and younger men (50 to 69 years) with risk factors should have BMD measured. Risk assessment tools such as FRAX, Garvan, and others can improve the assessment of fracture risk. - DXA of the spine and hip is recommended for men at risk for osteoporosis. Forearm DXA can be used with spine or hip BMD cannot be interpreted or is not appropriate. - A complete history and physical exam is recommended for men being evaluated for osteoporosis or being considered for pharmacological treatment. Several biochemical tests are recommended. Further testing might be required if specific causes of osteoporosis are suspected. - In men with osteopenia or osteoporosis who might have undiagnosed vertebral fractures, vertebral fracture assessment via DXA is recommended. If this is not possible, lateral spine radiographs may be considered. |
| International Society for Clinical Densitometry, 2015 [4] | Adults | - BMD testing is indicated for all women ≥65 years; post-menopausal women <65 years and those in the menopausal transition who have risk factors; men ≥70 years; men <70 years with risk factors; adults with a fragility fracture; adults with a disease, condition, or who are taking medications associated with low bone mass or bone loss; anyone being considered for treatment, being treated, or for whom evidence of bone loss could lead to treatment. - Central DXA (spine and hip) is recommended for diagnosis based on WHO criteria; forearm may be used in special circumstances. - For fracture risk assessment, any well-validated technique may be used. |
| **North America** | | |
| North American Menopause Society, 2010 [5] | Postmenopausal women | - Physical examination should include height and weight measurement, assessment of chronic back pain, kyphosis, and clinical risk factors. - BMD testing is indicated for all women ≥65 years and postmenopausal women with medical causes of bone loss, and should be considered for postmenopausal women ≥50 years with one or more risk factors. - DXA is the preferred method of BMD testing, and the lowest BMD score of 3 sites (total hip, femoral neck, posterior anterior lumbar spine) should be used. |
| **Canada** | | |
| Osteoporosis Canada, 2010 [6, 7] | Adults | - Individuals >50 years who have experienced a fragility fracture should be assessed for established risk factors. - Height should be measured annually to assess for presence of vertebral fractures, and history of falls in the past year should be assessed. - Indications for measuring BMD include individuals ≥65 years; menopausal women; men aged 50-64 years with clinical risk factors; and younger adults <50 years who are affected by medical conditions that impact bone metabolism or fracture risk. - 10-year major osteoporotic fracture risk should be estimated in those >50 years using CAROC or FRAX, incorporating BMD T-score at the femoral neck. For those <50 years, consultation with a specialist is recommended. |
| Canadian Association of Radiologists, 2013 [8] | Adults | - Absolute fracture risk category should be reported for adults ≥50 years when a relevant history is available. - In adults ≥50 years, the CAROC should be used to estimate fracture risk, incorporating BMD. For those <50 years, absolute risk assessment is not available and should not be reported. - The timing of follow-up assessments (or screening) should be noted, and will depend on the expected rate of BMD change over time |
| Menopause and Osteoporosis Working Group, 2014 [9] | Postmenopausal women | - For postmenopausal women, healthcare providers should estimate absolute fracture risk by integrating key risk factors using CAROC or FRAX; treatment should be initiated based on the results of the 10-year fracture risk assessment. - The presence of a previous fragility fracture confirms a diagnosis of osteoporosis regardless of BMD. - The presence of a hip or vertebral fragility fracture, or more than one fragility fracture at any site, confirms high fracture risk regardless of BMD. |
| Toward Optimized Practice (Alberta), 2016 [10] | Adults ≥50 years | - Recommendations are based on the Osteoporosis Canada guidelines.[6, 7] - For adults 50-64 years, use the Osteoporosis Self-assessment Tool (OST) to assess risk; order a BMD test only if OST score is <10; reassess OST in 5 years if OST score is ≥10. - For adults ≥50 years with known risk factors, order BMD testing via DXA; discuss the limited value of BMD testing; suggest a BMD test for all women ≥65 years and men ≥65 years with at least one risk factor if they have not previously been tested. - Use clinical risk factors and the BMD T-score to determine 10-year fracture risk using CAROC or FRAX; consider spine radiography only if clinical evidence suggests a vertebral fracture. - Consider biochemical tests if there is a suspicion of secondary causes of osteoporosis. |
| British Columbia Medical Association and British Columbia Ministry of Health, 2012 [11] | Adults | - Two aspects of risk can be explored by identifying known risk factors: risk of developing osteoporosis, risk of fracture within 10 years. - 10-year fragility fracture risk can be estimated using FRAX or CAROC; fall risk can also be considered. Patients can be stratified by risk. - Risk stratification using BMD is not indicated unless patients are >65 years, at moderate (10-20%) 10-year fracture risk, and results are likely to alter care. - Laboratory testing is only indicated in specific circumstances. |
| **United States** | | |
| American Association of Clinical Endocrinologists and American College of Endocrinology, 2016 [12] | Postmenopausal women | - All postmenopausal women aged ≥50 years should be evaluated for osteoporosis risk. - The initial evaluation for osteoporosis should include a detailed history, physical exam, and clinical fracture risk assessment using FRAX. - BMD testing via DXA can be considered based on clinical fracture risk profile. - Osteoporosis may be diagnosed based on (a) the presence of fragility fractures without other metabolic bone disorders, (b) a T-score of -2.5 or lower in the lumbar spine, femoral neck, total hip, or 33% radius even without a prevalent fracture, (c) osteopenia accompanied by increased fracture risk using FRAX country-specific thresholds, (d) osteopenia accompanied by a fragility fracture at the proximal humerus, pelvis, or distal forearm. |
| American College of Obstetricians and Gynecologists, 2014 (reaffirmed in 2016) [13] | Women | - BMD measurement via DXA of the lumbar spine and hip is the preferred method for diagnosing osteoporosis, using WHO criteria. A history of vertebral fracture (confirmed by lateral spine imaging) or low-trauma fracture can be used to establish a diagnosis of osteoporosis. - For women, BMD measurement via DXA should begin at 65 years; DXA screening can also be used in women <65 years if they are postmenopausal or have other risk factors (can be identified using the FRAX tool). - Routine screening of newly menopausal women or ‘baseline’ screening is not recommended. |
| American College of Physicians, 2018 [14] | Men | - Clinicians should periodically perform individualized assessment of risk factors for osteoporosis in older men. - Clinicians should obtain DXA for men at increased risk for osteoporosis who are candidates for treatment. - Further research is recommended to evaluate osteoporosis screening tests in men. |
| American College of Preventive Medicine, 2009 [15] | Adults | - All adults ≥50 years should be evaluated for risk factors for osteoporosis. - Screening with BMD testing (by DXA if available) is recommended for women ≥65 years and men ≥70 years; younger postmenopausal women and men aged 50-69 years with at least one major or two minor risk factors. - Clinicians should consider a fracture risk assessment tool (e.g., FRAX) to evaluate absolute fracture risk and determine appropriate treatment. - Re-screening should not occur more frequently than every 2 years. |
| American College of Radiology, 2016 [16] | Adults | - Provides ratings of the appropriateness of various types of imaging in specific clinical scenarios. - BMD measurement via DXA is the primary choice for screening women >65 years and men >70 years for osteoporosis. - DXA is indicated for postmenopausal women <65 years with additional risk factors. - Vertebral fracture assessment can be a useful screening strategy to identify at-risk individuals whose BMD is above treatment thresholds. |
| American College of Rheumatologists [17] | Children and adults on long-term glucocorticoid treatment | - In all adults and children, an initial clinical fracture risk assessment should be performed as soon as possible (at least within 6 months of initiation of treatment); for adults ≥40 years, the initial absolute fracture risk should be estimated using FRAX. - For adults <40 years, BMD testing should occur as soon as possible if the patient is at high fracture risk (previous fracture or other risk factors). - In all adults and children who continue glucocorticoid treatment, a clinical fracture risk reassessment should be performed every 12 months; for adults ≥40 years not on anti-osteoporosis treatment, retesting should occur every 1-3 years. - Specific reassessment recommendations are also available for other groups (i.e., age groups, with and without treatment). |
| Institute for Clinical Systems Improvement, 2013 [18] | Adults | - BMD testing is recommended for women ≥65 years; adults >50 years with a recent fragility fracture; adults on chronic glucocorticoid therapy. - Shared decision making about BMD tested is recommended for men ≥70 years; adults with a known condition associated with low bone mass/bone loss; organ transplant patients. - Consider BMD assessment and further risk assessment for women <65 years and men 50-69 years; individuals with significant height loss. - For patients who are not in a high risk category, an assessment of risk factors should take place to determine if BMD testing is warranted. This may occur using a thorough history or risk assessment tool (FRAX). |
| National Osteoporosis Foundation, 2014 [19] | Postmenopausal women and men ≥50 years | - All postmenopausal women and men ≥50 years should be evaluated for osteoporosis risk in order to determine the need for BMD testing and/or vertebral imaging. Height should be measured annually. The FRAX tool may be used to assess an individual patient’s risk. - BMD measurement via DXA should be performed in all women ≥65 years and men ≥70 years; postmenopausal women and men aged 50-69 based on risk factor profile; postmenopausal women and men ≥50 years with a previous fracture. - Vertebral imaging should be performed in all women ≥70 years and men ≥80 years if BMD T-score is -1.0 or less at the spine, total hip, or femoral neck; women 65-69 years and men 70-79 years if BMD T-score is -1.5 or less; postmenopausal women and men ≥50 years with specific risk factors. - The interval between repeat BMD screenings is dependent on baseline BMD and risk factors. |
| United States Preventive Services Task Force, 2018 [20, 21] | Adults | - Women ≥65 years and menopausal women <65 years who are at increased risk of osteoporosis, as determined by a risk assessment tool, should be screened using BMD testing. - Evidence was insufficient to provide screening recommendations for men. If the service is offered, patients should understand that there is uncertainty about the benefits and risks. |
| **Europe** | | |
| European Society for Clinical and Economic Aspects of Osteoporosis and Osteoarthritis, and the International Osteoporosis Foundation, 2013 [22] | Adults | - A series of systematic reviews were conducted to act as a platform upon which national guidelines could be developed. - At present there is no universally accepted policy for population screening to identify those at high risk of fracture; most countries adopt a case finding strategy whereby those with risk factors are identified for further assessment. Potential screening scenarios depend on access to densitometry. - Unrestricted access to densitometry: fracture risk should be assessed in postmenopausal women with one or more risk factors where assessment would influence management; women with a prior fragility fracture can be considered for treatment without further assessment; for women without prior fracture, 10-year probabilities based on FRAX without BMD should be determined and if they exceed the assessment threshold these women can be considered for BMD testing via DXA. - Limited access to densitometry: as above (unrestricted access) except for women without prior fracture, those with 10-year FRAX probabilities above the threshold may be considered for treatment without BMD measurement. Those between the lower and upper thresholds can be considered for BMD testing via DXA. - No access to BMD: as above (limited access) except without further assessment of those between the lower and upper 10-year FRAX probability thresholds. - Other assessment models that differ from the algorithm above exist in some countries. |
| European Society of Endocrinology [23] | Postmenopausal women | - All postmenopausal women should receive lifestyle and nutritional optimization for bone health, and their 10-year fracture risk should be determined using country-specific guidelines. - Women at low risk should be re-assessed for fracture risk every 2-4 years. - Women at moderate risk may be considered for treatment with bisphosphonates. - Postmenopausal women at high risk of fractures, and especially those with a recent fracture, should be treated with pharmacologic therapies. Patient values and preferences should be taken into account when deciding who to treat. - Intervals for reassessment of fracture risk vary by type of treatment and level of baseline risk. |
| **United Kingdom** | | |
| National Institute for Health and Care Excellence, 2012 [24] | Adults | - Consider assessment of fracture risk for all women ≥65 years and all men ≥75 years, and younger men and women in the presence of risk factors. - Do not routinely assess fracture risk in individuals <50 years unless they have major risk factors. - Absolute fracture risk should be assessed using FRAX (without BMD measurement if it has not previously been assessed) or QFracture. Do not routinely measure BMD to assess fracture risk without prior assessment using FRAX (without BMD value) or QFracture. - Consider those above the upper age limit of these tools to be at high risk. Interpret the estimated absolute fracture risk in those >80 years with caution. |
| UK National Osteoporosis Guideline Group, 2017 [25] | Postmenopausal women and men ≥50 years | - A case finding strategy should be used whereby at-risk individuals are identified by the presence of a fragility fracture or clinical risk factors. - Fracture risk should be assessed in postmenopausal women and men ≥50 years with risk factors, using FRAX. Vertebral fracture assessment is recommended for those with a history of ≥4 cm height loss, kyphosis, glucocorticoid therapy, or BMD T-score ≤-2.5. - Those at intermediate risk should have BMD measured via DXA. - The intervention threshold for those ≤70 years is set at a level of risk that is equivalent to the presence of a prior fragility fracture (i.e., increases with age); fixed thresholds are applied for those >70 years. - Women with a prior fragility fracture should be for treatment without the need for further assessment (BMD measurement might be appropriate). |
| UK National Screening Committee, 2012 [26] | Postmenopausal women | - The committee reviewed other guidelines and available evidence and indicated that it is not appropriate to implement a national screening program for osteoporosis (inadequate evidence of cost-effectiveness; lack of consensus about who should be eligible for treatment; long term clinical and cost effectiveness is unknown). - The committee concluded that the impact of a national screening program on the population burden of fractures would be limited. |
| Scottish Intercollegiate Guidelines Network, 2015 [27] | Adults | - Individuals >50 years with a history of fragility fracture should be screened using BMD measurement via DXA to evaluate the need for treatment. - In individuals with clinical risk factors for osteoporosis and those for whom anti-osteoporosis treatment is being considered, fracture risk assessment should be undertaken prior to DXA, preferably using the QFracture tool. - When anti-osteoporosis treatment is being considered, fracture risk assessment should be followed by BMD measurement via DXA at the spine and hip. |
| **Other** | | |
| Multidisciplinary Osteoporotic Forum (Poland) [28] | Postmenopausal women and men ≥50 years | - Diagnosis of osteoporosis in postmenopausal women >50 years should be based on evaluation of BMD, 10-year absolute fracture risk, and previous osteoporotic fractures. - 10-year fracture risk should be estimated using BMI, clinical risk factors, and BMD at centrally measured sites (when available), and other independent risk factors. - Routine management should begin with screening based on FRAX. - Any existing verified fracture or serious fracture risk (>10%) is an indication for drug therapy. |
| Osteoporosis New Zealand, 2017 [29] | Adults | - Individuals who have sustained a fragility fracture, women aged <65 years with risk factors, women ≥65 years and men ≥75 years, and all users of glucocorticoids for >6 months should have BMD assessed via DXA at 2 sites (preferably anteroposterior spine and hip). - Individuals ≥75 years with a radiologically confirmed significant osteoporotic fracture do not necessarily require BMD measurement prior to treatment. - Fracture risk assessment and treatment should not be withheld if DXA is unavailable; in this case it is acceptable to assess fracture risk with FRAX or Garvan calculators without incorporating BMD. - Absolute fracture risk assessment (using FRAX or Garvan tools) should be used as the basis for therapeutic decision-making. - Timing of reassessments will depend on the expected rate of BMD change over time. |
| Osteoporosis Society of Hong Kong, 2013 [30] | Adults | - A diagnosis of osteoporosis relies on BMD measurement prior to development of a fragility fracture. - The current gold standard for diagnosing osteoporosis is DXA with classification via WHO standards. Central DXA at the hip and spine are the recommended sites. - Indications for BMD testing are based on International Society for Clinical Densitometry Recommendations.[4] |
| Royal Australian College of General Practitioners and Osteoporosis Australia, 2017 [31] | Adults ≥50 years | - All individuals aged >50 years with a fragility fracture at the hip or spine is presumptive of osteoporosis (treatment can be initiated without BMD testing). Use BMD testing via DXA at the spine and proximal femur should be used to guide management for other fracture sites. - All individuals ≥70 years, postmenopausal women, and men >50 years with risk factors should be assessed for osteoporosis. - Diagnostic investigations should include BMD measurement via DXA (spine and proximal femur), medical history and clinical examination; additional assessment of absolute fracture risk using the Garvan Fracture Risk Calculator or FRAX may be useful for those not meeting treatment criteria. - Fracture risk should regularly be re-assessed. |
| Saudi Osteoporosis Society, 2015 [32] | Adults | - In the absence of good cost-effectiveness evidence, an individualized approach to screening is recommended. Individual decisions should be informed by the evidence that fracture risk increases with age and with an increased number of risk factors. - In the absence of local data, the US version of FRAX may be used until such data become available. - Diagnosis of osteoporosis should be based on BMD measurement via DXA (following AACE guidelines for who should be measured). |
| Spanish Menopause Society, 2013 [33] | Adults | - Diagnosis and risk stratification for postmenopausal osteoporosis are based on the detection of risk factors and BMD measurement via DXA. - Measurement of BMD is recommended for postmenopausal women ≥65 years, postmenopausal women <65 years with additional risk factors, and premenopausal women with a fragility fracture. |

AACE: American Association of Clinical Endocrinologists; BMD: bone mineral density; CAROC: Canadian Association of Radiologists and Osteoporosis Canada tool; DXA: dual-energy x-ray absorptiometry; FRAX: Fracture Risk Assessment Tool; OST: Osteoporosis Self-assessment Tool; UK: United Kingdom; US: United States; WHO: World Health Organization

**REFERENCES**

1. Kanis JA, on behalf of the World Health Organization Scientific Group. Assessment of osteoporosis at the primary health-care level. Technical Report. World Health Organization Collaborating Centre for Metabolic Bone Diseases, University of Sheffield, UK; 2007.
2. World Health Organization. Assessment of osteoporosis at the primary health care level. Summary Report of a WHO Scientific Group. Geneva, Switzerland: World Health Organization; 2007.
3. Watts NB, Adler RA, Bilezikian JP, Drake MT, Eastell R, Orwoll ES, et al. Osteoporosis in men: an Endocrine Society clinical practice guideline. J Clin Endocrinol Metab. 2012;97:1802-22.
4. The International Society for Clinical Densitometry. ISCD official position: Adults. 2015 <https://www.iscd.org/official-positions/2015-iscd-official-positions-adult/>. Accessed 31 Jan 2019.
5. North American Menopause Society. Management of osteoporosis in postmenopausal women: 2006 position statement of The North American Menopause Society. Menopause 2006;13:340.
6. Papaioannou A, Morin S, Cheung AM, Atkinson S, Brown JP, Feldman S, et al. 2010 clinical practice guidelines for the diagnosis and management of osteoporosis in Canada: summary. CMAJ 2010;182:1864-73.
7. Papaioannou A, Morin SN, Cheung AM, Atkinson S, Brown JP, Feldman S, et al. Clinical practice guidelines for the diagnosis and management of osteoporosis in Canada: Background and technical report. 2010. <https://osteoporosis.ca/health-care-professionals/clinical-practice-guidelines/osteoporosis-guidelines/>. Accessed 31 Jan 2019.
8. Siminoski K, O'Keeffe M, Brown JP, Burrell S, Coupland D, Dumont M, et al. Canadian Association of Radiologists technical standards for bone mineral densitometry reporting. Can Assoc Radiol J 2013;64:281-94.
9. Khan A, Fortier M, Menopause and Osteoporosis Working Group. Osteoporosis in menopause. JOGC 2014;36:839-40.
10. Toward Optimized Practice. Diagnosis and management of osteoporosis: Clinical practice guideline. 2016. <http://www.topalbertadoctors.org/cpgs/?sid=18&cpg_cats=81>. Accessed 31 Jan 2019.
11. British Columbia Medical Association, British Columbia Ministry of Health. Osteoporosis: diagnosis, treatment, and fracture prevention. 2012. <https://www2.gov.bc.ca/gov/content/health/practitioner-professional-resources/bc-guidelines/osteoporosis>. Accessed 31 Jan 2019.
12. Camacho PM, Petak SM, Binkley N, Clarke BL, Harris ST, Hurley DL, et al. American Association of Clinical Endocrinologists and American College of Endocrinology clinical practice guidelines for the diagnosis and treatment of postmenopausal osteoporosis—2016. Endocr Pract 2016;22 Suppl 4:1-42.
13. The American College of Obstetricians and Gynecologists. ACOG practice bulletin N. 129. Osteoporosis. Obstet Gynecol 2012;120:718-34.
14. Qaseem A, Snow V, Shekelle P, Hopkins R, Forciea MA, Owens DK. Screening for osteoporosis in men: A clinical practice guideline from the American College of Physicians. Ann Intern Med 2008;148:680-4.
15. Lim LS, Hoeksema LJ, Sherin K. Screening for osteoporosis in the adult U.S. population: ACPM position statement on preventive practice. Am J Prev Med 2009;36:366-75.
16. Ward RJ, Roberts CC, Bencardino JT, Arnold E, Baccei SJ, Cassidy RC, et al. ACR Appropriateness Criteria® osteoporosis and bone mineral density. J Am Coll Radiol 2017;14:S189-S202.
17. Buckley L, Guyatt G, Fink HA, Cannon M, Grossman J, Hansen KE, et al. 2017 American College of Rheumatology guideline for the prevention and treatment of glucocorticoid‐induced osteoporosis. Arthritis Rheumatol. 2017;69:1521-37.
18. Beithon J, Gallenberg M, Johnson K, Kildahl P, Krenik J, Liebow M, et al. Institute for Clinical Systems Improvement: Diagnosis and treatment of osteoporosis. 2017. <https://www.icsi.org/guidelines__more/catalog_guidelines_and_more/catalog_guidelines/catalog_womens_health_guidelines/osteoporosis/>. Accessed 31 Jan 2019.
19. Cosman F, de Beur SJ, LeBoff MS, Lewiecki EM, Tanner B, Randall S, et al. Clinician's guide to prevention and treatment of osteoporosis. Osteoporos Int 2014;25:2359-81.
20. United States Preventive Services Task Force. Screening for osteoporosis to prevent fractures: US Preventive Services Task Force recommendation statement. JAMA 2018;319:2521-31.
21. Viswanathan M, Reddy S, Berkman N, et al. Screening to prevent osteoporotic fractures: Updated evidence report and systematic review for the US Preventive services Task Force. JAMA 2018;319:2532-51.
22. Kanis JA, McCloskey EV, Johansson H, Cooper C, Rizzoli R, Reginster JY. European guidance for the diagnosis and management of osteoporosis in postmenopausal women. Osteoporos Int 2013;24:23-57.
23. Eastell R, Rosen CJ, Black DM, Cheung AM, Murad MH, Shoback D. Pharmacological management of osteoporosis in postmenopausal women: An Endocrine Society clinical practice guideline. J Clin Endocrinol Metab 2019;104:1595-1622.
24. National Clinical Guideline Centre. Osteoporosis: Assessing the risk of fragility fracture. London, UK: National Institute for Health and Clinical Excellence; 2012.
25. Compston J, Cooper A, Cooper C, Gittoes N, Gregson C, Harvey N, et al. UK clinical guideline for the prevention and treatment of osteoporosis. Arch Osteoporos 2017;12(1):43.
26. Peto L, Allaby L. Screening for Osteoporosis in Postmenopausal Women − A Report for the UK National Screening Committee. Oxford, UK: Solutions for Public Health; 2013.
27. Scottish Intercollegiate Guidelines Network (SIGN). Management of osteoporosis and the prevention of fragility fractures (SIGN publication no. 142). Edinburgh, UK: SIGN; 2015.
28. Gluszko P, Lorenc RS, Karczmarewicz E, Misiorowski W, Jaworski M. Polish guidelines for the diagnosis and management of osteoporosis: A review of 2013 update. Polskie Archiwum Medycyny Wewnetrznej 2014;124:255-63.
29. Osteoporosis New Zealand. Guidance on the diagnosis and management of osteoporosis in New Zealand. 2017. <https://osteoporosis.org.nz/clinical-guidance/>. Accessed 31 Jan 2019.
30. Ip TP, Cheung SK, Cheung TC, Choi TC, Chow SL, Ho YY, et al. The Osteoporosis Society of Hong Kong (OSHK): 2013 OSHK guideline for clinical management of postmenopausal osteoporosis in Hong Kong. Hong Kong Med J 2013;19 Suppl 2:1-40.
31. The Royal Australian College of General Practitioners, Osteoporosis Australia. Osteoporosis prevention, diagnosis and management in postmenopausal women and men over 50 years of age, 2nd edition. East Melbourne, Vic: Royal Australian College of General Practitioners; 2017.
32. Al-Saleh Y, Sulimani R, Sabico S, Raef H, Fouda M, Alshahrani F, et al. 2015 Guidelines for osteoporosis in Saudi Arabia: Recommendations from the Saudi Osteoporosis Society. Ann Saudi Med 2015;35:1-12.
33. Mendoza N, Sanchez-Borrego R, Villero J, Baro F, Calaf J, Cancelo MJ, et al. 2013 Up-date of the consensus statement of the Spanish Menopause Society on postmenopausal osteoporosis. Maturitas 2013;76:99-107.
