# Supplementary material for: Screening to prevent fragility fractures among adults 40 years and older in primary care: protocol for a systematic review
Source: Syst Rev. 2019 Aug 23;8:216. doi: 10.1186/s13643-019-1094-5 (PMC6706906; doi:10.1186/s13643-019-1094-5)
Supplement: Supplementary file 3 — Supplementary information on selection criteria, data extraction items, and risk of bias assessment. This file contains detailed information about the selection criteria, data extraction items, and risk of bias assessment. (DOCX 36 kb) [file 13643_2019_1094_MOESM3_ESM.docx]

**Additional file 3.** Supplementary information on selection criteria, data extraction items, and risk of bias assessment

**A.** **Detailed description of the selection criteria for the review**

Population

For KQ1 (benefits and harms of screening), KQ2 (accuracy of screening tests), KQ3a (benefits of treatment), and KQ4 (acceptability) we will include studies of a general population of adults ≥40 years who are seen within primary care. The 40-year age cut-off was chosen taking into account the increasing risk of fracture with advancing age,[1] and to ensure that women in early menopause (e.g., 40 to 45 years) would be captured. To ensure that no relevant data are missed we will include studies where ≥80% of the sample, or the sample mean age -1 standard deviation, meets the criteria of being ≥40 years. For KQ3 (treatment benefits and harms), patients must be considered by the study authors to be at risk for fragility fracture. For KQ1, we will exclude studies that include patients who at baseline were being treated with anti-osteoporotic drugs. Since the population of interest is asymptomatic individuals who are not undergoing treatment for osteoporosis or are already at known increased risk for fractures, for KQs 1 and 2, we will exclude studies where a majority (>50%) of adults have been previously diagnosed with osteoporosis (criteria that align with the 2018 USPSTF review). For KQ4 (acceptability) we will exclude patients who are currently using anti-osteoporosis drugs (>10% of population), though study participants may have recently received a prescription or recommendation to start treatment. For KQs 1, 2, 3a, and 4 we will exclude studies where a majority (>50%) of adults: are known to have experienced a previous fragility fracture, have an endocrine or other disorder likely to be related to metabolic bone disease, have active cancer, or have chronic use of glucocorticoid medications. Population subgroups on which the Task Force envisions making recommendations for these KQs will include age, sex, and menopausal status. Although the Task Force does not plan to make any recommendations for people with a prior fracture or diabetes, these are important subgroups that we are referring to as “methodological subgroups” (potentially mediating or confounding effects). Diabetes is an important and easily identifiable risk factor not accounted for in many risk prediction tools. For KQ1 and 3a, we will also examine methodological subgroups based on differences in baseline predicted fracture risk; we may use post-hoc analyses of trials to inform this subgroup. For KQ2 (accuracy of screening tests) an additional methodological subgroup will be whether patients received treatment with anti-osteoporosis drugs during follow-up for the predicted outcomes. For KQ4 (acceptability), additional subgroups of interest include prior screening, fracture, or diagnosis of osteoporosis, and level of concern or perceived severity of fractures. We will use these variables when assessing the methodological quality of individual studies as well as any heterogeneity in effects between studies (see section on Data Synthesis).

Compared with the eligibility criteria proposed for other KQs, the population of interest for KQ3b (harms of treatment) will be wider because of the assumption that the incidence of harms does not differ greatly between patient populations undergoing usual recommended doses of treatment (e.g., a previous fracture will not alter presence of a serious adverse event from standard treatments). We will include all studies of adults ≥40 years, with or without prior fracture, who are treated because of possible fracture risk and may have a diagnosis of osteoporosis. We will exclude studies with a majority of patients with endocrine or other disorders likely related to metabolic bone disease, and cancer patients. Population subgroups include age, sex, and menopausal status. Our methodological subgroups will be prior fracture and diabetes.

Interventions

For KQ1 (benefits and harms of screening) and KQ2 (accuracy of screening tests) the main intervention/exposure of interest is any one- or two-step screening strategy aimed at the prevention of fragility fractures. For KQ1 we will include DXA and/or any validated or non-validated fracture risk assessment/prediction tool (i.e., any paper or electronic tool or set of questions using ≥2 demographic and/or clinical factors) used to assess risk for future fracture. We consider a strategy to be one-step if providers are interpreting one or more tools (e.g., DXA or clinical risk assessment with DXA) at the same time, while in a two-step approach the use of a second tool is based on the results of the first (e.g., risk assessment followed by BMD in a subset of patients where it is indicated). Studies included for KQ1 will include the intention to provide treatment for those assessed at a level of risk that meets a certain threshold, either investigator-defined or based on patient and/or clinician decision-making. For KQ2, the risk prediction tools need to have been externally validated (aside from the initial development population) within a population in a very-high human development index country[2] with a similar hip fracture incidence to Canada (moderate).[3] BMD as a tool on its own will be included for KQ2, but only for discrimination outcomes (i.e., ability of the test to distinguish between those who will or will not fracture, measured via area under the receiver operating characteristics curve and other measures of accuracy [e.g., sensitivity, specificity], relying on a particular threshold) since calibration (i.e., accuracy of the absolute risk prediction), the critical outcome of interest related to test accuracy, does not apply to BMD which does not on its own provide an estimate of absolute fracture risk. For the ascertainment of BMD, we will include DXA of the femoral neck (main site) and/or lumbar spine. We will exclude all other, less common measures of BMD (e.g. quantitative ultrasound, quantitative computed tomography, peripheral DXA, trabecular bone score, bone turnover markers), as they typically cannot be incorporated into risk assessment tools and have not been shown to improve fracture risk prediction.[4-6] Vertebral fracture assessment and/or spinal radiography may be included if it is performed in addition to DXA, but it is not within the scope of this review to consider studies where either of these tests are used alone.

For KQ3 (benefits and harms of treatment) the intervention of interest is any commonly used first-line pharmacotherapy that is currently approved by Health Canada for the prevention of fragility fractures or treatment of osteoporosis (i.e., alendronate, risedronate, zoledronic acid, denosumab) (Additional table 1, below).[7] Though it is registered as a biologic, we will consider denosumab as a pharmaceutical for the purposes of this review. We will exclude etidronate and teriparatide, which are not first-line treatments. Though Osteoporosis Canada recommends raloxifene as a first-line therapy for post-menopausal women,[8] we will exclude it because it is not widely used in Canada and is not recommended as first line in recent treatment guidelines.[9, 10] Hormone therapy is also excluded because it is only recommended for women with vasomotor symptoms,[8] and recommendations against its use for the primary prevention of chronic conditions such as fragility fracture exist in both the US[11] and Canada.[12] We will also exclude off-label pharmaceuticals and dosages, natural health products, dietary supplements, calcitonin (no longer approved by Health Canada),[13] the 5mg per day dosage of alendronate (no longer recommended for osteoporosis treatment),[7, 14] and drugs used in combination. Observational studies examining harms of oral bisphosphonates as a class will be included if >90% of participants are taking alendronate or risedronate or if within-study subgroup analysis for these drugs is available. We will include studies where there is identical adjunct vitamin D and calcium use in both groups. Falls prevention or exercise programs are beyond the scope of this guideline, as are studies that are focused solely on complex interventions (e.g., pharmacotherapy + exercise), thus they will be excluded.

For KQ4, the intervention or exposure of interest is that participants be provided with information (e.g., BMD/medical fracture risk, scenarios, vignettes, education material, decision aids) containing the possible magnitude of benefits and/or harms of screening (using methods eligible for KQ1) or treatment (with denosumab or bisphosphonates) for fragility fractures or osteoporosis. Alternately, investigators could solicit participants’ perceptions of the magnitude of benefits and/or harms of screening or treatment where it is believed to be acceptable. The population may or may not have knowledge of their own medical fracture risk/BMD but must have at least some general scenario or background information on the benefits and harms of screening or treatment. Subgroups of interest include different presentations of information (e.g., magnitudes of effects, absolute versus relative effects, number of outcomes presented).

**Additional table 1.** Pharmacotherapies approved by Health Canada for the prevention of fragility fracture or treatment of osteoporosis that are included in the systematic review

| **Drug name** | **Approved dosages** | **Mode of administration** |
| --- | --- | --- |
| **Bisphosphonates** | | |
| Alendronate (Fosamax) | 10 mg daily  70 mg weekly | Oral |
| Alendronate (+vitamin D) (Fosavance) | 70 mg (Alendronate) & 2800 IU (Vit D) weekly  70 mg (Alendronate) & 5600 IU (Vit D) weekly | Oral |
| Risedronate (Actonel) | 5 mg daily  35 mg weekly  150 mg monthly | Oral |
| Risedronate (+calcium) | 35 mg weekly (Risedronate) & 500 mg calcium daily on day 2-7 | Oral |
| Zoledronic acid (Aclasta) | 5 mg per year | Intravenous infusion |
| **RANKL inhibitor** | | |
| Denosumab (Prolia) | 60 mg every 6 months | Subcutaneous injection |

RANKL: Receptor activator of nuclear factor kappa-Β ligand

Comparators

For KQ1a (benefits and harms of screening) the comparator of interest is no screening. Screened and unscreened study arms must have access to the same treatment, however studies will not be excluded based on treatment type (e.g., pharmacological therapy that is no longer the standard of care). For KQ1b (comparative effectiveness of screening approaches) the comparator of interest is screening using a different strategy (e.g., one- versus two-step) or fracture risk assessment tool. For KQ2 (accuracy of screening tests) the comparator is the observed or estimated (e.g., incorporating mortality) fracture rate (i.e., hip, all clinical/major osteoporotic fractures). For KQ3a (benefits of treatment) the comparator of interest is placebo. For KQ3b, we will also include studies with no treatment (versus placebo) as a comparator (i.e., controlled observational studies; for serious adverse events), or (for osteonecrosis of the jaw and atypical fracture) no comparison group. For KQ4 we will include non-active exposure (i.e., intervention without information about potential magnitude of benefits and/or harms), information on alternative screening or treatment strategies (of those eligible as interventions/exposures), or studies with no comparator.

Outcomes

To determine the preliminary outcomes for the review, the Task Force rated potential outcomes on a 10-point scale (0 to 9) based on their significance and impact within a population screening program to prevent fragility fractures. Outcomes rated between 4 and 6 are considered important and those rated 7 to 9 are considered critical to decision-making. Additional table 2 (below) shows outcome definitions and information related to baseline risk. Following the initial ratings, the working group met to reach consensus on the outcomes to be included. These outcomes were also rated by patients using surveys and focus groups conducted by the Knowledge Translation team at St. Michael's Hospital in Toronto, Ontario. The final outcome ratings were evaluated after considering the value ratings (i.e., important or critical) determined by the patients.

For KQ1 (benefits and harms of screening) and KQ3 (benefits and harms of treatment), the *critical outcomes* chosen by the Task Force are: hip fractures, fracture-related mortality, functionality and disability (including surrogate measures such as frailty measures or long-term care admissions if required), quality of life or wellbeing, all clinical fragility fractures, and serious adverse events. According to the International Conference on Harmonisation of Technical Requirements for Pharmaceuticals for Human Use, serious adverse events are defined as “any untoward medical occurrence that at any dose: results in death, is life-threatening, requires inpatient hospitalisation or prolongation of existing hospitalisation, results in persistent or significant disability/incapacity, or results in a congenital anomaly/birth defect”.[15] Apart from data on the number of people experiencing any serious adverse event, we will also include nine serious adverse event “sub-outcomes”: all serious cardiovascular events; serious cardiac rhythm disturbances (e.g., atrial fibrillation or ventricular arrhythmia); serious gastrointestinal events (excluding cancers); gastrointestinal cancers; atypical fractures; osteonecrosis of the jaw; fractures related to rebound effects of stopping treatment.

For KQ1 (benefits and harms of screening) and KQ3 (benefits and harms of treatment), the *important outcomes* chosen by the Task Force are all-cause mortality, overdiagnosis (relevant only to KQ1), discontinuations due to adverse events, and non-serious adverse events. Non-serious adverse events include any non-life threatening event (i.e., does not require hospitalization or result in disability) that occurs following treatment. For these outcomes we will rely on reports of the number of participants experiencing one or more non-serious adverse event (or ‘any adverse event’, if needed) as reported within the included studies. We do not anticipate that any study will report on overdiagnosis directly; apart from the relevant findings on treatment benefits and harms, we will extract data on variables (e.g., number screened, number considered to be at high risk [either by a set threshold or after shared decision-making], mean [with variance] predicted risk of fractures, treatment thresholds) that may be considered if the Task Force chooses to either calculate an estimate or discuss the outcome conceptually.

For KQ2 (accuracy of screening tests) the critical outcome of interest is calibration (i.e., accuracy of the absolute risk prediction; measured via expected versus observed fracture rate, ‘goodness-of-fit’ or Hosmer-Lemeshow test, calibration slope), because this outcome is most likely to be useful to clinicians in decision-making. We will report on calibration at 5- and 10-year (as applicable) expected rates, for hip and clinical fractures. We will report on total/average calibration (i.e., across all study participants) as well as calibration at varying levels of expected risk. Data on discrimination (i.e., ability of the tool to correctly distinguish between those who will or will not fracture based on particular thresholds; measured via area under the receiver operative characteristics curve and other measures of accuracy such as sensitivity and specificity), though not rated as a critical or important outcome, will be reported as it is presented in the USPSTF review that we are updating. We will not include new studies reporting only on discrimination.

For KQ4, outcomes of interest include any acceptability measures, such as willingness or intentions to screen or initiate treatment, acceptability of screening or initiating treatment, uptake of screening or treatment, absolute risk for fracture that would make treatment acceptable, and or suitable outcomes reported by study authors (e.g., intent to return for another screen, magnitude of benefits to make screening or treatment acceptable).

**Additional table 2.** Critical and important outcome definitions

| **Outcome** | **Definition** | **Baseline Risk or Subgroups** |
| --- | --- | --- |
| **Rating: Critical** | | |
| Hip fracture | A radiographically verified low trauma break in the upper portion of the femur (thigh bone) | Low trauma hip fractures among Canadians ≥50 years were estimated at 28,867 in the 2007/2008 fiscal year (based on hospital admissions).[16] Age-standardized hip fracture rates (all fracture types) were 496 per 100,000 Canadians aged ≥65 years in 2016 (based on hospital admissions).[17] |
| Fracture-related mortality | Deaths attributed to a fragility fracture | Since the risk of osteoporosis increases with age, comorbidities such as diabetes and hypertension are common and increase the risk of mortality; however, an estimated 26-28% of deaths can be attributed to the fragility fracture alone.[18] |
| Quality of life or well-being | Perceived physical health, psychological state, personal beliefs, social relationships and their relationship to salient features of their environment.[19] A related concept, well-being, assesses the positive aspects of a person’s life, such as positive emotions and life satisfaction.[20] |  |
| Functionality and disability (includes surrogate measures of frailty and long-term care admissions) | Measures of functional abilities of the individual (i.e. ability to perform daily physical tasks, self-care or social activities that provided enjoyment). Surrogate measures include admission to a long-term care facility (e.g., nursing home, rehabilitation centre) due to a fragility fracture. | Among Canadians ≥50 years, following a fragility fracture, 32% were released to long-term, chronic or rehab care compared to 13.2% prior to the fracture.[16] |
| All clinical fragility fractures | Fragility fractures are broken bones that result from a minor fall or normal activity that usually should not cause a fracture in healthy adults. Clinical fragility fractures are symptomatic and cause pain, disability or reduction in quality of life (i.e. excludes asymptomatic vertebral fractures or those found incidentally). Fragility fractures are associated with osteoporosis and usually occur at the hip, spine or wrist.[9] These fractures must be radiographically verified. | Low trauma fractures among Canadians ≥50 years were estimated at 57,413 in the 2007/2008 fiscal year (based on hospital admissions).[16] |
| Serious adverse events (includes surrogate measure of serious adverse (drug) reactions) | "A serious adverse event (experience) or reaction is any untoward medical occurrence that at any dose (a) results in death, (b) is life-threatening, (c) requires inpatient hospitalisation or prolongation of existing hospitalisation, (d) results in persistent or significant disability/ incapacity, or (e) is a congenital anomaly/birth defect."[15] | **Subgroups**  (a) All serious cardiovascular events (e.g. myocardial infarction, thromboembolic events, cardiovascular death, cerebrovascular accident/stroke)  (b) Serious cardiac rhythm disturbances (e.g. serious atrial fibrillation or ventricular arrhythmia)  (c) Serious gastrointestinal (GI) events (excluding GI cancers)  (d) GI cancer (e.g. esophageal, stomach)  (e) Atypical femoral fractures  (f) Osteonecrosis of the jaw  (g) Fractures related to rebound effects of stopping treatments |
| Calibration | Measure of accuracy of a model’s absolute risk prediction (i.e., expected versus observed fracture rate, ‘goodness-of-fit’ or Hosmer-Lemeshow test, calibration slope) |  |
| **Rating: Important** | | |
| Overdiagnosis | Overdiagnosed patients may be considered to be those who are at high risk of fracture – either according to a set threshold or by self-selection based on shared decision-making – but who would have never known that they were at high risk (i.e., would never have fractured) without screening. Using a shared decision-making perspective, overdiagnosis may be conceptualized as patients who had a risk assessment and following shared decision-making decided to start treatment but would never have fractured anyway. |  |
| All-cause mortality | Deaths due to any cause | In Canada there were 267,213 deaths from all causes in 2016 with a rate of 7.4 per 1,000 population.[21] |
| Non-serious adverse events (includes surrogate measures of all adverse events or adverse (drug) reactions) | A non-serious adverse event (experience) or reaction is any untoward medical occurrence at any dose. However it does not (a) result in death, (b) become life-threatening, (c) require inpatient hospitalisation or prolongation of existing hospitalisation, (d) result in persistent or significant disability/ incapacity, or (e) result in a congenital anomaly/birth defect. | May include “any non-serious adverse event” or “any adverse event” as reported in the individual studies. |
| Discontinuation due to adverse events or adverse (drug) reactions | Stopping treatment due to an adverse event or reaction that occurred during pharmaceutical usage. |  |

Setting

For all KQs the setting of interest is primary health care.[22] For KQ3b, we will include studies of adults in long-term care, but for all other KQs we will exclude studies of patients who are hospitalized or in long-term care because fracture risk and management in these settings differs from screening in primary care.[23, 24] As previously mentioned, for KQ2 the risk prediction tool must have been externally validated within a population in a very-high human development index country[2] with a similar hip fracture incidence to Canada.[25]

Study design and timing

For KQ1 (benefits and harms of screening) we will prioritize the inclusion of randomized controlled trials. We will require studies to have at least 6 months of follow-up to ensure that it will be possible to assess the occurrence of new fractures. Controlled clinical trials will be considered if randomized controlled trials provide insufficient evidence to inform a recommendation. We will exclude all other study designs.

For KQ2 (accuracy of screening tests) we will include prospective and retrospective cohort studies with a defined index screen (undertaken or using data assessed before the measurement of fractures). Randomized trials comparing two or more different index tests (all patients assessed for fractures) will also be included, but each arm assessed separately. We will exclude all other study designs. We will include studies with any duration follow-up but interpret their methodological quality in respect of the desired 5- and 10-year risk predictions.

For KQ3a (benefits of treatment) will include randomized controlled trials, and will require studies to have at least 6 months of follow-up. We will exclude all other study designs, including extensions of trials without an eligible comparator or with select populations (e.g., responders) of the original trial. For KQ3b (harms of treatment) we will include randomized controlled trials with at least 6 months of follow-up. For serious adverse events, we will also include observational studies including >1,000 participants. With the exception of osteonecrosis of the jaw and atypical fracture outcomes, observational studies must have a no treatment comparator as these outcomes are the least prone to attribution to other causes. We will exclude case studies and case series (where all participants had the outcome) and cross-sectional studies.

For KQ4 we will include any quantitative study design, and exclude qualitative studies. For all KQs we will include relevant dissertations, abstracts, and results published in clinical trials registers (latter two published in the past 2 years). We will exclude systematic reviews, meta-analyses, and pooled analyses.

Language and date of publication

For all KQs we will include studies published in English or French. For KQs 1 to 3 we will not impose any date limit; for KQ4 we will include studies published from 1995 to present to coincide with the approval of bisphosphonates in Canada.

**B. Data extraction items**

We will extract the following from each of the included studies:

- **Publication characteristics:** author(s); date of publication; country of origin; funding source; study design; sample size calculations;
- **Participant characteristics:** eligibility criteria; number assessed for eligibility, allocated to each study arm, screened and/or treated, assessed for outcomes; baseline characteristics related to our subgroups of interest (see Tables 1 to 4 in the main protocol document); proportion with previous fragility fracture; proportion with previous anti-osteoporosis medication exposure;
- **Interventions/exposures:** screening strategy including the approach (1-step vs. 2-step), fracture risk assessment tool used (e.g., variables and weighting of variables in the prediction model, handling of predictors in the model) and any available validation information, treatment threshold (KQ1); fracture rate in the validation cohort (KQ2); time span of prediction (e.g., 5- or 10-year risk), and intended timing of use (KQ2); pharmacological treatment including dose, timing, mode of administration, adjunctive treatments (KQ1 & 3); type, dose, and mode of delivery of information provided (KQ4);
- **Comparators:** details about no screening (e.g., usual care) group (KQ1a); alternate screening strategy including 1-step vs. 2-step approach (KQ1b), fracture risk assessment tool used (KQ1b and KQ2); ascertainment of fractures as the reference standard (KQ2); adjunctive treatments in the placebo or no treatment arm (KQ3); alternate or non-active exposure approach (KQ4);
- **Setting:** location of recruitment, screening, treatment; validation population (KQ2);
- **Outcomes:** definition used in study; measurement and ascertainment; timing (length of follow-up);
- **Analysis details:** available within-study subgroup analyses of interest, adjusted analyses; missing data and how these were handled; number analyzed for each outcome; method for estimating observed fracture rate (if applicable);
- **Quantitative findings related to the outcomes of interest:** number of events (or relative risks, rate ratios, odds or hazard ratios, and cumulative incidence data when events are unavailable) and sample size in each study arm, number deemed to be above the treatment threshold with fractures at follow-up (KQ1), mean risk of fracture in the population deemed to be at risk (KQ1), change from baseline or baseline and final scores for continuous variables (with measures of variability), calibration (e.g., expected vs. observed fractures, calibration slope) data related to prognostic model performance, acceptability measures (KQ4). For clinical fractures and all serious adverse events, we will preferentially extract dichotomous/binary data (number of participants experiencing one or more serious adverse events during follow-up); if unreported we will accept count data (number of total events) or sum the events across several serious adverse event outcomes to calculate a count of events experienced during follow-up. For each of the individual serious adverse events of interest (the “sub-outcomes”), we will consider whether or not the data represents dichotomous or count data and extract and analyze appropriately. For non-serious adverse events, we will only rely on dichotomous data, but will consider using data on “any adverse event” recognizing that some serious adverse events will be included.

**C. Description of the risk of bias or quality assessment tools used to appraise the included studies**

We will appraise the risk of bias in randomized controlled trials and clinical controlled trials across six domains (i.e., selection bias, performance bias, detection bias, attrition bias, reporting bias, other sources of bias) using the Cochrane Risk of Bias tool (2011 version).[26] For KQ3b (harms of treatment), we will consider additional questions from the McHarm tool[27] to inform our assessments of selective outcome reporting (i.e., whether all harms were collected and the total number of participants who experienced each type of harm was reported by study arm) and other sources of bias (i.e., whether the collection of harms data was active or passive). For trials published post-2009, we will search for study protocols (using Google and trial registries) to assist in informing our assessments for the selective outcome reporting domain. We will rate the overall (study- or outcome-level) risk of bias as low if all key domains are judged to be at low risk of bias, unclear if at least one key domain is judged to be at unclear risk of bias and none are at high risk of bias, and high if any key domain is judged to be at high risk of bias.

We will appraise the quality controlled cohort and case-control studies across four domains (i.e., sample selection, comparability, exposure [case-control only], and outcome [cohort only]) using the Newcastle-Ottawa Quality Assessment Scale.[28] For these studies, we will additionally assess the potential for selective outcome reporting (with incorporation of McHarm[27] domains as appropriate in KQ3b on harms of treatment). Despite the relative lack of protocols published for observational studies, we may appraise selective reporting to be at risk when expected outcomes (i.e., those defined as ‘critical’ or ‘important’ for patients and for decision-making) are not reported by the study authors.

We will appraise surveys/cross-sectional studies (KQ4) and uncontrolled cohort studies using the 14 questions contained within the Quality Assessment Tool for Observational Cohort and Cross-sectional Studies developed by the National Institutes of Health’s National Heart, Lung, and Blood Institute.[29] We will classify studies as low, moderate, or high risk of bias based on our judgment about the studies’ performance across the 14 questions.

We will appraise the risk of bias and applicability of prognostic accuracy studies across four domains (i.e., participant selection, predictors, outcome, analysis) using the signaling questions (i.e., excluding questions related only to model development) from the Prediction model Risk Of Bias Assessment Tool.[30,31] We will classify studies as low, moderate, or high risk of bias based on ratings for each domain.

**REFERENCES**

1. Prior JC, Langsetmo L, Lentle BC, Berger C, Goltzman D, Kovacs CS, et al. Ten-year incident osteoporosis-related fractures in the population-based Canadian Multicentre Osteoporosis Study — Comparing site and age-specific risks in women and men. Bone. 2015;71:237-43.

2. United Nations Development Programme (UNDP). Human development report 2016: Human development for everyone. 2016. <http://hdr.undp.org/sites/default/files/2016_human_development_report.pdf>. Accessed 30 Jan 2019.

3. Kanis JA, Odén A, McCloskey EV, Johansson H, Wahl DA, Cooper C. A systematic review of hip fracture incidence and probability of fracture worldwide. Osteoporos Int. 2012;23:2239-56.

4. Kanis JA, Johnell O, Oden A, Johansson H, Eisman JA, Fujiwara S, et al. The use of multiple sites for the diagnosis of osteoporosis. Osteoporos Int. 2006;17:527-34.

5. Leslie WD, Tsang JF, Caetano PA, Lix LM. Number of osteoporotic sites and fracture risk assessment: a cohort study from the Manitoba Bone Density Program. JBMR. 2009;22:476-83.

6. Viswanathan M, Reddy S, Berkman N, et al. Screening to prevent osteoporotic fractures: Updated evidence report and systematic review for the US Preventive Services Task Force. JAMA. 2018;319:2532-51.

7. Health Canada. Drug Product Database online query. 2018. <https://health-products.canada.ca/dpd-bdpp/index-eng.jsp>. Accessed 30 Jan 2019.

8. Papaioannou A, Morin S, Cheung AM, Atkinson S, Brown JP, Feldman S, et al. 2010 clinical practice guidelines for the diagnosis and management of osteoporosis in Canada: summary. CMAJ. 2010;182:1864-73.

9. Qaseem A, Forciea M, McLean RM, Denberg TD, for the Clinical Guidelines Committee of the American College of Physicians. Treatment of low bone density or osteoporosis to prevent fractures in men and women: A clinical practice guideline update from the American College of Physicians. Ann Intern Med. 2017;166:818-39.

10. Camacho PM, Petak SM, Binkley N, Clarke BL, Harris ST, Hurley DL, et al. American Association of Clinical Endocrinologists and American College of Endocrinology clinical practice guidelines for the diagnosis and treatment of postmenopausal osteoporosis—2016. Endocr Pract. 2016;22 Suppl 4:1-42.

11. Grossman DC, Curry SJ, Owens DK, Barry MJ, Davidson KW, Doubeni CA, et al. Hormone therapy for the primary prevention of chronic conditions in postmenopausal women: US Preventive Services Task Force recommendation statement. JAMA. 2017;318:2224-33.

12. Wathen CN, Feig DS, Feightner JW, Abramson BL, Cheung AM. Hormone replacement therapy for the primary prevention of chronic diseases: Recommendation statement from the Canadian Task Force on Preventive Health Care. CMAJ. 2004;170:1535-7.

13. Health Canada. Recalls and safety alerts: Synthetic calcitonin (salmon) nasal spray (NS) - market withdrawal of all products. 2013. <http://healthycanadians.gc.ca/recall-alert-rappel-avis/hc-sc/2013/34783a-eng.php>. Accessed 30 Jan 2019.

14. Accord Healthcare Inc. Product monograph: alendronate. 2018. <https://pdf.hres.ca/dpd_pm/00044117.PDF>. Accessed 30 Jan 2019.

15. International Conference on Harmonisation (ICH) of Technical Requirements for Registration of Pharmaceuticals for Human Use. Clinical safety data management: Definitions and standards for expedited reportings e2a. 1994. <https://www.ich.org/products/guidelines/efficacy/efficacy-single/article/clinical-safety-data-management-definitions-and-standards-for-expedited-reporting.html>. Accessed 30 Jan 2019.

16. Tarride JE, Hopkins RB, Leslie WD, Morin S, Adachi JD, Papaioannou A, et al. The burden of illness of osteoporosis in Canada. Osteoporos Int. 2012;23:2591-600.

17. Canadian Institute for Health Information. Indicator data: Hospitalized hip fracture event. 2017. <http://indicatorlibrary.cihi.ca/display/HSPIL/Hospitalized+Hip+Fracture+Event>. Accessed 30 Jan 2019.

18. Teng GG, Curtis eR, Saag KG. Mortality and osteoporotic fractures: is the link causal, and is it modifiable? Clin Exp Rheumatol. 2008;26:S125-S37.

19. World Health Organization. WHOQOL: measuring quality of life. 1997. <http://www.who.int/mental_health/publications/whoqol/en/>. Accessed 30 Jan 2019.

20. Office of Disease Prevention and Health Promotion, US Department of Health & Human Services. Healthy People 2020: Health-related quality of life and well-being. 2010. <https://www.healthypeople.gov/2020/about/foundation-health-measures/Health-Related-Quality-of-Life-and-Well-Being>. Accessed 30 Jan 2019.

21. Statistics Canada. Table 13-10-0710-01: Deaths and mortality rate, by age group. 2018. <https://www150.statcan.gc.ca/t1/tbl1/en/tv.action?pid=1310071001>. Accessed 30 Jan 2019.

22. Government of Canada. About primary health care. 2012. <https://www.canada.ca/en/health-canada/services/primary-health-care/about-primary-health-care.html>. Accessed 30 Jan 2019.

23. Khatib R, Santesso N, Pickard L, Osman O, Giangregorio L, Skidmore C, et al. Fracture risk in long term care: a systematic review and meta-analysis of prospective observational studies. BMC Geriatrics. 2014;14:130.

24. Warriner AH, Outman RC, Saag KG, Berry SD, Colon-Emeric C, Flood KL, et al. The management of osteoporosis among home health and long term care patients with a prior fracture. South Med J. 2009;102:397.

25. Kanis JA, Johnell O, De Laet C, Jonsson B, Oden A, Ogelsby AK. International variations in hip fracture probabilities: Implications for risk assessment. JBMR. 2002;17:1237-44.

26. Higgins JPT, Altman DG, Gøtzsche PC, Jüni P, Moher D, Oxman AD, et al. The Cochrane Collaboration’s tool for assessing risk of bias in randomised trials. BMJ. 2011;343.

27. Agency for Healthcare Research and Quality. Methods guide for effectiveness and comparative effectiveness reviews. Rockville, MD: Agency for Healthcare Research and Quality; 2011.

28. Wells GA, Shea B, O’Connell D, Peterson J, Welch V, Losos M, et al. The Newcastle-Ottawa Scale (NOS) for assessing the quality of nonrandomised studies in meta-analyses. 2009. http://www.ohri.ca/programs/clinical_epidemiology/oxford.asp. Accessed 30 Jan 2019

29. National Institute of Health National Heart, Lung, and Blood Institute. Study quality assessment tools: Quality assessment tool for observational cohort and cross-sectional studies. National Institute of Health, 2019. <https://www.nhlbi.nih.gov/health-topics/study-quality-assessment-tools>

30. Moons KM, Wolff RF, Riley RD, et al. Probast: A tool to assess risk of bias and applicability of prediction model studies: Explanation and elaboration. Ann Intern Med. 2019;170:W1-W33.

31. Wolff RF, Moons KM, Riley RD, et al. Probast: A tool to assess the risk of bias and applicability of prediction model studies. Ann Intern Med. 2019;170:51-8.
