# Supplementary material for: Screening to prevent fragility fractures among adults 40 years and older in primary care: protocol for a systematic review
Source: Syst Rev. 2019 Aug 23;8:216. doi: 10.1186/s13643-019-1094-5 (PMC6706906; doi:10.1186/s13643-019-1094-5)
Supplement: Supplementary file 4 — Identified systematic reviews with adverse events data from observational studies for KQ3b. This file contains a list of systematic reviews identified for integration in KQ3b. (DOCX 18 kb) [file 13643_2019_1094_MOESM4_ESM.docx]

**Additional file 4.** Identified systematic reviews with adverse events data from observational studies for KQ3b

1. Boleto G, Drame M, Lambrecht I, Eschard JP, Salmon JH. Disease-modifying anti-rheumatic drug effect of denosumab on radiographic progression in rheumatoid arthritis: a systematic review of the literature. Clin Rheumatol. 2017;36(8):1699-706.
2. Cai D, Qin J, Chen G, Feng W, Liu J. Bisphosphonates use and risk of gastric cancer: an updated meta-analysis of cohort and case-control studies. Minerva Med. 2017;108(5):464-72.
3. Chen LX, Ning GZ, Zhou ZR, Li YL, Zhang D, Wu QL, et al. The carcinogenicity of alendronate in patients with osteoporosis: evidence from cohort studies. PLoS ONE. 2015;10(4):e0123080.
4. Conwell LS, Chang AB. Bisphosphonates for osteoporosis in people with cystic fibrosis. Cochrane Database Syst Rev. 2014(3):CD002010.
5. Gauthier K, Bai A, Perras C, Cunningham J, Ahuja T, Richter T, et al. Denosumab, raloxifene, and zoledronic acid for the treatment of postmenopausal osteoporosis: clinical effectiveness and harms. CADTH Rapid Response Reports. 2012;02:02.
6. Gedmintas L, Solomon DH, Kim SC. Bisphosphonates and risk of subtrochanteric, femoral shaft, and atypical femur fracture: a systematic review and meta-analysis. J Bone Miner Res. 2013;28(8):1729-37.
7. Giusti A, Hamdy NA, Papapoulos SE. Atypical fractures of the femur and bisphosphonate therapy: A systematic review of case/case series studies. Bone. 2010;47(2):169-80. Epub 2010/05/25.
8. Howard PA, Barnes BJ, Vacek JL, Chen W, Lai SM. Impact of bisphosphonates on the risk of atrial fibrillation. Am J Muskuloskelet Drugs. 2010;10(6):359-67. Epub 2010/11/26.
9. Khan AA, Sandor GK, Dore E, Morrison AD, Alsahli M, Amin F, et al. Bisphosphonate associated osteonecrosis of the jaw. The Journal of rheumatology. 2009;36(3):478-90. Epub 2009/03/17.
10. Khow KS, Shibu P, Yu SC, Chehade MJ, Visvanathan R. Epidemiology and postoperative outcomes of atypical femoral fractures in older adults: a systematic review. J Nutr Health Aging. 2017;21(1):83-91.
11. Kim DH, Rogers JR, Fulchino LA, Kim CA, Solomon DH, Kim SC. Bisphosphonates and risk of cardiovascular events: a meta-analysis. PLoS ONE. 2015;10(4):e0122646.
12. Kim HY, Kim JW, Kim SJ, Lee SH, Lee HS. Uncertainty of current algorithm for bisphosphonate-related osteonecrosis of the jaw in population-based studies: a systematic review. J Bone Miner Res. 2017;32(3):584-91.
13. Kim SY, Kim MJ, Cadarette SM, et al. Bisphosphonates and risk of artial fibrillation: a meta-analysis. Arthritis Res Ther. 2010;12(1):R30.20170505
14. Lee S, Yin RV, Hirpara H, Lee NC, Lee A, Llanos S, et al. Increased risk for atypical fractures associated with bisphosphonate use. Fam Pract. 2015;32(3):276-81.
15. Lee SH, Chang SS, Lee M, Chan RC, Lee CC. Risk of osteonecrosis in patients taking bisphosphonates for prevention of osteoporosis: a systematic review and meta-analysis. Osteoporos Int. 2014;25(3):1131-9.
16. Liu J, Zhang HX, Lu XX, Hu JJ, Deng LF. Bisphosphonates and risk of subtrochanteric, femoral shaft, and atypical femur fracture: sensitivity and trim and fill studies. Genet Test Mol Biomarkers. 2014;18(2):117-22.
17. Liu L, Li C, Yang P, Zhu J, Gan D, Bu L, et al. Association between alendronate and atypical femur fractures: a meta-analysis. Endocr. 2015;4(1):58-64.
18. Loke YK, Jeevanantham V, Singh S. Bisphosphonates and atrial fibrillation: systematic review and meta-analysis. Drug safety. 2009;32(3):219-28. Epub 2009/04/03.
19. Mak A, Cheung MW, Ho RC, Cheak AA, Lau CS. Bisphosphonates and atrial fibrillation: Bayesian meta-analyses of randomized controlled trials and observational studies. BMC Musculoskelet Disord. 2009;10:113. Epub 2009/09/24.
20. McGowan K, McGowan T, Ivanovski S. Risk factors for medication‐related osteonecrosis of the jaws: A systematic review. Oral diseases. 2017;24(4):527-36.
21. Sharma A, Chatterjee S, Arbab-Zadeh A, Goyal S, Lichstein E, Ghosh J, et al. Risk of serious atrial fibrillation and stroke with use of bisphosphonates: evidence from a meta-analysis. Chest. 2013;144(4):1311-22.
22. Sharma A, Einstein AJ, Vallakati A, Arbab-Zadeh A, Walker MD, Mukherjee D, et al. Risk of atrial fibrillation with use of oral and intravenous bisphosphonates. Am J Cardiol. 2014;113(11):1815-21.
23. Solomon DH, Mercer E, Woo SB, Avorn J, Schneeweiss S, Treister N. Defining the epidemiology of bisphosphonate-associated osteonecrosis of the jaw: prior work and current challenges. Osteoporos Int. 2013;24(1):237-44.
24. Sun K, Liu JM, Sun HX, Lu N, Ning G. Bisphosphonate treatment and risk of esophageal cancer: a meta-analysis of observational studies. Osteoporos Int. 2013;24(1):279-86.
25. Wright E, Schofield PT, Molokhia M. Bisphosphonates and evidence for association with esophageal and gastric cancer: a systematic review and meta-analysis. BMJ Open. 2015;5(12):e007133.
26. Yang G, Hu H, Zeng R, Huang J. Oral bisphosphonates and the risk of colorectal cancer: a meta-analysis. J Clin Gastroenterol. 2013;47(9):741-8.
