# Supplementary material for: Screening to prevent fragility fractures among adults 40 years and older in primary care: protocol for a systematic review
Source: Syst Rev. 2019 Aug 23;8:216. doi: 10.1186/s13643-019-1094-5 (PMC6706906; doi:10.1186/s13643-019-1094-5)
Supplement: Supplementary file 5 — Search strategies. This file contains the planned search strategies for the review. (DOCX 46 kb) [file 13643_2019_1094_MOESM5_ESM.docx]

**Additional file 5.** Search strategies

**Key Question 1**: Benefits and harms of screening

**Ovid MEDLINE(R) and Epub Ahead of Print, In-Process & Other Non-Indexed Citations and Daily 1946 to present**

| 1 Bone Density/ (49651)  2 Decalcification, Pathologic/ (285)  3 Fractures, Bone/ (61222)  4 Osteoporosis/ (41540)  5 Osteoporosis, Postmenopausal/ (12538)  6 Osteoporotic Fractures/ (4358)  7 (bone* adj2 (broken or fracture*)).tw,kf. (14289)  8 (bone* adj2 (brittle* or fragil*)).tw,kf. (2622)  9 (bone* adj2 (content* or densit* or loss* or mass)).tw,kf. (82110)  10 (bone* adj1 health*).tw,kf. (6648)  11 (fracture* adj2 (fragil* or risk*)).tw,kf. (16274)  12 osteoporo*.tw,kf. (70550)  13 or/1-12 [Combined MeSH & text words for fragility fractures] (205219)  14 Absorptiometry, Photon/ (21151)  15 Mass Screening/ (95622)  16 Risk Assessment/ (234752)  17 a#sorptiometry.tw,kf. (23543)  18 ((assess* or estimat* or predict*) adj3 risk*).tw,kf. (180105)  19 ((densitometry or photodensitometry) adj3 (radiograph* or x-ray* or xray*)).tw,kf. (523)  20 ((DEXA or DXA) adj2 scan*).tw,kf. (2029)  21 screen*.tw,kf. (666961)  22 or/14-21 [Combined MeSH & text words for screening] (1064443)  23 and/13,22 [Combined concepts for fragility fractures & screening] (32383)  24 exp "Controlled Clinical Trials as Topic"/ (127040)  25 clinical trial.pt. (513635)  26 controlled clinical trial.pt. (92800)  27 randomized controlled trial.pt. (472632)  28 (nonrandom* or nRCT*).ab. (17297)  29 random*.ab. (988464)  30 trial.ti. (191474)  31 or/24-30 (1493381)  32 exp animals/ not humans.sh. (4525184)  33 31 not 32 [Pragmatic RCT filter – not validated] (1376329)  34 and/23,33 [RCT filter applied] (4755)  35 (2016* or 2017* or 2018* or 2019*).dt. (3611477)  36 34 and 35 (635)  37 limit 34 to yr="2016-Current" (715)  38 36 or 37 [Date range limit applied] (723)  39 remove duplicates from 38 (720) |
| --- |

**Ovid Embase**

| 1 exp bone density/ (85762)  2 osteoporosis/ (106551)  3 postmenopause osteoporosis/ (13618)  4 fracture/ (77283)  5 fragility fracture/ (16148)  6 (bone* adj2 (broken or fracture*)).tw,kw. (19339)  7 (bone* adj2 (brittle* or fragil*)).tw,kw. (4148)  8 (bone* adj2 (content* or densit* or loss* or mass)).tw,kw. (114647)  9 (bone* adj1 health*).tw,kw. (11029)  10 (fracture* adj2 (fragil* or risk*)).tw,kw. (27326)  11 osteoporo*.tw,kw. (112090)  12 or/1-11 [Combined MeSH & text words for fragility fractures] (286341)  13 photon absorptiometry/ (3656)  14 mass screening/ (51376)  15 risk assessment/ (497801)  16 a#sorptiometry.tw,kw. (31692)  17 ((assess* or estimat* or predict*) adj3 risk*).tw,kw. (263372)  18 ((densitometry or photodensitometry) adj3 (radiograph* or x-ray* or xray*)).tw,kw. (729)  19 ((DEXA or DXA) adj2 scan*).tw,kw. (5263)  20 screen*.tw,kw. (942431)  21 or/13-20 [Combined MeSH & text words for screening] (1594040)  22 and/12,21 [Combined concepts for fragility fractures & screening] (48291)  23 exp "controlled clinical trial (topic)"/ (162793)  24 clinical trial/ (951658)  25 controlled clinical trial/ (458809)  26 exp randomized controlled trial/ (537623)  27 (nonrandom* or nRCT*).ab. (19677)  28 random*.ab. (1348006)  29 trial.ti. (263615)  30 or/23-29 (2253613)  31 exp animal/ or exp animal experimentation/ or exp animal model/ or exp animal experiment/ or nonhuman/ or exp vertebrate/ (25525597)  32 exp human/ or exp human experimentation/ or exp human experiment/ (19388583)  33 31 not 32 (6137928)  34 30 not 33 [ANIMAL-ONLY REMOVED] (2064491)  35 and/22,34 [RCT filter applied] (8138)  36 (2016* or 2017* or 2018* or 2019*).dc. (5304693)  37 35 and 36 (1882)  38 limit 35 to yr="2016-Current" (1663)  39 37 or 38 [Date range limit applied] (1900)  40 remove duplicates from 39 (1882) |
| --- |

**Cochrane Library**

| ID Search Hits  #1 [mh "Bone Density"] 4411  #2 [mh "Decalcification, Pathologic"] 4  #3 [mh ^"Fractures, Bone"] 1817  #4 [mh ^Osteoporosis] 1966  #5 [mh "Osteoporosis, Postmenopausal"] 1922  #6 [mh "Osteoporotic Fractures"] 254  #7 (bone* NEAR/2 (broken or fracture*)):ti,ab,kw 2639  #8 (bone* NEAR/2 (brittle* or fragil*)):ti,ab,kw 128  #9 (bone* NEAR/2 (content* or densit* or loss* or mass)):ti,ab,kw 12074  #10 (bone* NEAR/1 health*):ti,ab,kw 842  #11 (fracture* NEAR/2 (fragil* or risk*)):ti,ab,kw 1903  #12 osteoporo*:ti,ab,kw 8048  #13 {or #1-#12} 17441  #14 [mh "Absorptiometry, Photon"] 1787  #15 [mh ^"Mass Screening"] 2931  #16 [mh ^"Risk Assessment"] 8345  #17 (absorptiometry or asorptiometry):ti,ab,kw 4816  #18 ((assess* or estimat* or predict*) NEAR/3 risk*):ti,ab,kw 25575  #19 ((densitometry or photodensitometry) NEAR/3 (radiograph* or (x NEAR ray*) or xray*)):ti,ab,kw 39  #20 ((DEXA or DXA) NEAR/2 scan*):ti,ab,kw 497  #21 screen*:ti,ab,kw 42081  #22 {or #14-#21} 69804  #23 #13 AND #22 with Cochrane Library publication date Between Jan 2016 and Dec 2019 1297  #24 #13 AND #22 with Publication Year from 2016 to 2019, in Trials 811  #25 #23 OR #24 1298  DSR – 39  CENTRAL - 1259 |
| --- |

**Key Question 2:** Accuracy of screening tests

**Ovid MEDLINE(R) and Epub Ahead of Print, In-Process & Other Non-Indexed Citations and Daily 1946 to present**

| 1 Fractures, Bone/ (61674)  2 Fractures, Spontaneous/ (7201)  3 Hip Fractures/ (14730)  4 Osteoporotic Fractures/ (4584)  5 ((bone? or hip?) adj3 (break* or broke? or fractur*)).tw,kf. (37805)  6 ((spontaneous* or pathologic*) adj2 fractur*).tw,kf. (6585)  7 (fragility adj2 fractur*).tw,kf. (3258)  8 (osteoporo* adj2 fractur*).tw,kf. (10849)  9 or/1-8 [Combined MeSH & text words for fractures] (113776)  10 Absorptiometry, Photon/ (21382)  11 Algorithms/ (234652)  12 Risk/ (118222)  13 Risk Assessment/ (239434)  14 "Risk Evaluation and Mitigation"/ (13)  15 Risk Factors/ (760084)  16 "Surveys and Questionnaires"/ (421300)  17 a#sorptiometr*.tw,kf. (24089)  18 (absolute adj2 risk?).tw,kf. (6847)  19 algorithm*.tw,kf. (222011)  20 ((assess* or calculat* or estimat* or predict*) adj3 risk?).tw,kf. (193662)  21 ((assess* or predict* or prognos*) adj2 (instrument* or test* or tool*)).tw,kf. (107790)  22 ((densitometr* or photodensitometr* or photo-densitometr*) adj3 (radiograph* or x-ray* or xray*)).tw,kf. (625)  23 ((DEXA or DXA) adj2 scan*).tw,kf. (2073)  24 ((factor* or index* or indices* or scale* or scor* or stratif* or test*) adj3 risk?).tw,kf. (602848)  25 (fractur* adj3 predict*).tw,kf. (3377)  26 (fractureindex or fracture index).tw,kf. (82)  27 FRAX.tw,kf. (1141)  28 GARVAN.tw,kf. (50)  29 ((predict* or prognos*) adj2 score*).tw,kf. (17501)  30 (scoring adj2 system?).tw,kf. (28992)  31 ((predict* or prognos*) adj2 model*).tw,kf. (88372)  32 Qfracture*.tw,kf. (36)  33 questionnaire*.tw,kf. (460206)  34 (relative adj2 risk?).tw,kf. (74230)  35 (predict* adj2 (guide or guides or rule or rules)).tw,kf. (3439)  36 or/10-35 [Combined MeSH & text words for tests & risk assessment] (2513901)  37 and/9,36 [Combined concepts of fractures & assessment] (24394)  38 Calibration/ (36119)  39 Reference Values/ (156484)  40 exp "Sensitivity and Specificity"/ (548004)  41 exp Survival Analysis/ (270113)  42 ((accurac* or reliability or validity or value*) adj2 predict*).tw,kf. (142786)  43 ((accurac* or effectiveness or efficac* or error* or perform* or reliability or use? or validity or value* or yield*) adj2 diagnostic*).tw,kf. (116838)  44 ((auroc or hsroc or roc or sroc) adj1 (analys#s or curve?)).tw,kf. (39654)  45 calibrat*.tw,kf. (92477)  46 "goodness-of-fit".tw,kf. (7269)  47 Hosmer-Lemeshow.tw,kf. (1721)  48 (c-statistic? or cstatistic?).tw,kf. (5167)  49 (detection adj2 (limit* or rate*)).tw,kf. (124901)  50 discriminat*.tw,kf. (215167)  51 (false adj1 (negative* or positive*)).tw,kf. (71908)  52 gold standard*.tw,kf. (59187)  53 likelihood ratio*.tw,kf. (14207)  54 Odds Ratio/ (85183)  55 odds ratio*.tw,kf. (242489)  56 ((post-test or posttest or pre-test or pretest) adj1 probabilit*).tw,kf. (2827)  57 reference standard*.tw,kf. (15565)  58 receiver operat*.tw,kf. (68668)  59 sensitivity.tw,kf. (736209)  60 specificity.tw,kf. (430554)  61 threshold*.tw,kf. (240475)  62 Validation Studies/ (94080)  63 validat*.tw,kf. (452839)  64 exp Forecasting/ (81575)  65 forecast*.tw,kf. (15635)  66 or/38-65 [Pragmatic prognosis filter - not validated] (3040415)  67 and/37,66 [Prognosis filter applied] (6665)  68 Adolescent/ not Adult/ (632507)  69 exp Child/ not exp Adult/ (1151413)  70 exp Infant/ not exp Adult/ (800647)  71 67 not (68 or 69 or 70) [Exclude Pediatric Population] (6470)  72 (2016* or 2017* or 2018* or 2019*).dt. (3925462)  73 71 and 72 (1329)  74 limit 71 to yr="2016-Current" (1412)  75 73 or 74 [Date range limit applied] (1426)  76 remove duplicates from 75 (1422) |
| --- |

**Ovid Embase**

| 1 fracture/ (77283)  2 pathologic fracture/ (7850)  3 exp hip fracture/ (34287)  4 ((bone? or hip?) adj3 (break* or broke? or fractur*)).tw,kw. (51615)  5 ((spontaneous* or pathologic*) adj2 fractur*).tw,kw. (8918)  6 fragility fracture/ (16148)  7 (fragility adj2 fractur*).tw,kw. (6147)  8 (osteoporo* adj2 fractur*).tw,kw. (17469)  9 or/1-8 [Combined MeSH & text words for fractures] (148122)  10 photon absorptiometry/ (3656)  11 algorithm/ (240877)  12 risk/ (497349)  13 fall risk/ (3616)  14 risk assessment/ (497801)  15 fall risk assessment/ (1484)  16 "risk evaluation and mitigation strategy"/ (191)  17 risk factor/ (928441)  18 questionnaire/ (616419)  19 a#sorptiometry.tw,kw. (31692)  20 (absolute adj2 risk).tw,kw. (8867)  21 algorithm*.tw,kw. (274842)  22 ((assess* or calculat* or estimat* or predict*) adj3 risk*).tw,kw. (276980)  23 ((assess* or predict* or prognos*) adj2 (instrument* or test* or tool*)).tw,kw. (152917)  24 ((densitometr* or photodensitometr* or photo-densitometr*) adj3 (radiograph* or x-ray* or xray*)).tw,kw. (842)  25 ((DEXA or DXA) adj2 scan*).tw,kw. (5263)  26 ((factor* or index* or indices* or scale* or scor* or stratif* or test*) adj3 risk*).tw,kw. (899176)  27 (fracture* adj3 predict*).tw,kw. (5101)  28 (fractureindex or fracture index).tw,kw. (139)  29 FRAX.tw,kw. (2931)  30 GARVAN.tw,kw. (98)  31 ((predict* or prognos*) adj2 score*).tw,kw. (29940)  32 (scoring adj2 system?).tw,kw. (46056)  33 ((predict* or prognos*) adj2 model*).tw,kw. (115458)  34 Qfracture*.tw,kw. (82)  35 questionnaire*.tw,kw. (663681)  36 (relative adj2 risk?).tw,kw. (92521)  37 (predict* adj2 (guide or guides or rule or rules)).tw,kw. (4479)  38 or/10-37 [Combined MeSH & text words for tests & risk assessment] (3384951)  39 and/9,38 [Combined concepts of fractures & assessment] (45875)  40 calibration/ (63303)  41 exp reference value/ (124630)  42 "sensitivity and specificity"/ (317890)  43 survival analysis/ (13821)  44 predictive value/ (143846)  45 ((accurac* or reliability or validity or value*) adj2 predict*).tw,kw. (203068)  46 ((accurac* or effectiveness or efficac* or error* or perform* or reliability or use* or validity or value* or yield*) adj2 diagnostic*).tw,kw. (177088)  47 ((auroc or hsroc or roc or sroc) adj1 (analys#s or curve?)).tw,kw. (73447)  48 calibrat*.tw,kw. (115784)  49 "goodness-of-fit".tw,kw. (9315)  50 Hosmer-Lemeshow.tw,kw. (2851)  51 (c-statistic? or cstatistic?).tw,kw. (8964)  52 (detection adj2 (limit* or rate*)).tw,kw. (153930)  53 discriminat*.tw,kw. (260542)  54 false negative result/ (15719)  55 false positive result/ (25433)  56 (false adj1 (negative* or positive*)).tw,kw. (96669)  57 gold standard/ (48157)  58 gold standard*.tw,kw. (96450)  59 likelihood ratio*.tw,kw. (19075)  60 odds ratio/ (14263)  61 odds ratio*.tw,kw. (302115)  62 ((post-test or posttest or pre-test or pretest) adj1 probabilit*).tw,kw. (4563)  63 reference standard*.tw,kw. (21324)  64 receiver operat*.tw,kw. (91936)  65 sensitivity.tw,kw. (938540)  66 specificity.tw,kw. (548159)  67 threshold*.tw,kw. (302142)  68 validation study/ (75383)  69 validat*.tw,kw. (657490)  70 "prediction and forecasting"/ (18600)  71 forecasting/ (43240)  72 forecast*.tw,kw. (19210)  73 or/40-72 [Pragmatic prognosis filter - not validated] (3310321)  74 and/39,73 [Prognosis filter applied] (9268)  75 exp adolescent/ not exp adult/ (523142)  76 exp child/ not exp adult/ (1772939)  77 fetus/ not exp adult/ (151944)  78 74 not (75 or 76 or 77) [Exclude Pediatric Population] (8996)  79 (2016* or 2017* or 2018* or 2019*).dc. (5304693)  80 78 and 79 (2494)  81 limit 78 to yr="2016-Current" (2255)  82 80 or 81 [Date range limit applied] (2518)  83 remove duplicates from 82 (2476) |
| --- |

**Cochrane Library**

| ID Search Hits  #1 [mh ^"Fractures, Bone"] 1817  #2 [mh "Fractures, Spontaneous"] 123  #3 [mh "Hip Fractures"] 1429  #4 [mh "Osteoporotic Fractures"] 254  #5 ((bone* or hip*) NEAR/3 (break* or broke? or fractur*)):ti,ab,kw 5507  #6 ((spontaneous* or pathologic*) NEAR/2 fractur*):ti,ab,kw 402  #7 (fragility NEAR/2 fractur*):ti,ab,kw 669  #8 (osteoporo* NEAR/2 fractur*):ti,ab,kw 1104  #9 {or #1-#8} 6859  #10 [mh "Absorptiometry, Photon"] 1787  #11 [mh ^Algorithms] 3334  #12 [mh ^Risk] 3202  #13 [mh ^"Risk Assessment"] 8345  #14 [mh "Risk Evaluation and Mitigation"] 0  #15 [mh "Risk Factors"] 23569  #16 [mh ^"Surveys and Questionnaires"] 23781  #17 (absorptiometry or asorptiometry):ti,ab,kw 4816  #18 (absolute NEAR/2 risk):ti,ab,kw 1280  #19 algorithm*:ti,ab,kw 9501  #20 ((assess* or calculat* or estimat* or predict*) NEAR/3 risk*):ti,ab,kw 26354  #21 ((assess* or predict* or prognos*) NEAR/2 (instrument* or test* or tool*)):ti,ab,kw 9370  #22 ((densitometr* or photodensitometr* or photo-densitometr*) NEAR/3 (radiograph* or (x NEXT ray*) or xray*)):ti,ab,kw 44  #23 ((DEXA or DXA) NEAR/2 scan*):ti,ab,kw 497  #24 ((factor* or index* or indices* or scale* or scor* or stratif* or test*) NEAR/3 risk*):ti,ab,kw 58288  #25 (fractur* NEAR/3 predict*):ti,ab,kw 142  #26 (fractureindex or "fracture index"):ti,ab,kw 7  #27 FRAX:ti,ab,kw 121  #28 GARVAN:ti,ab,kw 3  #29 ((predict* or prognos*) NEAR/2 score*):ti,ab,kw 1598  #30 (scoring NEAR/2 system*):ti,ab,kw 13513  #31 ((predict* or prognos*) NEAR/2 model*):ti,ab,kw 3203  #32 Qfracture*:ti,ab,kw 0  #33 questionnaire*:ti,ab,kw 79774  #34 (relative NEAR/2 risk*):ti,ab,kw 8807  #35 (predict* NEAR/2 (guide or guides or rule or rules)):ti,ab,kw 211  #36 {or #10-#35} 185095  #37 #9 and #36 2196  #38 [mh Calibration] 340  #39 [mh "Reference Values"] 9282  #40 [mh "Sensitivity and Specificity"] 15925  #41 [mh "Survival Analysis"] 19221  #42 ((accurac* or reliability or validity or value*) NEAR/1 predict*):ti,ab,kw 14994  #43 ((accurac* or effectiveness or efficac* or error* or perform* or reliability or use* or validity or value* or yield*) NEAR/2 diagnostic*):ti,ab,kw 12063  #44 ((auroc or hsroc or roc or sroc) NEAR/1 (analysis or analyses or curve*)):ti,ab,kw 2796  #45 calibrat*:ti,ab,kw 2886  #46 "goodness-of-fit":ti,ab,kw 225  #47 "Hosmer-Lemeshow":ti,ab,kw 86  #48 ((c NEXT statistic*) or cstatistic*):ti,ab,kw 489  #49 (detection NEAR/2 (limit* or rate*)):ti,ab,kw 2782  #50 discriminat*:ti,ab,kw 7296  #51 (false NEAR/1 (negative* or positive*)):ti,ab,kw 2869  #52 (gold NEXT standard*):ti,ab,kw 4742  #53 (likelihood NEXT ratio*):ti,ab,kw 642  #54 [mh "Odds Ratio"] 2908  #55 (odds NEXT ratio*):ti,ab,kw 17883  #56 (("post-test" or posttest or "pre-test" or pretest) NEAR/1 probabilit*):ti,ab,kw 183  #57 (reference NEXT standard*):ti,ab,kw 1263  #58 (receiver NEXT operat*):ti,ab,kw 4643  #59 sensitivity:ti,ab,kw 46336  #60 specificity:ti,ab,kw 20064  #61 threshold*:ti,ab,kw 18108  #62 "Validation Studies":pt 2399  #63 validat*:ti,ab,kw 24424  #64 [mh Forecasting] 507  #65 forecast*:ti,ab,kw 726  #66 {or #38-#65} 150889  #67 #37 AND #66 466  #68 [mh Adolescent] NOT [mh Adult] 98384  #69 [mh Child] NOT [mh Adult] 617  #70 [mh Infant] NOT [mh Adult] 15067  #71 #67 not (#68 or #69 or #70) with Cochrane Library publication date Between Jan 2016 and Dec 2019 145  #72 #71 with Publication Year from 2016 to 2019, in Trials 94  #73 #71 or #72 145  DSR – 7  CENTRAL - 138 |
| --- |

**Key Question 3:** Benefits and harms of treatment

**Ovid MEDLINE(R) and Epub Ahead of Print, In-Process & Other Non-Indexed Citations and Daily 1946 to present**

| 1 Alendronate/ (3541)  2 (alendronate or alenato$2 or alend$2 or alendros$2 or alovell$2 or arendal$2 or bifemelan$2 or bifosa$2 or binosto$2 or bonapex$2 or defixal$2 or dronal$2 or endronax$2 or eucalen$2 or fixopan$2 or fosalan$2 or fosamax$2 or fosmin$2 or fosval$2 or marvil$2 or maxibone$2 or "mk 0217" or "mk 217" or mk0217 or mk217 or neobon$2 or oncalst$2 or onclast$2 or osdron$2 or osdronat$2 or oseotenk$2 or osficar$2 or oslene$2 or osteofar$2 or osteofos$2 or osteopor$2 or osteosan$2 or osteovan$2 or osticalcin$2 or porosal$2 or teiroc$2 or tibolene$2 or voroste$2).tw,kf. (4586)  3 Denosumab/ (1342)  4 (denosumab or "amg 162" or amg162 or amgiva$2 or prolia$2 or xgeva$2).tw,kf. (2360)  5 Diphosphonates/ (15421)  6 (biphosphonate or biphosphonates or bisphosphonate or bisphosphonates or bisphosphonic acid derivative* or diphosphonate or diphosphonates or diphosphonic acid derivative*).tw,kf. (19841)  7 Risedronic Acid/ (1144)  8 (actonel$2 or atelvia$2 or benet$2 or "ne 58095" or ne58095 or optinate$2 or ribastamin$2 or risedronate or risedronic acid).tw,kf. (1732)  9 Zoledronic Acid/ (3182)  10 (aclasta$2 or "cgp 42446" or "cgp 42446a" or cgp42446 or cgp42446a or orazol$2 or reclast$2 or "zol 446" or zol446 or zoledronate or zoledronic acid or zomera$2 or zometa$2).tw,kf. (4354)  11 Osteoporosis/dt [drug therapy] (8926)  12 Osteoporosis, Postmenopausal/dt [drug therapy] (4433)  13 Osteoporotic Fractures/pc [prevention and control] (1304)  14 or/1-13 [OSTEOPOROTIC DRUGS OF INTEREST, DRUG THERAPY/PREVENTION] (38124)  15 ae.fs. [adverse effects free-floating subheading] (1640434)  16 co.fs. [complications free-floating subheading] (1890115)  17 to.fs. [toxicity free-floating subheading] (404655)  18 exp "Drug-Related Side Effects and Adverse Reactions"/ (109362)  19 (ADR or ADRs or ADE or ADEs).tw,kf. (12334)  20 Safety/ (38467)  21 Patient Safety/ (16547)  22 Patient Harm/ (126)  23 (safe* or unsafe*).tw,kf. (786394)  24 (side effect* or tolerability or toxicity or treatment emergent or undesirable effect*).tw,kf. (611457)  25 (adverse adj2 (effect or effects or event or events or experience* or incident or incidents or outcome or outcomes or reaction or reactions)).tw,kf. (391672)  26 (injurious adj2 (effect or effects or event or events or experience* or incident or incidents or outcome or outcomes or reaction or reactions)).tw,kf. (11904)  27 (undesirable adj2 (effect or effects or event or events or experience* or incident or incidents or outcome or outcomes or reaction or reactions)).tw,kf. (7304)  28 Risk/ (118412)  29 risk*.ti. (442673)  30 (harm or harmed or harming or harms or harmful*).tw,kf. (105081)  31 (adverse* or complication* or injurious or treatment outcome* or undesirable or tolerability or interaction or interactions or reaction or reactions or tolerability or toxic).ti. (654783)  32 Patient Dropouts/ (7844)  33 ((discontinu* or drop-out* or dropout* or withdr*) adj2 (participant* or patient* or subject*)).tw,kf. (18077)  34 or/15-33 [GENERAL HARMS, ADVERSE EVENTS TERMS] (5579910)  35 Osteoporotic Fractures/ci [chemically induced] (141)  36 Arrhythmias, Cardiac/ep, et, ci [Epidemiology, Etiology, Chemically Induced] (23464)  37 Atrial Fibrillation/ep, et, ci [Epidemiology, Etiology, Chemically Induced] (11824)  38 Cardiovascular Diseases/ep, et, ci [Epidemiology, Etiology, Chemically Induced] (60519)  39 Colorectal Neoplasms/ep, et, ci [Epidemiology, Etiology, Chemically Induced] (10312)  40 Esophageal Neoplasms/ep, et, ci [Epidemiology, Etiology, Chemically Induced] (6197)  41 Femoral Fractures/ep, et, ci [Epidemiology, Etiology, Chemically Induced] (2804)  42 Gastrointestinal Diseases/ep, et, ci [Epidemiology, Etiology, Chemically Induced] (12746)  43 Jaw Diseases/ep, et, ci [Epidemiology, Etiology, Chemically Induced] (1548)  44 Osteonecrosis/ep, et, ci [Epidemiology, Etiology, Chemically Induced] (3597)  45 exp Myocardial Ischemia/ep, et, ci [Epidemiology, Etiology, Chemically Induced] (92616)  46 Stomach Neoplasms/ep, et, ci [Epidemiology, Etiology, Chemically Induced] (10295)  47 Stroke/ep, et, ci [Epidemiology, Etiology, Chemically Induced] (32056)  48 (arrythmia* or arrhythmia* or dysrhythmia*).tw,kf. (87421)  49 ((atrial or atrium or auricular) adj fibril*).tw,kf. (64487)  50 ((cancer or cancers or carcinoma* or malignan* or metasta* or neoplasm* or tumor or tumors or tumour or tumours or adenoma* or adenocarcinoma* or adeno-carcinoma* or carcinosarcoma* or carcino-sarcoma*) adj3 (colon or colonic or colorectal* or colo-rectal* or esophag* or oesophag* or pharynx-esophag* or gastric or gastro* or rectal* or rectum or stomach)).tw,kf. (351534)  51 ((cardio* or cardiac or heart) adj2 (death* or disease* or event* or fatal* or incident? or mortalit*)).tw,kf. (414595)  52 ((complain* or effect* or symptom*) adj2 (gastric or gastro* or digesti* or stomach)).tw,kf. (33110)  53 ((femoral* or femur*) adj2 (break or breaks or breaking or broken or fracture*)).tw,kf. (18020)  54 ((infarct* or isch?emi*) adj1 (cardio* or cardiac or heart or myocardial or myo-cardial)).tw,kf. (249572)  55 heart attack?.tw,kf. (5207)  56 ((jaw or jaws or mandib*) adj3 (osteo-necro* or osteonecro*)).tw,kf. (2759)  57 (stroke or strokes or CVA or apople*).tw,kf. (231172)  58 ((brain or cerebral or cerebrum) adj (accident? or attack? or insult* or isch?emic attack? or vascular accident? or vascular insufficienc*)).tw,kf. (2124)  59 (cerebrovascular adj (accident? or arrest? or failure? or injury or injuries or insufficienc* or insult?)).tw,kf. (8131)  60 (isch?emic adj (cerebral attack? or seizure?)).tw,kf. (67)  61 or/35-60 [SPECIFIC DRUG HARMS] (1350887)  62 34 or 61 [GENERAL OR SPECIFIC DRUG HARMS] (6397481)  63 14 and 62 [DRUGS OF INTEREST - GENERAL AND SPECIFIC HARMS] (18433)  64 Bisphosphonate-Associated Osteonecrosis of the Jaw/ (1017)  65 63 or 64 [DRUGS OF INTEREST - GENERAL AND SPECIFIC HARMS, SPECIFIC MESH] (18548)  66 (controlled clinical trial or randomized controlled trial or pragmatic clinical trial or equivalence trial).pt. (568609)  67 clinical trial.pt. (515621)  68 exp "Controlled Clinical Trials as Topic"/ (130305)  69 (randomi#ed or randomi#ation? or randomly or RCT or placebo*).tw,kf. (902685)  70 ((singl* or doubl* or trebl* or tripl*) adj (mask* or blind* or dumm*)).tw,kf. (162997)  71 trial.ti. (196538)  72 or/66-71 [RCT FILTER] (1400492)  73 65 and 72 [RCTs] (4047)  74 controlled clinical trial.pt. (93025)  75 Controlled Clinical Trial/ or Controlled Clinical Trials as Topic/ (98389)  76 (control* adj2 trial).tw,kf. (139947)  77 Non-Randomized Controlled Trials as Topic/ (476)  78 (nonrandom* or non-random* or quasi-random* or quasi-experiment*).tw,kf. (50888)  79 (nRCT or non-RCT).tw,kf. (257)  80 Controlled Before-After Studies/ (383)  81 (control* adj3 ("before and after" or "before after")).tw,kf. (3965)  82 Interrupted Time Series Analysis/ (553)  83 time series.tw,kf. (27235)  84 (pre- adj3 post-).tw,kf. (72758)  85 (pretest adj3 posttest).tw,kf. (4916)  86 Historically Controlled Study/ (152)  87 (control* adj2 study).tw,kf. (159512)  88 Control Groups/ (1614)  89 (control* adj2 group?).tw,kf. (461349)  90 or/74-79 [NON-RCT FILTER] (281004)  91 65 and 90 [NON-RCTs] (769)  92 exp Cohort Studies/ (1844701)  93 cohort?.tw,kf. (507404)  94 Retrospective Studies/ (741924)  95 (longitudinal or prospective or retrospective).tw,kf. (1140444)  96 ((followup or follow-up) adj (study or studies)).tw,kf. (48665)  97 Observational study.pt. (60307)  98 (observation$2 adj (study or studies)).tw,kf. (92576)  99 ((population or population-based) adj (study or studies or analys#s)).tw,kf. (16112)  100 ((multidimensional or multi-dimensional) adj (study or studies)).tw,kf. (106)  101 Comparative Study.pt. (1826814)  102 ((comparative or comparison) adj (study or studies)).tw,kf. (107864)  103 exp Case-Control Studies/ (984388)  104 ((case-control* or case-based or case-comparison) adj (study or studies)).tw,kf. (98069)  105 Cross-Sectional Studies/ (290838)  106 (cross-section* or crosssection*).tw,kf. (341552)  107 or/92-106 [OBSERVATIONAL STUDY FILTER] (4505894)  108 65 and 107 [OBSERVATIONAL STUDIES] (4917)  109 73 or 91 or 108 [ALL STUDY DESIGNS - HARMS] (7367)  110 exp Animals/ not Humans/ (4568825)  111 109 not 110 [ANIMAL-ONLY REMOVED] (7061)  112 (comment or editorial or news or newspaper article).pt. (1301663)  113 (letter not (letter and randomized controlled trial)).pt. (1017400)  114 111 not (112 or 113) [OPINION PIECES REMOVED] (6894)  115 (2010* or 2011* or 2012* or 2013* or 2014* or 2015* or 2016* or 2017* or 2018* or 2019*).dt. (10150434)  116 114 and 115 (3507)  117 limit 114 to yr="2010-current" (3571)  118 116 or 117 [DATE LIMITS] (3594)  119 14 and 72 [DRUGS - RCT FILTER - BENEFITS] (6674)  120 119 not 110 [ANIMAL-ONLY REMOVED] (6278)  121 120 not (112 or 113) [OPINION PIECES REMOVED] (6098)  122 (2016* or 2017* or 2018* or 2019*).dt. (3974913)  123 121 and 122 (728)  124 limit 121 to yr="2016-Current" (771)  125 123 or 124 [ BENEFITS - DATE LIMITS] (789)  126 118 or 125 [BENEFITS OR HARMS - WITH DATE LIMITS] (3898)  127 Case Reports.pt. (1968151)  128 126 not 127 [CASE REPORTS REMOVED] (3764)  129 remove duplicates from 128 (3759) |
| --- |

**Ovid Embase**

| 1 alendronic acid/ (15491)  2 (alendronate or alenato$2 or alend$2 or alendros$2 or alovell$2 or arendal$2 or bifemelan$2 or bifosa$2 or binosto$2 or bonapex$2 or defixal$2 or dronal$2 or endronax$2 or eucalen$2 or fixopan$2 or fosalan$2 or fosamax$2 or fosmin$2 or fosval$2 or marvil$2 or maxibone$2 or "mk 0217" or "mk 217" or mk0217 or mk217 or neobon$2 or oncalst$2 or onclast$2 or osdron$2 or osdronat$2 or oseotenk$2 or osficar$2 or oslene$2 or osteofar$2 or osteofos$2 or osteopor$2 or osteosan$2 or osteovan$2 or osticalcin$2 or porosal$2 or teiroc$2 or tibolene$2 or voroste$2).tw,kw. (8424)  3 denosumab/ (7476)  4 (denosumab or "amg 162" or amg162 or amgiva$2 or prolia$2 or xgeva$2).tw,kw. (5044)  5 bisphosphonic acid derivative/ (32689)  6 (biphosphonate or biphosphonates or bisphosphonate or bisphosphonates or bisphosphonic acid derivative* or diphosphonate or diphosphonates or diphosphonic acid derivative*).tw,kw. (30286)  7 risedronic acid/ (7453)  8 (actonel$2 or atelvia$2 or benet$2 or "ne 58095" or ne58095 or optinate$2 or ribastamin$2 or risedronate or risedronic acid).tw,kw. (3534)  9 zoledronic acid/ (15043)  10 (aclasta$2 or "cgp 42446" or "cgp 42446a" or cgp42446 or cgp42446a or orazol$2 or reclast$2 or "zol 446" or zol446 or zoledronate or zoledronic acid or zomera$2 or zometa$2).tw,kw. (8636)  11 osteoporosis/dt [Drug Therapy] (19808)  12 postmenopause osteoporosis/dt [Drug Therapy] (6645)  13 fragility fracture/pc [Prevention] (1812)  14 or/1-13 [OSTEOPOROTIC DRUGS OF INTEREST, DRUG THERAPY/PREVENTION] (73198)  15 ae.fs. [adverse effects free-floating subheading] (1185073)  16 co.fs. [complications free-floating subheading] (1639483)  17 to.fs. [toxicity free-floating subheading] (511001)  18 "danger, risk, safety and related phenomena"/ (72)  19 exp adverse drug reaction/ (492295)  20 (ADR or ADRs or ADE or ADEs).tw,kw. (20653)  21 safety/ (251682)  22 exp patient safety/ (108351)  23 (safe* or unsafe*).tw,kw. (1178602)  24 (side effect* or tolerability or toxicity or treatment emergent or undesirable effect*).tw,kw. (866528)  25 (adverse adj2 (effect or effects or event or events or experience* or incident or incidents or outcome or outcomes or reaction or reactions)).tw,kw. (612840)  26 (injurious adj2 (effect or effects or event or events or experience* or incident or incidents or outcome or outcomes or reaction or reactions)).tw,kw. (1572)  27 (undesirable adj2 (effect or effects or event or events or experience* or incident or incidents or outcome or outcomes or reaction or reactions)).tw,kw. (9823)  28 risk/ (497469)  29 cardiovascular risk/ (184167)  30 fall risk/ (3652)  31 mortality risk/ (12217)  32 organs at risk/ (5873)  33 patient risk/ (5318)  34 risk*.ti. (606487)  35 (harm or harmed or harming or harms or harmful*).tw,kw. (136963)  36 (adverse* or complication* or injurious or treatment outcome* or undesirable or tolerability or interaction or interactions or reaction or reactions or tolerability or toxic).ti. (703171)  37 Patient Dropouts/ (769)  38 ((discontinu* or drop-out* or dropout* or withdr*) adj2 (participant* or patient* or subject*)).tw,kw. (34648)  39 or/15-38 [GENERAL HARMS, ADVERSE EVENTS TERMS] (6297990)  40 heart arrhythmia/ep, et [Epidemiology, Etiology] (8049)  41 exp atrial fibrillation/ep, et [Epidemiology, Etiology] (1411)  42 cardiovascular disease/ep, et [Epidemiology, Etiology] (35420)  43 exp colorectal tumor/ep, et [Epidemiology, Etiology] (2805)  44 exp esophagus tumor/ep, et [Epidemiology, Etiology] (8586)  45 exp femur fracture/ep, et [Epidemiology, Etiology] (1591)  46 gastrointestinal disease/ep, et [Epidemiology, Etiology] (4716)  47 jaw disease/ep, et [Epidemiology, Etiology] (1600)  48 bone necrosis/ep, et [Epidemiology, Etiology] (1515)  49 heart muscle ischemia/ep, et [Epidemiology, Etiology] (6940)  50 exp stomach tumor/ep, et [Epidemiology, Etiology] (16787)  51 cerebrovascular accident/ep, et [Epidemiology, Etiology] (8657)  52 (arrythmia* or arrhythmia* or dysrhythmia*).tw,kw. (128107)  53 ((atrial or atrium or auricular) adj fibril*).tw,kw. (112546)  54 ((cancer or cancers or carcinoma* or malignan* or metasta* or neoplasm* or tumor or tumors or tumour or tumours or adenoma* or adenocarcinoma* or adeno-carcinoma* or carcinosarcoma* or carcino-sarcoma*) adj3 (colon or colonic or colorectal* or colo-rectal* or esophag* or oesophag* or pharynx-esophag* or gastric or gastro* or rectal* or rectum or stomach)).tw,kw. (480187)  55 ((cardio* or cardiac or heart) adj2 (death* or disease* or event* or fatal* or incident? or mortalit*)).tw,kw. (603654)  56 ((complain* or effect* or symptom*) adj2 (gastric or gastro* or digesti* or stomach)).tw,kw. (47476)  57 ((femoral* or femur*) adj2 (break or breaks or breaking or broken or fracture*)).tw,kw. (20527)  58 ((infarct* or isch?emi*) adj1 (cardio* or cardiac or heart or myocardial or myo-cardial)).tw,kw. (352601)  59 heart attack?.tw,kw. (7456)  60 ((jaw or jaws or mandib*) adj3 (osteo-necro* or osteonecro*)).tw,kw. (3840)  61 (stroke or strokes or CVA or apople*).tw,kw. (368498)  62 ((brain or cerebral or cerebrum) adj (accident? or attack? or insult* or isch?emic attack? or vascular accident? or vascular insufficienc*)).tw,kw. (2962)  63 (cerebrovascular adj (accident? or arrest? or failure? or injury or injuries or insufficienc* or insult?)).tw,kw. (12915)  64 (isch?emic adj (cerebral attack? or seizure?)).tw,kw. (92)  65 or/40-64 [SPECIFIC DRUG HARMS] (1829965)  66 39 or 65 [GENERAL OR SPECIFIC DRUG HARMS] (7437463)  67 14 and 66 [DRUGS OF INTEREST - GENERAL AND SPECIFIC HARMS] (39509)  68 Bisphosphonate-Associated Osteonecrosis of the Jaw/ (975)  69 67 or 68 [DRUGS OF INTEREST - GENERAL AND SPECIFIC HARMS, SPECIFIC MESH] (39660)  70 exp randomized controlled trial/ or controlled clinical trial/ (726204)  71 clinical trial/ (956476)  72 exp "controlled clinical trial (topic)"/ (164052)  73 (randomi#ed or randomi#ation? or randomly or RCT or placebo*).tw,kw. (1255482)  74 ((singl* or doubl* or trebl* or tripl*) adj (mask* or blind* or dumm*)).tw,kw. (222636)  75 trial.ti. (265752)  76 or/70-75 [RCT FILTER] (2112521)  77 69 and 76 [RCTs] (12878)  78 controlled clinical trial/ (461653)  79 "controlled clinical trial (topic)"/ (9955)  80 (control* adj2 trial).tw,kw. (189426)  81 (nonrandom* or non-random* or quasi-random* or quasi-experiment*).tw,kw. (63285)  82 (nRCT or non-RCT).tw,kw. (395)  83 (control* adj3 ("before and after" or "before after")).tw,kw. (5122)  84 time series analysis/ (22708)  85 time series.tw,kw. (31003)  86 pretest posttest control group design/ (377)  87 (pre- adj3 post-).tw,kw. (129521)  88 (pretest adj3 posttest).tw,kw. (5633)  89 controlled study/ (6558379)  90 (control* adj2 study).tw,kw. (213862)  91 control group/ (110712)  92 (control* adj2 group?).tw,kw. (657465)  93 or/78-80 [NON-RCT FILTER] (608704)  94 69 and 93 [NON-RCTs] (3131)  95 cohort analysis/ (452892)  96 cohort?.tw,kw. (852478)  97 retrospective study/ (754210)  98 longitudinal study/ (123558)  99 prospective study/ (508843)  100 (longitudinal or prospective or retrospective).tw,kw. (1722075)  101 follow up/ (1379113)  102 ((followup or follow-up) adj (study or studies)).tw,kw. (63738)  103 observational study/ (163489)  104 (observation$2 adj (study or studies)).tw,kw. (144628)  105 population research/ (97303)  106 ((population or population-based) adj (study or studies or analys#s)).tw,kw. (22792)  107 ((multidimensional or multi-dimensional) adj (study or studies)).tw,kw. (136)  108 exp comparative study/ (1305263)  109 ((comparative or comparison) adj (study or studies)).tw,kw. (122523)  110 exp case control study/ (156071)  111 ((case-control* or case-based or case-comparison) adj (study or studies)).tw,kw. (126199)  112 cross-sectional study/ (293391)  113 (cross-section* or crosssection*).tw,kw. (438196)  114 or/95-113 [OBSERVATIONAL STUDY FILTER] (5257867)  115 69 and 114 [OBSERVATIONAL STUDIES] (10590)  116 77 or 94 or 115 [ALL STUDY DESIGNS - HARMS] (19641)  117 exp animal/ or exp animal experimentation/ or exp animal model/ or exp animal experiment/ or nonhuman/ or exp vertebrate/ (25651031)  118 exp human/ or exp human experimentation/ or exp human experiment/ (19492405)  119 117 not 118 (6159545)  120 116 not 119 [ANIMAL-ONLY REMOVED] (19251)  121 editorial.pt. (596174)  122 letter.pt. not (letter.pt. and randomized controlled trial/) (1050921)  123 120 not (121 or 122) [OPINION PIECES REMOVED] (18654)  124 (2010* or 2011* or 2012* or 2013* or 2014* or 2015* or 2016* or 2017* or 2018* or 2019*).dc. (13666859)  125 123 and 124 (10128)  126 limit 123 to yr="2010-current" (9612)  127 125 or 126 [DATE LIMITS] (10129)  128 14 and 76 [DRUGS - RCT FILTER - BENEFITS] (18356)  129 128 not 119 [ANIMAL-ONLY REMOVED] (17538)  130 129 not (121 or 122) [OPINION PIECES REMOVED] (16864)  131 (2016* or 2017* or 2018* or 2019*).dc. (5448365)  132 130 and 131 (2414)  133 limit 130 to yr="2016-Current" (2160)  134 132 or 133 [ BENEFITS - DATE LIMITS] (2436)  135 127 or 134 [BENEFITS OR HARMS - WITH DATE LIMITS] (11112)  136 case report/ (2335299)  137 135 not 136 [CASE REPORTS REMOVED] (10397)  138 conference abstract.pt. (3357973)  139 137 not 138 (6740)  140 137 and 138 (3657)  141 limit 140 to yr="2017-current" (700)  142 139 or 141 [MOST RECENT 2 YRS CONFERENCE ABSTRACTS RETAINED] (7440) |
| --- |

**Cochrane Library**

| ID Search Hits  #1 [mh Alendronate] 681  #2 (alendronate or alenato* or alend* or alendros* or alovell* or arendal* or bifemelan* or bifosa* or binosto* or bonapex* or defixal* or dronal* or endronax* or eucalen* or fixopan* or fosalan* or fosamax* or fosmin* or fosval* or marvil* or maxibone* or "mk 0217" or "mk 217" or mk0217 or mk217 or neobon* or oncalst* or onclast* or osdron* or osdronat* or oseotenk* or osficar* or oslene* or osteofar* or osteofos* or osteopor or osteosan* or osteovan* or osticalcin* or porosal* or teiroc* or tibolene* or voroste*):ti,ab,kw 1479  #3 [mh Denosumab] 228  #4 (denosumab or "amg 162" or amg162 or amgiva* or prolia* or xgeva*):ti,ab,kw 814  #5 [mh Diphosphonates] 2355  #6 (biphosphonate or biphosphonates or bisphosphonate or bisphosphonates or ("bisphosphonic acid" NEXT derivative*) or diphosphonate or diphosphonates or ("diphosphonic acid" NEXT derivative*)):ti,ab,kw 3124  #7 [mh "Risedronic Acid"] 238  #8 (actonel* or atelvia* or benet* or "ne 58095" or ne58095 or optinate* or ribastamin* or risedronate or "risedronic acid"):ti,ab,kw 690  #9 [mh "Zoledronic Acid"] 380  #10 (aclasta* or "cgp 42446" or "cgp 42446a" or cgp42446 or cgp42446a or orazol* or reclast* or "zol 446" or zol446 or zoledronate or "zoledronic acid" or zomera* or zometa*):ti,ab,kw 1477  #11 [mh Osteoporosis/DT] 1566  #12 [mh "Osteoporosis, Postmenopausal"/DT] 1045  #13 [mh "Osteoporotic Fractures"/PC] 119  #14 {or #1-#13} 6474  #15 [mh /AE] 122903  #16 [mh /CO] 51109  #17 [mh /TO] 1644  #18 [mh "Drug-Related Side Effects and Adverse Reactions"] 3274  #19 (ADR or ADRs or ADE or ADEs):ti,ab,kw 1349  #20 [mh ^Safety] 3011  #21 [mh "Patient Safety"] 492  #22 [mh "Patient Harm"] 2  #23 (safe* or unsafe*):ti,ab,kw 235102  #24 ((side NEXT effect*) or tolerability or toxicity or "treatment emergent" or (undesirable NEXT effect*)):ti,ab,kw 196465  #25 (adverse NEAR/2 (effect or effects or event or events or experience* or incident or incidents or outcome or outcomes or reaction or reactions)):ti,ab,kw 258791  #26 (injurious NEAR/2 (effect or effects or event or events or experience* or incident or incidents or outcome or outcomes or reaction or reactions)):ti,ab,kw 51  #27 (undesirable NEAR/2 (effect or effects or event or events or experience* or incident or incidents or outcome or outcomes or reaction or reactions)):ti,ab,kw 941  #28 [mh ^Risk] 3209  #29 risk*:ti 37634  #30 (harm or harmed or harming or harms or harmful*):ti,ab,kw 10982  #31 (adverse* or complication* or injurious or (treatment NEXT outcome*) or undesirable or tolerability or interaction or interactions or reaction or reactions or tolerability or toxic):ti 33952  #32 [mh "Patient Dropouts"] 1902  #33 ((discontinu* or drop-out* or dropout* or withdr*) NEAR/2 (participant* or patient* or subject*)):ti,ab,kw 13232  #34 {or #15-#33} 528005  #35 [mh "Osteoporotic Fractures"/CI] 6  #36 [mh ^"Arrhythmias, Cardiac"/EP,ET,CI] 867  #37 [mh "Atrial Fibrillation"/EP,ET,CI] 702  #38 [mh ^"Cardiovascular Diseases"/EP,ET,CI] 2768  #39 [mh "Colorectal Neoplasms"/EP,ET,CI] 366  #40 [mh "Esophageal Neoplasms"/EP,ET,CI] 48  #41 [mh "Femoral Fractures"/EP,ET,CI] 190  #42 [mh ^"Gastrointestinal Diseases"/EP,ET,CI] 870  #43 [mh ^"Jaw Diseases"/EP,ET,CI] 14  #44 [mh ^Osteonecrosis/EP,ET,CI] 42  #45 [mh "Myocardial Ischemia"/EP,ET,CI] 4315  #46 [mh "Stomach Neoplasms"/EP,ET,CI] 73  #47 [mh Stroke/EP,ET,CI] 1708  #48 (arrythmia* or arrhythmia* or dysrhythmia*):ti,ab,kw 11391  #49 ((atrial or atrium or auricular) NEXT fibril*):ti,ab,kw 11445  #50 ((cancer or cancers or carcinoma* or malignan* or metasta* or neoplasm* or tumor or tumors or tumour or tumours or adenoma* or adenocarcinoma* or adeno-carcinoma* or carcinosarcoma* or carcino-sarcoma*) NEAR/3 (colon or colonic or colorectal* or colo-rectal* or esophag* or oesophag* or pharynx-esophag* or gastric or gastro* or rectal* or rectum or stomach)):ti,ab,kw 32058  #51 ((cardio* or cardiac or heart) NEAR/2 (death* or disease* or event* or fatal* or incident* or mortalit*)):ti,ab,kw 61408  #52 ((complain* or effect* or symptom*) NEAR/2 (gastric or gastro* or digesti* or stomach)):ti,ab,kw 15380  #53 ((femoral* or femur*) NEAR/2 (break or breaks or breaking or broken or fracture*)):ti,ab,kw 2308  #54 ((infarct* or ischemi* or ischaemi*) NEAR/1 (cardio* or cardiac or heart or myocardial or myo-cardial)):ti,ab,kw 40503  #55 (heart NEXT (attack or attacks)):ti,ab,kw 1133  #56 ((jaw or jaws or mandib*) NEAR/3 (osteo-necro* or osteonecro*)):ti,ab,kw 312  #57 (stroke or strokes or CVA or apople*):ti,ab,kw 49926  #58 ((brain or cerebral or cerebrum) NEXT (accident* or attack or attacks or insult* or ischemic attack* or ischaemic attack* or vascular accident* or vascular insufficienc*)):ti,ab,kw 984  #59 (cerebrovascular NEXT (accident* or arrest* or failure* or injury or injuries or insufficienc* or insult*)):ti,ab,kw 12400  #60 ((ischemic or ischaemic) NEXT (cerebral attack* or seizure*)):ti,ab,kw 3260  #61 {or #35-#60} 182079  #62 #34 or #61 625229  #63 #14 and #62 3782  #64 [mh "Bisphosphonate-Associated Osteonecrosis of the Jaw"] 20  #65 #63 or #64 3782  #66 conference abstract:pt 143025  #67 #65 NOT #66 3280  #68 #65 AND #66 with Cochrane Library publication date Between Jan 2017 and Dec 2019 241  #69 #65 AND #66 with Publication Year from 2017 to 2019, in Trials 124  #70 #67 OR #68 OR #69 3521  #71 #67 OR #68 OR #69 with Cochrane Library publication date Between Jan 2010 and Dec 2019 2234  #72 #67 OR #68 OR #69 with Publication Year from 2010 to 2019, in Trials 1606  #73 #71 or #72 2229  #74 [mh Alendronate] 681  #75 (alendronate or alenato* or alend* or alendros* or alovell* or arendal* or bifemelan* or bifosa* or binosto* or bonapex* or defixal* or dronal* or endronax* or eucalen* or fixopan* or fosalan* or fosamax* or fosmin* or fosval* or marvil* or maxibone* or "mk 0217" or "mk 217" or mk0217 or mk217 or neobon* or oncalst* or onclast* or osdron* or osdronat* or oseotenk* or osficar* or oslene* or osteofar* or osteofos* or osteopor or osteosan* or osteovan* or osticalcin* or porosal* or teiroc* or tibolene* or voroste*):ti,ab,kw 1479  #76 [mh Denosumab] 228  #77 (denosumab or "amg 162" or amg162 or amgiva* or prolia* or xgeva*):ti,ab,kw 814  #78 [mh Diphosphonates] 2355  #79 (biphosphonate or biphosphonates or bisphosphonate or bisphosphonates or ("bisphosphonic acid" NEXT derivative*) or diphosphonate or diphosphonates or ("diphosphonic acid" NEXT derivative*)):ti,ab,kw 3124  #80 [mh "Risedronic Acid"] 238  #81 (actonel* or atelvia* or benet* or "ne 58095" or ne58095 or optinate* or ribastamin* or risedronate or "risedronic acid"):ti,ab,kw 690  #82 [mh "Zoledronic Acid"] 380  #83 (aclasta* or "cgp 42446" or "cgp 42446a" or cgp42446 or cgp42446a or orazol* or reclast* or "zol 446" or zol446 or zoledronate or "zoledronic acid" or zomera* or zometa*):ti,ab,kw 1477  #84 [mh Osteoporosis/DT] 1566  #85 [mh "Osteoporosis, Postmenopausal"/DT] 1045  #86 [mh "Osteoporotic Fractures"/PC] 119  #87 {or #74-#86} 6474  #88 conference abstract:pt 143025  #89 #87 NOT #88 5596  #90 #87 AND #88 with Cochrane Library publication date Between Jan 2017 and Dec 2019 407  #91 #87 AND #88 with Publication Year from 2017 to 2019, in Trials 215  #92 #89 OR #90 OR #91 6003  #93 #89 OR #90 OR #91 with Cochrane Library publication date Between Jan 2016 and Dec 2019 2619  #94 #89 OR #90 OR #91 with Publication Year from 2016 to 2019, in Trials 1046  #95 #93 OR #94 2619  #96 #73 OR #95 3380  DSR – 21 Reviews, 7 Protocols  CENTRAL – 3345 Trials |
| --- |

**Key Question 4:** Acceptability of screening

**Ovid MEDLINE(R) and Epub Ahead of Print, In-Process & Other Non-Indexed Citations and Daily 1946 to May 23, 2019, Embase 1974 to present, PsycINFO 2002 to present**

| 1 Bone Density/ (137823)  2 Decalcification, Pathologic/ (108406)  3 Fractures, Bone/ (84790)  4 Osteoporosis/ (151267)  5 Osteoporosis, Postmenopausal/ (17627)  6 Osteoporotic Fractures/ (11605)  7 ((bone? or hip or hips) adj2 (break* or broken or fracture*)).tw,kf. (73687)  8 (bone* adj2 (brittle* or fragil*)).tw,kf. (6965)  9 (bone* adj2 (content* or densit* or loss* or mass)).tw,kf. (200746)  10 (bone* adj1 health*).tw,kf. (18499)  11 (fracture* adj2 fragil*).tw,kf. (9742)  12 osteoporo*.tw,kf. (181661)  13 Bone Density Conservation Agents/ (15879)  14 Bone Resorption.tw,kf. (55214)  15 (antiresorptive or anti-resorptive).tw,kf. (7779)  16 (antiosteoporo* or anti-osteoporo*).tw,kf. (5178)  17 or/1-16 [Combined MeSH & text words for fragility fractures, osteoporosis, general anti-osteoporotic agents] (525122)  18 Adolescent/ not exp Adult/ (1108389)  19 exp Child/ not exp Adult/ (2955307)  20 exp Infant/ not exp Adult/ (1542487)  21 17 not (18 or 19 or 20) [ADOLESCENT-, CHILD-, INFANT-ONLY REMOVED] (493474)  22 Osteoporosis/di, dt, pc, th [diagnosis, drug therapy, prevention & control, therapy] (52577)  23 Osteoporosis, Postmenopausal/di, dt, pc, th [diagnosis, drug therapy, prevention & control, therapy] (11978)  24 Osteoporotic Fractures/di, dt, pc, th [diagnosis, drug therapy, prevention & control, therapy] (4210)  25 Fractures, Bone/pc [prevention & control] (4667)  26 (osteoporo* adj3 (diagnos* or drug? or medicat* or prevent* or screen* or therap* or treat*)).tw,kf. (56670)  27 (fracture? adj3 prevent*).tw,kf. (12472)  28 or/22-27 [GENERAL VOCABULARY FOR OSTEOPOROSIS DIAGNOSIS, PREVENTION, TREATMENT] (105534)  29 Early Diagnosis/ (123848)  30 Mass Screening/ (149309)  31 (screen* or detect*).tw,kf. (6341353)  32 (identif* or recogni*).ti. (802150)  33 ((early or earlier or earliest) adj5 (diagnos* or identif* or recogni*)).tw,kf. (463670)  34 (case finding? or casefinding?).tw,kf. (11436)  35 or/29-34 [GENERAL SCREENING] (7337757)  36 Absorptiometry, Photon/ (24569)  37 Algorithms/ (418999)  38 Risk/ (616502)  39 Risk Assessment/ (762231)  40 "Risk Evaluation and Mitigation"/ (224)  41 Risk Factors/ (1398030)  42 a#sorptiometr*.tw,kf. (56846)  43 (absolute adj2 risk?).tw,kf. (17340)  44 algorithm*.tw,kf. (530376)  45 ((assess* or calculat* or estimat* or predict*) adj3 risk?).tw,kf. (498920)  46 ((assess* or predict* or prognos*) adj2 (instrument* or test* or tool*)).tw,kf. (301511)  47 ((BMD or bone mineral dens*) adj3 test*).tw,kf. (3122)  48 (bone* adj3 (densitometr* or photodensitometr* or photo-densitometr*)).tw,kf. (6882)  49 ((densitometr* or photodensitometr* or photo-densitometr*) adj3 (radiograph* or x-ray* or xray*)).tw,kf. (1448)  50 ((DEXA or DXA) adj2 scan*).tw,kf. (7530)  51 ((factor* or index* or indices* or scale* or scor* or stratif* or test*) adj3 risk?).tw,kf. (1607437)  52 (fractur* adj3 (predict* or risk*)).tw,kf. (53170)  53 (fractureindex or fracture index).tw,kf. (224)  54 FRAX.tw,kf. (4161)  55 GARVAN.tw,kf. (156)  56 ((predict* or prognos*) adj2 score*).tw,kf. (52809)  57 (scoring adj2 system?).tw,kf. (77375)  58 ((predict* or prognos*) adj2 model*).tw,kf. (219923)  59 Qfracture*.tw,kf. (124)  60 (relative adj2 risk?).tw,kf. (173629)  61 (predict* adj2 (guide or guides or rule or rules)).tw,kf. (8262)  62 or/36-61 [RISK ASSESSMENT, INCLUDING BMD TESTS] (4858013)  63 28 or 35 or 62 (11398200)  64 21 and 63 [OSTEOPOROSIS/FRAGILITY FRACTURES - DIAGNOSIS, PREVENTION & SCREENING, TREATMENT, RISK] (219513)  65 Choice Behavior/ (223754)  66 (choice? adj2 behavio?r*).tw,kf. (9707)  67 Cooperative Behavior/ (76949)  68 Decision Making/ (351866)  69 decision aid*.tw,kf. (7573)  70 ((guide? or guiding or make or making or makes or made or shar* or support*) adj2 (choice? or choos* or consent* or decid* or decision*)).tw,kf. (496734)  71 Patient Education as Topic/ (171958)  72 Patient Acceptance of Health Care/ (96800)  73 Patient Participation/ (50749)  74 Patient Preference/ (23083)  75 Patient Satisfaction/ (209334)  76 ((involv* or participat*) adj3 (patient? or person$2 or personally or man or men or "man's" or "men's" or wom#n or "woman's" or "women's")).tw,kf. (300787)  77 exp Patients/px [psychology] (16036)  78 Uncertainty/ (40092)  79 ((accept* or consider* or choice? or choos* or chose? or decid* or decis* or expect* or input* or knowledge* or opinion* or participat* or prefer* or respons* or satisf* or uncertain* or understand*) adj2 (female? or male? or man or men or "man's" or "men's" or patient? or person$2 or personally or wom#n or "woman's" or "women's")).tw,kf. (819604)  80 ((analys#s or valuation? or value? or valuing) adj2 (conjoint or contingent)).tw,kf. (4226)  81 (choice? adj1 (discrete or experiment*)).tw,kf. (8226)  82 ((patient? or person$2 or personally or man or men or "man's" or "men's" or wom#n or "woman's" or "women's") adj (centered or centred or focus*)).tw,kf. (75077)  83 Informed Consent/ (138641)  84 (informed adj (choice* or choos* or consent* or decid* or decision*)).tw,kf. (136205)  85 ((patient? or person$2 or personally or man or men or "man's" or "men's" or wom#n or "woman's" or "women's") adj2 consent*).tw,kf. (33367)  86 ((inten* or refus* or reject* or uptake or willing*) adj2 (initiat* or intervention? or screen* or therap* or treat*)).tw,kf. (215800)  87 (preference? adj1 (elicit* or reveal* or scor* or stated)).tw,kf. (5191)  88 trade?off?.tw,kf. (14789)  89 Information Seeking Behavior/ (4913)  90 (inform* adj1 seek*).tw,kf. (11897)  91 or/65-90 [PATIENT PARTICIPATION/DECISION-MAKING] (2573707)  92 64 and 91 (13869)  93 exp Animals/ not Humans/ (17593595)  94 (animal or animal-model* or animals or canine* or cat or cats or dog or dogs or feline or felines or hamster or hamsters or mice or monkey or monkeys or mouse or pig or piglet or piglets or pigs or porcine or primate* or rabbit or rabbits or rat or rats or rodent or rodents or sheep or swine or swines).ti. (4248085)  95 92 not (93 or 94) [ANIMAL-ONLY REMOVED] (9433)  96 ((case report* or case study or case studies) not ((lit* or systematic*) adj2 review*)).ti. (585113)  97 (case reports or comment or editorial or news or newspaper article).pt. (3920061)  98 95 not (96 or 97) [OPINION PIECES AND CASE STUDIES REMOVED] (9126)  99 limit 98 to yr="2005-Current" (6048)  100 limit 99 to (english or french) (5678)  101 100 use ppez [MEDLINE RECORDS] (3247)  102 exp bone density/ (138037)  103 fracture/ (78538)  104 fragility fracture/ (16560)  105 pathologic fracture/ (15204)  106 osteoporosis/ (151267)  107 postmenopause osteoporosis/ (13751)  108 ((bone? or hip or hips) adj2 (break* or broken or fracture*)).tw,kw. (73963)  109 (bone* adj2 (brittle* or fragil*)).tw,kw. (7047)  110 (bone* adj2 (content* or densit* or loss* or mass)).tw,kw. (201836)  111 (bone* adj1 health*).tw,kw. (18671)  112 (fracture* adj2 fragil*).tw,kw. (9851)  113 osteoporo*.tw,kw. (188221)  114 bone density conservation agent/ (16119)  115 bone resorption.tw,kw. (56107)  116 (antiresorptive or anti-resorptive).tw,kw. (7866)  117 (antiosteoporo* or anti-osteoporo*).tw,kw. (5226)  118 or/102-117 [Combined MeSH & text words for fragility fractures, osteoporosis, general anti-osteoporotic agents] (513775)  119 exp adolescent/ not exp adult/ (1108531)  120 exp child/ not exp adult/ (2955307)  121 fetus/ not exp adult/ (219885)  122 118 not (119 or 120 or 121) [ADOLESCENT-, CHILD-, INFANT-ONLY REMOVED] (485211)  123 osteoporosis/di, dt, pc, th [diagnosis, drug therapy, prevention & control, therapy] (52577)  124 postmenopause osteoporosis/di, dt, pc, th [diagnosis, drug therapy, prevention & control, therapy] (8670)  125 fragility fracture/di, dt, pc, th [diagnosis, drug therapy, prevention & control, therapy] (3441)  126 fracture/pc [prevention & control] (3668)  127 (osteoporo* adj3 (diagnos* or drug? or medicat* or prevent* or screen* or therap* or treat*)).tw,kw. (56947)  128 (fracture? adj3 prevent*).tw,kw. (12549)  129 or/123-128 [GENERAL VOCABULARY FOR OSTEOPOROSIS DIAGNOSIS, PREVENTION, TREATMENT] (103226)  130 early diagnosis/ (123848)  131 mass screening/ (149309)  132 (screen* or detect*).tw,kw. (6351737)  133 (identif* or recogni*).ti. (802150)  134 ((early or earlier or earliest) adj5 (diagnos* or identif* or recogni*)).tw,kw. (464684)  135 case finding/ (3090)  136 (case finding? or casefinding?).tw,kw. (11563)  137 or/130-136 [GENERAL SCREENING] (7348461)  138 photon absorptiometry/ (25205)  139 algorithm/ (495628)  140 risk/ (616502)  141 fall risk/ (3732)  142 fall risk assessment/ (1517)  143 risk assessment/ (762231)  144 "risk evaluation and mitigation strategy"/ (210)  145 risk factor/ (1785595)  146 a#sorptiometr*.tw,kw. (57241)  147 (absolute adj2 risk?).tw,kw. (17387)  148 algorithm*.tw,kw. (532738)  149 ((assess* or calculat* or estimat* or predict*) adj3 risk?).tw,kw. (503374)  150 ((assess* or predict* or prognos*) adj2 (instrument* or test* or tool*)).tw,kw. (302220)  151 ((BMD or bone mineral dens*) adj3 test*).tw,kw. (3143)  152 (bone* adj3 (densitometr* or photodensitometr* or photo-densitometr*)).tw,kw. (7396)  153 ((densitometr* or photodensitometr* or photo-densitometr*) adj3 (radiograph* or x-ray* or xray*)).tw,kw. (1493)  154 ((DEXA or DXA) adj2 scan*).tw,kw. (7573)  155 ((factor* or index* or indices* or scale* or scor* or stratif* or test*) adj3 risk?).tw,kw. (1620079)  156 (fractur* adj3 (predict* or risk*)).tw,kw. (53377)  157 (fractureindex or fracture index).tw,kw. (227)  158 FRAX.tw,kw. (4234)  159 GARVAN.tw,kw. (158)  160 ((predict* or prognos*) adj2 score*).tw,kw. (52962)  161 (scoring adj2 system?).tw,kw. (77719)  162 ((predict* or prognos*) adj2 model*).tw,kw. (220821)  163 Qfracture*.tw,kw. (127)  164 (relative adj2 risk?).tw,kw. (173864)  165 (predict* adj2 (guide or guides or rule or rules)).tw,kw. (8353)  166 or/138-165 [RISK ASSESSMENT, INCLUDING BMD TESTS] (4997457)  167 129 or 137 or 166 (11526033)  168 122 and 167 [OSTEOPOROSIS/FRAGILITY FRACTURES - DIAGNOSIS, PREVENTION & SCREENING, TREATMENT, RISK] (225232)  169 exp cooperation/ (66450)  170 (choice? adj2 behavio?r*).tw,kw. (9856)  171 decision making/ (351866)  172 patient decision making/ (9376)  173 shared decision making/ (93158)  174 decision aid*.tw,kw. (7752)  175 ((guide? or guiding or make or making or makes or made or shar* or support*) adj2 (choice? or choos* or consent* or decid* or decision*)).tw,kw. (499723)  176 patient education/ (192005)  177 patient attitude/ (73721)  178 patient participation/ (50749)  179 patient preference/ (23083)  180 patient satisfaction/ (209334)  181 ((involv* or participat*) adj3 (patient? or person$2 or personally or man or men or "man's" or "men's" or wom#n or "woman's" or "women's")).tw,kw. (301165)  182 uncertainty/ (40092)  183 ((accept* or consider* or choice? or choos* or chose? or decid* or decis* or expect* or input* or knowledge* or opinion* or participat* or prefer* or respons* or satisf* or uncertain* or understand*) adj2 (female? or male? or man or men or "man's" or "men's" or patient? or person$2 or personally or wom#n or "woman's" or "women's")).tw,kw. (821536)  184 ((analys#s or valuation? or value? or valuing) adj2 (conjoint or contingent)).tw,kw. (4354)  185 (choice? adj1 (discrete or experiment*)).tw,kw. (8285)  186 ((patient? or person$2 or personally or man or men or "man's" or "men's" or wom#n or "woman's" or "women's") adj (centered or centred or focus*)).tw,kw. (75712)  187 informed consent/ (138641)  188 (informed adj (choice* or choos* or consent* or decid* or decision*)).tw,kw. (137238)  189 ((patient? or person$2 or personally or man or men or "man's" or "men's" or wom#n or "woman's" or "women's") adj2 consent*).tw,kw. (33403)  190 ((inten* or refus* or reject* or uptake or willing*) adj2 (initiat* or intervention? or screen* or therap* or treat*)).tw,kw. (216693)  191 (preference? adj1 (elicit* or reveal* or scor* or stated)).tw,kw. (5234)  192 trade?off?.tw,kw. (15660)  193 information seeking/ (5143)  194 (inform* adj1 seek*).tw,kw. (11941)  195 or/169-194 [PATIENT PARTICIPATION/DECISION-MAKING] (2530765)  196 168 and 195 (14623)  197 exp animal/ or exp animal experimentation/ or exp animal model/ or exp animal experiment/ or nonhuman/ or exp vertebrate/ (48393205)  198 exp human/ or exp human experimentation/ or exp human experiment/ (37427143)  199 197 not 198 (10967608)  200 (animal or animal-model* or animals or canine* or cat or cats or dog or dogs or feline or felines or hamster or hamsters or mice or monkey or monkeys or mouse or pig or piglet or piglets or pigs or porcine or primate* or rabbit or rabbits or rat or rats or rodent or rodents or sheep or swine or swines).ti. (4248085)  201 196 not (199 or 200) [ANIMAL-ONLY REMOVED] (14519)  202 ((case report* or case study or case studies) not ((lit* or systematic*) adj2 review*)).ti. (585113)  203 case report/ (4376260)  204 editorial.pt. (1092609)  205 201 not (202 or 203 or 204) [OPINION PIECES AND CASE STUDIES REMOVED] (13848)  206 limit 205 to yr="2005-Current" (10845)  207 limit 206 to (english or french) (10353)  208 conference abstract.pt. (3412872)  209 207 not 208 [CONFERENCE ABSTRACTS REMOVED] (7505)  210 207 and 208 (2848)  211 limit 210 to yr="2017-current" (565)  212 209 or 211 [MOST RECENT 2 YRS CONFERENCE ABSTRACTS RETAINED] (8070)  213 212 use oemezd [EMBASE RECORDS] (5009)  214 Osteoporosis/ (151267)  215 ((bone? or hip or hips) adj2 (break* or broken or fracture*)).tw. (72303)  216 (bone* adj2 (brittle* or fragil*)).tw. (6902)  217 (bone* adj2 (content* or densit* or loss* or mass)).tw. (200022)  218 (bone* adj1 health*).tw. (18398)  219 (fracture* adj2 fragil*).tw. (9611)  220 osteoporo*.tw. (179227)  221 bone resorption.tw. (54835)  222 (antiresorptive or anti-resorptive).tw. (7741)  223 (antiosteoporo* or anti-osteoporo*).tw. (5165)  224 or/214-223 [Combined MeSH & text words for fragility fractures, osteoporosis, general anti-osteoporotic agents] (430599)  225 (osteoporo* adj3 (diagnos* or drug? or medicat* or prevent* or screen* or therap* or treat*)).tw. (56555)  226 (fracture? adj3 prevent*).tw. (12401)  227 or/225-226 [GENERAL VOCABULARY FOR OSTEOPOROSIS DIAGNOSIS, PREVENTION, TREATMENT] (64298)  228 Screening/ (280066)  229 Health Screening/ (54609)  230 Screening Tests/ (24570)  231 (screen* or detect*).tw. (6334341)  232 (identif* or recogni*).ti. (802150)  233 ((early or earlier or earliest) adj5 (diagnos* or identif* or recogni*)).tw. (462970)  234 (case finding? or casefinding?).tw. (11377)  235 or/228-234 [GENERAL SCREENING] (7321011)  236 Algorithms/ (418999)  237 Risk Assessment/ (762231)  238 Risk Factors/ (1398030)  239 a#sorptiometr*.tw. (56622)  240 (absolute adj2 risk?).tw. (17309)  241 algorithm*.tw. (529105)  242 ((assess* or calculat* or estimat* or predict*) adj3 risk?).tw. (495278)  243 ((assess* or predict* or prognos*) adj2 (instrument* or test* or tool*)).tw. (301093)  244 ((BMD or bone mineral dens*) adj3 test*).tw. (3121)  245 (bone* adj3 (densitometr* or photodensitometr* or photo-densitometr*)).tw. (6824)  246 ((densitometr* or photodensitometr* or photo-densitometr*) adj3 (radiograph* or x-ray* or xray*)).tw. (1446)  247 ((DEXA or DXA) adj2 scan*).tw. (7506)  248 ((factor* or index* or indices* or scale* or scor* or stratif* or test*) adj3 risk?).tw. (1598911)  249 (fractur* adj3 (predict* or risk*)).tw. (53098)  250 (fractureindex or fracture index).tw. (224)  251 FRAX.tw. (4137)  252 GARVAN.tw. (155)  253 ((predict* or prognos*) adj2 score*).tw. (52677)  254 (scoring adj2 system?).tw. (77209)  255 ((predict* or prognos*) adj2 model*).tw. (219199)  256 Qfracture*.tw. (123)  257 (relative adj2 risk?).tw. (173444)  258 (predict* adj2 (guide or guides or rule or rules)).tw. (8130)  259 or/236-258 [RISK ASSESSMENT, INCLUDING BMD TESTS] (4432079)  260 227 or 235 or 259 (11009380)  261 224 and 260 [OSTEOPOROSIS/FRAGILITY FRACTURES - DIAGNOSIS, PREVENTION & SCREENING, TREATMENT, RISK] (185915)  262 exp Choice Behavior/ (412106)  263 (choice? adj2 behavio?r*).tw. (9593)  264 Cooperation/ (50341)  265 Decision Making/ (351866)  266 Group Decision Making/ (1311)  267 decision aid*.tw. (7432)  268 ((guide? or guiding or make or making or makes or made or shar* or support*) adj2 (choice? or choos* or consent* or decid* or decision*)).tw. (491431)  269 Client Education/ (2106)  270 Client Participation/ (1649)  271 exp Client Attitudes/ (13709)  272 ((involv* or participat*) adj3 (patient? or person$2 or personally or man or men or "man's" or "men's" or wom#n or "woman's" or "women's")).tw. (300246)  273 exp Uncertainty/ (40499)  274 ((accept* or consider* or choice? or choos* or chose? or decid* or decis* or expect* or input* or knowledge* or opinion* or participat* or prefer* or respons* or satisf* or uncertain* or understand*) adj2 (female? or male? or man or men or "man's" or "men's" or patient? or person$2 or personally or wom#n or "woman's" or "women's")).tw. (817657)  275 ((analys#s or valuation? or value? or valuing) adj2 (conjoint or contingent)).tw. (4123)  276 (choice? adj1 (discrete or experiment*)).tw. (8159)  277 ((patient? or person$2 or personally or man or men or "man's" or "men's" or wom#n or "woman's" or "women's") adj (centered or centred or focus*)).tw. (73261)  278 Informed Consent/ (138641)  279 (informed adj (choice* or choos* or consent* or decid* or decision*)).tw. (135454)  280 ((patient? or person$2 or personally or man or men or "man's" or "men's" or wom#n or "woman's" or "women's") adj2 consent*).tw. (33359)  281 ((inten* or refus* or reject* or uptake or willing*) adj2 (initiat* or intervention? or screen* or therap* or treat*)).tw. (215539)  282 (preference? adj1 (elicit* or reveal* or scor* or stated)).tw. (5114)  283 trade?off?.tw. (14695)  284 Information Seeking/ (5143)  285 (inform* adj1 seek*).tw. (11717)  286 or/262-285 [PATIENT PARTICIPATION/DECISION-MAKING] (2308232)  287 261 and 286 (11129)  288 limit 287 to yr="2005-Current" (8725)  289 limit 288 to (english or french) (8294)  290 289 use ppez,oemezd (8131)  291 289 not 290 [PSYCINFO RECORDS] (163)  292 101 or 213 or 291 [ALL DATABASES] (8419)  293 limit 292 to yr="2015-current" (3337)  294 remove duplicates from 293 (2391)  295 292 not 293 (5082)  296 remove duplicates from 295 (3379)  297 294 or 296 [TOTAL UNIQUE RECORDS] (5770)  298 297 use ppez [MEDLINE UNIQUE RECORDS] (3239)  299 297 use oemezd [EMBASE UNIQUE RECORDS] (2478)  300 297 not (298 or 299) [PSYCINFO UNIQUE RECORDS] (53) |
| --- |
